# Effects of Ligand N and O Donor Content on the Structure, Aqueous Stability, and SOD Activity of a Series of Mn^2+^ Complexes with Pyridine-Containing Tripodal Ligands

**DOI:** 10.1021/acsomega.5c10215

**Published:** 2025-11-14

**Authors:** Steven T. Frey, Haley A. Cirka, Jasper G. Ballot, Katheryn C. Rinaolo, Nich Nearyrat Phalkun, Anthony Mirkovic, Aidan Spengler, Avery J. Stamps, Jacob R. Hale, Yuta Nakagawa, Hanyu Ruan, Haiyang Lin, Ian T. Frey, Samuel J. Frey, Peter J. Bonitatibus

**Affiliations:** † Department of Chemistry, 7230 Skidmore College, 815 North Broadway, Saratoga Springs, New York 12866, United States; ‡ Department of Chemistry, 8024 Rensselaer Polytechnic Institute, 110 Eighth Street, Troy, New York 12180, United States

## Abstract

A series of six Mn^2+^ complexes of pyridine-containing, tripodal ligands were investigated in this study to examine the effect of varying ligand N and O donor content on their structural, aqueous stability and superoxide dismutase (SOD) activity. Crystalline forms of the complexes of the formula [MnL­(OAc)­(MeOH)]­BPh_4_, where L is a tripodal ligand, were characterized by IR, elemental analysis, and X-ray diffraction. Crystal structures of each compound reveal a hepta-coordinated Mn^2+^ ion with distorted pentagonal bipyramidal geometry. Complex formation in aqueous solution was examined by potentiometric titration and cyclic voltammetry. Mn^2+^-binding affinities (log β) of the ligands are strongly influenced by the N and O donor content of the ligands, with greater N content resulting in higher stability. Reduction potentials, *E*_1/2_ Mn­(III/II) of the complexes also correlate to the N and O donor content of the ligands, with lower O content producing higher *E*_1/2_ values. The aqueous complexes catalyze the efficient disproportionation of superoxide ion, where the apparent catalytic rate constants (*k*_cat_) are influenced by the nature of the O-donor moiety (−OH vs –OCH_3_). For complexes with methoxy-containing ligands, increasing –OCH_3_ content correlates negatively with *k*_cat_ values. The opposite trend is observed for complexes with hydroxy-containing ligands, suggesting the role of hydrogen bonding and/or proton transfer by –OH groups in the catalytic mechanism of these complexes.

## Graphical abstract

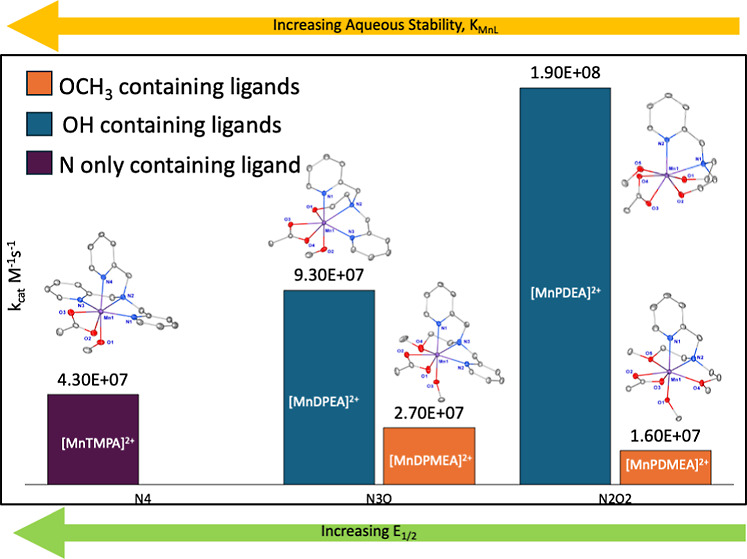

## Introduction

Reactive oxygen species (RNOS), which include hydroxyl radical (OH^•^), superoxide ion (O_2_^•–^), peroxide (O_2_^2–^), nitric oxide (NO^•^), and others have important roles in biological systems such as cell signaling and homeostasis.^1^ Yet, their misregulation can lead to oxidative damage of cells and impairment of cell signaling. Enhanced ROS production accompanies tissue damage from injury or a variety of inflammatory diseases including cancer, diabetes, cardiovascular diseases, and atherosclerosis.^2^ Organisms have therefore evolved protective mechanisms that includes antioxidant enzymes to regulate RNOS.^3–5^ Superoxide dismutases (SODs) are a family of these enzymes that contain either Mn, Fe, Ni, or a combination of Cu and Zn metal centers.^3,6^ These enzymes are capable of rapidly converting superoxide ion to molecular oxygen and hydrogen peroxide using a ping-pong mechanism during which the redox-active metal of the center is alternately oxidized and reduced.^6^

A growing number of biomimetic compounds and materials have been studied as potential therapeutics that might augment the natural regulation of ROS during disease states.^7^ While these include iron and copper compounds, an emphasis has been placed on manganese compounds due to the lower toxicity of manganese than iron or copper.^8–14^ Unfortunately, Mn^2+^ complexes are generally less stable than Fe^2+^ or Cu^2+^ complexes in aqueous solution, as illustrated by the Irving-Williams series.^15,16^ Hence, a thorough understanding of the factors that lead to both aqueous stability and catalytic activity are critical in the of design manganese compounds with therapeutic potential.

The metal binding site of MnSOD enzymes consists of three histidine nitrogens and an aspartate oxygen that coordinate the manganese ion, along with a water molecule, in a distorted trigonal bipyramidal geometry.^17,18^ This combination of N- and O-donor atoms, the geometry of the coordination sphere, and the hydrogen bonding network in the active site, presumably stabilize the manganese ion and permit it to alternate between the +2 and +3 oxidation states in the catalytic disproportionation of superoxide ion shown in Scheme 1.^19^

**Scheme 1:**
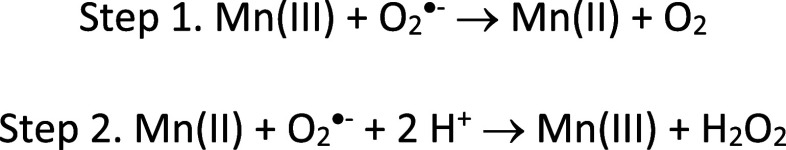
Ping Pong Mechanism for the Disproportionation of Superoxide Ion, O_2_^•–^ Into Molecular Oxygen and Hydrogen Peroxide by MnSODs

The well-defined active site structure of MnSOD enzymes has inspired the synthesis and examination of a number of small molecular weight analogs.^8–14^ These biomimetic compounds have, in turn, contributed important information regarding the factors that optimize the SOD activity of coordinated manganese ions.^10^ The study of new biomimetic compounds with systematic variations is likely to further enhance our understanding of how to stabilize Mn­(II/III) complexes in aqueous solution and optimize their antioxidant behavior. We therefore initiated a study of Mn^2+^ complexes with tripodal ligands that provide a variety of N- and O-donor moieties, and thus the opportunity to probe the effects of donor atom types and related functionalities on aqueous stability and SOD activity. Herein we describe Mn^2+^ complex formation of the six ligands shown in Figure 1, along with their structural characterization, range of stability in aqueous solution from weak to moderate, and efficient SOD-like reactivity, that lead to new insights for the design of Mn^2+^ compounds for therapeutic use.

**Figure 1:**
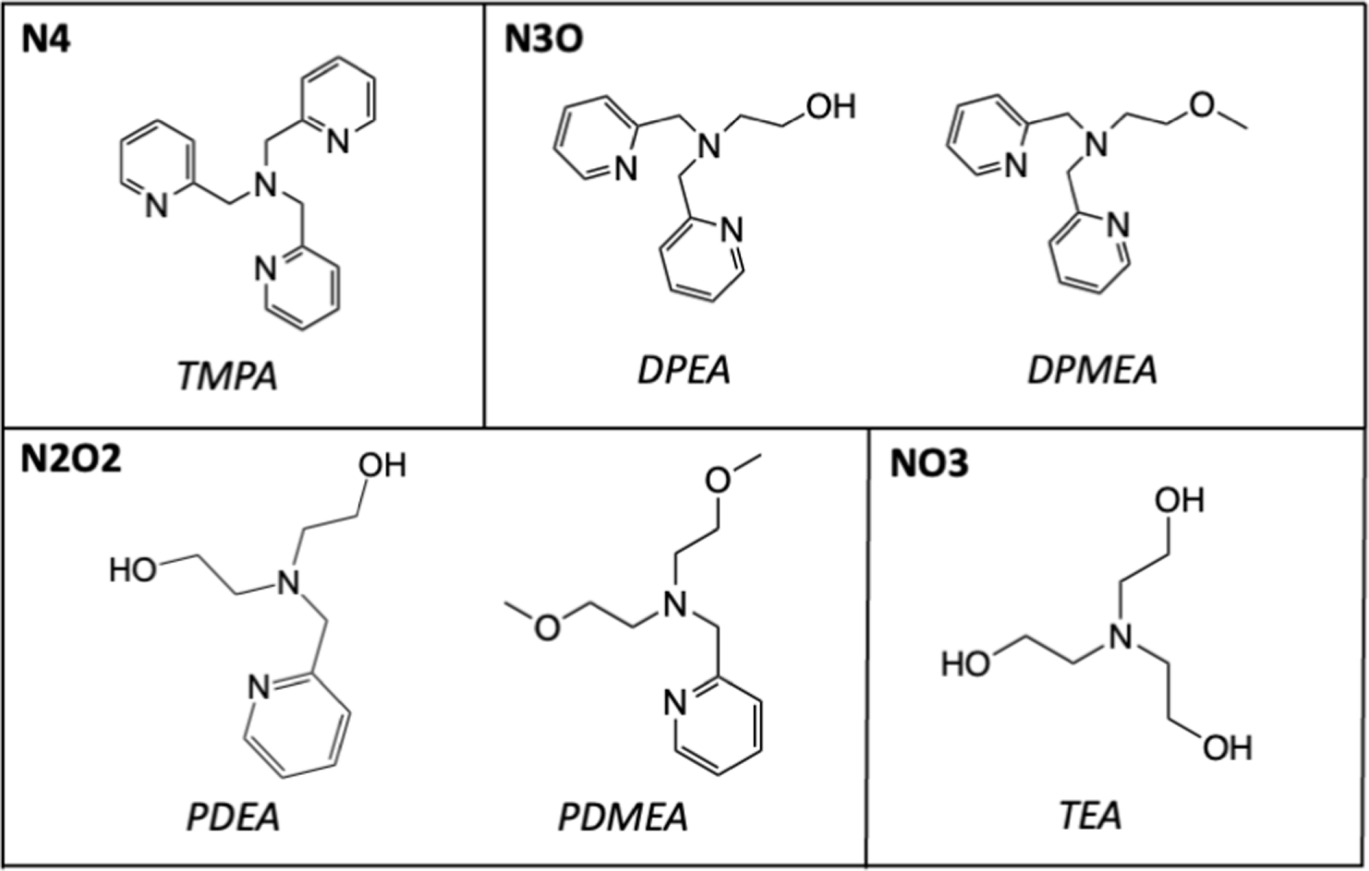
Structures of the six tripodal ligands described in this study, grouped by the number of N- and O-donor atoms that they contain.

## Experimental Methods

### Materials and Instrumentation

Reagents and solvents were obtained from commercial sources and used without further purification. The water used was deionized using a Barnstead NANOpure water purification system providing 18 MΩ cm^–1^ resistance, hereafter referred to as DI H_2_O. UV–vis experiments were carried out on an Agilent Technologies Cary 60 spectrometer equipped with a Peltier cell holder for temperature-controlled experiments. IR spectra were recorded using a PerkinElmer Spectrum 100 FT-IR. Both ^1^H and ^13^C NMR spectra were recorded on a JEOL JNM-ECZ400s NMR spectrometer and referenced against TMS. LC–MS analyses were run on a Thermo Vanquish LC equipped with an ISQ-ESI mass spectrometer. Cyclic voltammograms were acquired with an EDAQ 466 potentiostat and EChem software. Potentiometric titrations were recorded using a Thermo Scientific Orion Star T910 Series Titrator, and fit with GLEE^20^ and HyperQuad2013 ^21^ software.

### Ligand Syntheses

Triethanolamine (**TEA**) was obtained commercially and used without further purification.

#### Tris­(pyridin-2-ylmethyl)­amine (**TMPA**)

TMPA was synthesized and purified as described previously.^22^ ^1^H NMR (400 MHz, CDCl_3_) δ 8.54 (d, *J* = 4.8 Hz, 3H), 7.66 (t, *J* = 7.6 Hz, 3H), 7.59 (d, *J* = 6.6 Hz, 3H), 7.14 (t, *J* = 6.3 Hz, 3H), 3.89 (s, 6H). ^13^C NMR (101 MHz, CDCl_3_) δ 159.43, 149.14, 136.42, 122.94, 122.00, 60.17. IR (ATR, cm^–1^): 3081–2708 (m, ν_C–H_), 1588, 1564, 1474, 1436 (s, ν_pyr_). MH^+^ = 291.22 *m*/*z*.

#### *N*,*N*-Bis­(2-pyridylmethyl)­ethanolamine (**DPEA**)

In a 250 mL round-bottom flask, 10 g (61 mmol) picolyl chloride hydrochloride was dissolved in 20 mL H_2_O and cooled to 0 °C in an ice bath. A solution of 5.0 g (120 mmol) NaOH in 20 mL H_2_O was added dropwise under stirring. Following this, a solution of ethanolamine (1.9 g, 31 mmol) in CH_2_Cl_2_ (40 mL) was added. The reaction mixture was then removed from the ice bath, capped, and allowed to stir vigorously for 9 days. The CH_2_Cl_2_ layer was then separated, washed twice with brine, and dried over anhydrous sodium sulfate. The solution was filtered and concentrated on a rotary evaporator producing 9.4 g of a red–brown oil that solidified upon cooling. The crude product was chromatographed on basic alumina (chromatographic grade, 80–200 mesh) eluting with 20:1 ethyl acetate/methanol (Rf = 0.49), producing 2.8 g (38%) of a pure, DPEA as a golden oil. ^1^H NMR (400 MHz, CDCl_3_) δ 8.55 (d, *J* = 4.9 Hz, 2H), 7.60 (t, *J* = 7.6 Hz, 2H), 7.32 (d, *J* = 7.8 Hz, 2H), 7.15 (t, *J* = 6.6 Hz, 2H), 3.93 (s, 4H), 3.70 (t, *J* = 4.8 Hz, 2H), 2.88 (t, *J* = 5.2 Hz, 2H). ^13^C NMR (101 MHz, CDCl_3_) δ 159.36, 149.06, 136.49, 123.08, 122.09, 60.11, 59.78, 56.82. IR (ATR, cm^–1^): 3293 (b, ν_O–H_), 3030–2820 (m, ν_C–H_), 1589, 1569, 1474, 1432 (s, ν_pyr_). MH^+^ = 244.17 *m*/*z*.

#### 2-Methoxy-*N*,*N*-bis­(pyridin-2-ylmethyl)­ethan-1-amine (**DPMEA**)

In a 250 mL round-bottom flask, 10 g (61 mmol) picolyl chloride hydrochloride was dissolved in 20 mL H_2_O and cooled to 0 °C in an ice bath. A solution of 5.0 g (120 mmol) NaOH in 20 mL H_2_O was added dropwise under stirring. Following this, a solution of methoxyethylamine (2.3 g, 31 mmol) in CH_2_Cl_2_ (40 mL) was added. The reaction mixture was then removed from the ice bath, capped, and allowed to stir vigorously for 8 days. The CH_2_Cl_2_ layer was then separated, washed twice with brine, and dried over anhydrous sodium sulfate. The solution was filtered and concentrated on a rotary evaporator producing 6.4 g of a red–brown oil that solidified upon cooling. The crude product was chromatographed on basic alumina (chromatographic grade, 80–200 mesh) eluting with 20:1 ethyl acetate/methanol (*R*_f_ = 0.52), producing 5.9 g (74%) of a pure, DPMEA as a golden oil. ^1^H NMR (400 MHz, CDCl_3_) δ 8.51 (d, *J* = 3.1 Hz, 2H), 7.64 (t, *J* = 7.2 Hz, 2H), 7.55 (d, *J* = 7.9 Hz, 2H), 7.13 (t, *J* = 6.0 Hz, 2H), 3.89 (s, 4H), 3.52 (t, *J* = 5.8 Hz, 2H), 3.28 (s, 3H), 2.80 (t, *J* = 5.8 Hz, 2H). ^13^C NMR (101 MHz, CDCl_3_) δ 159.79, 148.97, 136.43, 122.99, 121.94, 70.98, 60.86, 58.74, 53.58. IR (ATR, cm^–1^): 3200–2800 (m, ν_C–H_), 1589, 1569, 1473, 1432 (s, ν_pyr_). MH^+^ = 258.19 *m*/*z*.

#### 2,2′-((Pyridin-2-ylmethyl)­azanediyl)­bis­(ethan-1-ol) (**PDEA**)

In a 250 mL round-bottom flask, 5.0 g (30 mmol) picolyl chloride hydrochloride was dissolved in 10 mL H_2_O and cooled to 0 °C in an ice bath. A solution of 2.4 g (60 mmol) NaOH in 10 mL H_2_O was added dropwise under stirring. Following this, a solution of diethanolamine (4.1 g, 30 mmol) in CH_2_Cl_2_ (20 mL) was added. The reaction mixture was then removed from the ice bath, capped, and allowed to stir vigorously for 7 days. The CH_2_Cl_2_ layer was then separated, washed twice with brine, and dried over anhydrous sodium sulfate. The solution was filtered and concentrated on a rotary evaporator producing 5.5 g of a red–brown oil that solidified upon cooling. The crude product was chromatographed on basic alumina (chromatographic grade, 80–200 mesh) eluting with 10:1 dichloromethane/methanol (Rf = 0.50), producing 3.0 g (51%) of a pure, PDEA as a golden oil. ^1^H NMR (400 MHz, CDCl_3_) δ 8.56 (d, *J* = 4.9 Hz, 1H), 7.64 (t, *J* = 7.6 Hz, 1H), 7.25–7.16 (m, 2H), 3.90 (s, 2H), 3.59 (t, *J* = 4.8 Hz, 4H), 2.85 (t, *J* = 5.2 Hz, 4H).^13^C NMR (101 MHz, CDCl_3_) δ 159.42, 149.32, 137.12, 122.75, 122.52, 59.71, 59.28, 57.87. IR (ATR, cm^–1^): 3284 (b, ν_O–H_), 3030–2820 (m, ν_C–H_), 1595, 1571, 1478, 1432 (s, ν_pyr_). MH^+^ = 197.18 *m*/*z*.

#### 2-Methoxy-*N*-(2-methoxyethyl)-*N*-(pyridin-2-ylmethyl)­ethan-1-amine (**PDMEA**)

In a 250 mL round-bottom flask, 5.4 g (33 mmol) picolyl chloride hydrochloride was dissolved in 10 mL H_2_O and cooled to 0 °C in an ice bath. A solution of 2.5 g (63 mmol) NaOH in 10 mL H_2_O was added dropwise under stirring. Following this, a solution of bis­(2-methoxyethyl)­amine (2.3 g, 31 mmol) in CH_2_Cl_2_ (40 mL) was added. The reaction mixture was then removed from the ice bath, capped, and allowed to stir vigorously for 12 days. The CH_2_Cl_2_ layer was then separated, washed twice with brine, and dried over anhydrous sodium sulfate. The solution was filtered and concentrated on a rotary evaporator producing 3.3 g of a red–brown oil that solidified upon cooling. The crude product was chromatographed on basic alumina (chromatographic grade, 80–200 mesh) eluting with ethyl acetate (Rf = 0.70), producing 2.6 g (36%) of a pure, PDMEA as a golden oil. ^1^H NMR (400 MHz, CDCl_3_) δ 8.50 (d, *J* = 4.9 Hz, 1H), 7.63 (t, *J* = 8.1 Hz, 1H), 7.49 (d, *J* = 7.8 Hz, 1H), 7.12 (t, *J* = 6.0 Hz, 1H), 3.87 (s, 2H), 3.48 (t, *J* = 6.5 Hz, 4H), 3.29 (s, 6H), 2.80 (t, *J* = 6.0 Hz, 4H). ^13^C NMR (101 MHz, CDCl_3_) δ 159.79, 148.97, 136.43, 122.99, 121.94, 70.98, 60.86, 58.74, 53.58. IR (ATR, cm^–1^): 3200–2800 (m, ν_C–H_), 1587, 1568, 1472, 1431 (s, ν_pyr_). MH^+^ = 225.07 *m*/*z*.

### Complex Syntheses

#### [Mn­(TMPA)­(OAc)]­BPh_4_

In a 100 mL round-bottom flask, 0.41 g (1.4 × 10^–3^ moles) of TMPA was dissolved in 10 mL of methanol. To this, 0.35 g (1.4 mmol) of Mn­(OAc)_2_·4H_2_O was added, and the solution was brought to reflux for 30 min. A separate solution of 0.48 g (1.4 mmol) of NaBPh_4_ in 10 mL of methanol was added slowly to the warm reaction mixture. The solution was allowed to cool to room temperature as a solid, crystalline precipitate formed. The precipitate was collected by filtration and washed with cold methanol. The solid was allowed to air-dry producing 0.71 g (74%) of a tan crystalline product. Prior to washing the solid product, the reaction filtrate was collected, placed in a capped vial, and stored in the refrigerator to promote further crystallization. X-ray quality crystals were produced from this filtrate after several weeks that were used for diffraction. Elemental analysis: Calc (found) for C_44_H_40_BMnN_4_O_2_: C, 73.14 (72.72); H 5.58 (5.72); N 7.75 (7.72)%. IR (ATR, cm^–1^): 3200–2800 (m, ν_C–H_), 1529, 1416 (s, ν_C–O_), 704, 729 (s, ν_B–C_).

#### [Mn­(DPEA)­(OAc)]­BPh_4_

In a 100 mL round-bottom flask, 0.50 g (2.0 mmol) of DPEA was dissolved in 10 mL of methanol. To this, 0.50 g (2.0 mmol) of Mn­(OAc)_2_·4H_2_O was added, and the solution was brought to reflux for 30 min. A separate solution of 0.70 g (2.0 mmol) of NaBPh_4_ in 10 mL of methanol was added slowly to the warm reaction mixture. The solution was allowed to cool to room temperature as a solid, crystalline precipitate formed. The precipitate was collected by filtration and washed with cold methanol. The solid was allowed to air-dry producing 1.06 g (77%) of a tan crystalline product. Prior to washing the solid product, the reaction filtrate was collected, placed in a capped vial, and stored in the refrigerator to promote further crystallization. X-ray quality crystals were produced from this filtrate after several weeks that were used for diffraction. Elemental analysis: Calc (found) for C_40_H_40_BMnN_3_O_3_: C, 71.01 (70.75); H 5.82 (5.96); N 6.22 (6.21) %. IR (ATR, cm^–1^): 3437 (b, ν_O–H_), 3200–2800 (m, ν_C–H_), 1580, 1418 (s, ν_C–O_), 734, 707 (s, ν_B–C_).

#### [Mn­(DPMEA)­(OAc)]­BPh_4_

In a 100 mL round-bottom flask, 0.50 g (1.9 mmol) of DPMEA was dissolved in 10 mL of methanol. To this, 0.48 g (1.9 mmol) of Mn­(OAc)_2_·4H_2_O was added, and the solution was brought to reflux for 30 min. A separate solution of 0.66 g (1.9 mmol) of NaBPh_4_ in 10 mL of methanol was added slowly to the warm reaction mixture. The solution was cooled to room temperature, capped, and placed in a refrigerator overnight. The following day, a tan precipitate was collected by filtration and washed with cold methanol. The solid was allowed to air-dry producing 0.28 g (21%) of a tan crystalline product. Prior to washing the solid product, the reaction filtrate was collected, placed in a capped vial, and stored in the refrigerator to promote further crystallization. X-ray quality crystals were produced from this filtrate after several weeks that were used for diffraction. Elemental analysis: Calc (found) for C_40_H_39_BMnN_3_O_3_: C, 70.80 (71.30); H 6.81 (6.07); N 6.05 (6.16) %. IR (ATR, cm^–1^): 3200–2800 (m, ν_C–H_), 1605, 1578 (s, ν_C–O_), 758, 704 (s, ν_B–C_).

#### [Mn­(PDEA)­(OAc)]­BPh_4_

In a 100 mL round-bottom flask, 0.20 g (1.0 mmol) of PDEA was dissolved in 10 mL of methanol. To this, 0.25 g (1.0 mmol) of Mn­(OAc)_2_·4H_2_O was added, and the solution was brought to reflux for 30 min. A separate solution of 0.34 g (1.0 mmol) of NaBPh_4_ in 10 mL of methanol was added slowly to the warm reaction mixture. The solution was cooled to room temperature, reduced in volume to 10 mL by evaporation, capped, and placed in a refrigerator overnight. The following day, a tan precipitate was collected by filtration and washed with cold methanol. The solid was allowed to air-dry producing 0.228 g (43%) of a tan microcrystalline product. Prior to washing the solid product, the reaction filtrate was collected, placed in a capped vial, and stored in the refrigerator to promote further crystallization. X-ray quality crystals were produced from this filtrate after 1 week that were used for diffraction. Elemental analysis: Calc (found) for C_36_H_39_BMnN_2_O_4_: C, 68.69 (69.54); H 6.25 (6.54); N 4.45 (4.38) %. IR (ATR, cm^–1^): 3200–2800 (m, ν_C–H_), 1578, 1426 (s, ν_C–O_), 732, 704 (s, ν_B–C_).

#### [Mn­(PDMEA)­(OAc)]­BPh_4_

In a 100 mL round-bottom flask, 0.20 g (0.88 mmol) of PDMEA was dissolved in 10 mL of methanol. To this, 0.22 g (0.88 mmol) of Mn­(OAc)_2_·4H_2_O was added, and the solution was brought to reflux for 30 min. A separate solution of 0.30 g (0.88 mmol) of NaBPh_4_ in 10 mL of methanol was added slowly to the warm reaction mixture. The solution was cooled to room temperature, capped, and placed in a refrigerator overnight. The following day, a tan precipitate was collected by filtration and washed with cold methanol. The solid was allowed to air-dry producing 0.53 g (90%) of a tan crystalline product. Prior to washing the solid product, the reaction filtrate was collected, placed in a capped vial, and stored in the refrigerator to promote further crystallization. X-ray quality crystals were produced from this filtrate after several weeks that were used for diffraction. Elemental analysis: Calc (found) for C_38_H_43_BMnN_2_O_4_: C, 69.42 (68.26); H 6.59 (6.74); N 4.26 (4.10) %. IR (ATR, cm^–1^): 3200–2800 (m, ν_C–H_), 1548, 1426 (s, ν_C–O_), 731, 706 (s, ν_B–C_).

#### [Mn­(TEA)­(OAc)]­BPh_4_

In a 100 mL round-bottom flask, 0.22 g (1.5 mmol) of TEA was dissolved in 5 mL of methanol. The solution capped with a septum and a vent needle was added. Nitrogen was bubbled through the solution for 15 min. To this solution, 0.36 g (1.5 mmol) of Mn­(OAc)_2_·4H_2_O was added, and the solution was stirred for 15 min while nitrogen bubbling continued. A separate solution of 0.51 g (1.5 mmol) of NaBPh_4_ in 5 mL of methanol was added and the solution was stirred an additional 10 min under nitrogen bubbling. The solution was then placed in the refrigerator overnight. The following day, a tan precipitate was collected by filtration and washed with cold methanol. The solid was allowed to air-dry producing 0.49 g (49%) of a tan crystalline product. X-ray quality crystals were produced from this filtrate after several weeks that were used for diffraction. IR (ATR, cm^–1^): 3600–3100 (b, ν_O–H_), 3000–2800 (m, ν_C–H_), 1578, 1423 (s, ν_C–O_), 735, 707 (s, ν_B–C_).

### X-ray Structural Data Collection

Suitable single crystals were selected, attached to a nylon loop, and mounted on either a Rigaku XtaLAB Synergy-S Dualflex diffractometer, equipped with a HyPix 6000-HE HPC detector and a Cryostream 800 low-temperature cryostat (compounds [Mn­(PDEA)­(OAc)­(MeOH)]­BPh_4_ and [Mn­(TEA)­(OAc)­(MeOH)]­BPh_4_·2 MeOH), or a Rigaku Gemini Eos Multiscan (compounds [Mn­(DPEA)­(OAc)­(MeOH)]­BPh_4_·MeOH, [Mn­(DPMEA)­(OAc)­(MeOH)]­BPh_4_·2 MeOH, and [Mn­(PDMEA)­(OAc)­(MeOH)]­BPh_4_). Crystals were kept at 100 K during data collection unless otherwise noted and used either Mo or Cu Kα radiation. With Olex2 as a GUI,^23^ structures were solved with the SHELXT structure solution program by intrinsic phasing^24^ and refined with the SHELXL refinement package using least-squares minimization.^25^ Crystal data and refinement details are provided in the Supporting Information (Tables S1–S33).

### Potentiometric Titrations

Titrations were performed using a Thermo Scientific OrionStar T910 automatic titrator equipped with a Ross Orion 8102BNUWP pH electrode. Electrode calibration was performed prior to each experiment using the GLEE method.^20^ The electrode standard potential and slope were used to convert millivolt readings from each titration into pH values. An air-sealed, jacketed titration vessel was employed, equipped with ports for titrant addition, nitrogen gas, and the pH probe. Temperature was maintained at 25.0 ± 0.1 °C with a temperature-controlled, circulating water bath. Air was excluded from the titration by purging both the titrant bottle and titration vessel with a stream of nitrogen gas, passed through a sparging stone, while the vessel was vented with a syringe needle.

Reagent stock solutions were prepared in DI H_2_O (18 MΩ cm^–1^). Ligand stock solutions ranging in concentration from 0.0200 to 0.200 M were solubilized by the addition of 10% CH_3_CN. The concentration of MnCl_2_(aq) stock (0.1043 M) was determined by titration with commercially standardized ethylenediaminetetraacetic acid (EDTA) using Eriochrome black T indicator. A 0.1008 M stock solution of HCl was standardized by titration in triplicate against oven-dried (12 h) tris­(hydroxymethyl)­aminomethane (Tris), using methyl red as an indicator. Carbonate-free stock solutions of NaOH were prepared using DI H_2_O that had been boiled for 15 min, cooled under a stream of nitrogen, and stored in a sealed bottle. The NaOH stock concentrations of either 0.0100 or 0.0500 M were determined by titrating them in triplicate against the standardized HCl solution.

The quantities of ligand, HCl, and MnCl_2_, and concentrations of KCl and NaOH titrant used for each experiment are given in the Supporting Information (Table S34). These quantities were chosen to produce optimum titration curves for the analysis of Mn^2+^ complex of each ligand. For a typical experiment, the appropriate quantities of ligand and HCl were added to the titration vessel along with enough DI H_2_O to give 40 mL of solution. A 0.3 g quantity of KCl was also added to give the solution an ionic strength of 0.1 M. The acidified ligand solution was then titrated in triplicate with NaOH of the concentration given in Table S34. Hyperquad 2013 software^21^ was used to analyze the data sets which were fit to a model where the ligands bind either one or two protons, producing protonation constants. The titration above was repeated in triplicate in the presence of MnCl_2_, generating a modified curve that was analyzed using Hyperquad 2013, holding the protonation constant(s) of the ligand constant and fitting for the stability constant of the [MnL]^2+^ complex. Once the protonation constants and [MnL]^2+^ stability constant were determined, HySS software^26^ was used to produce speciation plots under the conditions of the titration, and to determine the concentrations of Mn^2+^ and L used in cyclic voltammetry and SOD activity assays described herein, in order to ensure near-complete (TMPA, DPEA, or DPMEA) or substantial (PDEA or PDMEA) [MnL]^2+^ complex formation under the conditions of the experiment.

**Table 1:** Selected Bond Lengths and Angles for Compounds **1–6**

cmpd	selected bond lengths [Å]	selected bond angles [deg]	
**1** ^22^	Mn1–O1 2.1941(12)	O1–Mn1–O2 81.52(4)	O1–Mn1–O3 101.03(5)
	Mn1–O2 2.5009(12)	O1–Mn1–N1 88.33(5)	O1–Mn1–N2 92.28(5)
	Mn1–O3 2.2004(13)	O1–Mn1–N3 88.03(4)	O1–Mn1–N4 166.95(5)
	Mn1–N1 2.2769(15)	N4–Mn1–N1 91.07(5)	N4–Mn1–N2 75.20(4)
	Mn1–N2 2.4092(13)	N4–Mn1–N3 84.61(4)	N4–Mn1–O2 111.30(4)
	Mn1–N3 2.3022(13)	N4–Mn1–O3 88.91(5)	O3–Mn1–O2 54.74(4)
	Mn1–N4 2.2496(13)	N1–Mn1–O2 81.88(5)	N1–Mn1–N2 71.41(5)
		N3–Mn1–N2 71.94(5)	O3–Mn1–N3 83.91(5)
**2**	Mn1–O1 2.3185(10)	O2–Mn1–O1 78.01(4)	O2–Mn1–O3 89.66(4)
	Mn1–O2 2.1866(11)	O2–Mn1–O4 90.42(5)	O2–Mn1–N2 103.23(5)
	Mn1–O3 2.2346(10)	O2–Mn1–N3 82.25(4)	O2–Mn1–N1 173.93(5)
	Mn1–O4 2.3670(11)	N1–Mn1–O1 96.13(4)	N1–Mn1–O3 90.77(4)
	Mn1–N1 2.2768(12)	N1–Mn1–O4 94.86(4)	N1–Mn1–N2 73.36(4)
	Mn1–N2 2.3528(12)	N1–Mn1–N3 101.14(4)	O3–Mn1–O1 79.13(4)
	Mn1–N3 2.3016(12)	O3–Mn1–O4 56.47(4)	N3–Mn1–O4 85.23(4)
		N2–Mn1–N3 71.82(4)	N2–Mn1–O1 74.11(4)
**3**	Mn1–O1 2.3064(14)	O3–Mn1–O1 88.35(6)	O3–Mn1–O2 97.82(6)
	Mn1–O2 2.3296(14)	O3–Mn1–O4 76.44(6)	O3–Mn1–N2 84.54(6)
	Mn1–O3 2.1800(14)	O3–Mn1–N3 93.29(6)	O3–Mn1–N1 164.33(6)
	Mn1–O4 2.3556(15)	N1–Mn1–O1 106.97(6)	N1–Mn1–O2 88.41(6)
	Mn1–N1 2.2564(16)	N1–Mn1–O4 90.45(6)	N1–Mn1–N2 100.25(6)
	Mn1–N2 2.3049(16)	N1–Mn1–N3 74.42(6)	O1–Mn1–O2 56.02(6)
	Mn1–N3 2.3578(16)	O2–Mn1–O4 81.03(6)	O1–Mn1–N2 83.04(6)
		N2–Mn1–N3 71.49(6)	N3–Mn1–O4 73.05(6)
**4**	Mn1–O1 2.2565(19)	O1–Mn1–O2 113.08(8)	O1–Mn1–O3 85.46(7)
	Mn1–O2 2.2506(18)	O1–Mn1–O4 71.82(6)	O1–Mn1–N1 73.30(7)
	Mn1–O3 2.1767(17)	O1–Mn1–N2 71.15(6)	O1–Mn1–O5 160.94(8)
	Mn1–O4 2.5072(18)	O5–Mn1–O2 83.90(9)	O5–Mn1–O3 86.61(7)
	Mn1–O5 2.254(2)	O5–Mn1–O2 83.90(9)	O5–Mn1–O3 86.61(7)
	Mn1–N1 2.411(2)	O5–Mn1–O2 83.90(9)	O5–Mn1–O3 86.61(7)
	Mn1–N2 2.231(2)	O5–Mn1–N2 79.90(8)	O3–Mn1–O4 55.39(6)
		O2–Mn1–O3 88.03(7)	O3–Mn1–O4 55.39(6)
		O4–Mn1–N2 91.61(7)	N1–Mn1–N2 72.40(7)
**5**	Mn1–O1 2.1551(10)	O1–Mn1–O2 90.23(4)	O1–Mn1–O3 102.57(4)
	Mn1–O2 2.3445(10)	O1–Mn1–O4 86.06(4)	O1–Mn1–O5 81.56(4)
	Mn1–O3 2.2062(11)	O1–Mn1–N2 91.81(4)	O1–Mn1–N1 160.35(5)
	Mn1–O4 2.3361(10)	N1–Mn1–O2 94.25(4)	N1–Mn1–O3 95.91(5)
	Mn1–O5 2.3842(10)	N1–Mn1–O4 103.55(4)	N1–Mn1–O5 80.48(4)
	Mn1–N1 2.2471(13)	N1–Mn1–N2 75.45(5)	O2–Mn1–O3 57.17(4)
	Mn1–N2 2.3225(12)	O3–Mn1–O4 81.05(4)	O4–Mn1–N2 71.77(4)
		N2–Mn1–O5 73.41(4)	O2–Mn1–O5 79.41(3)
**6**	Mn1–O1 2.2123(15)	O2–Mn1–O1 93.56(6)	O2–Mn1–O3 90.50(6)
	Mn1–O2 2.2401(13)	O2–Mn1–O5 79.16(5)	O2–Mn1–O6 97.95(5)
	Mn1–O3 2.2891(15)	O2–Mn1–N1 76.83(5)	O2–Mn1–O4 176.07(6)
	Mn1–O4 2.1821(14)	O4–Mn1–O1 85.79(6)	O4–Mn1–O3 87.84(6)
	Mn1–O5 2.4410(14)	O4–Mn1–O5 104.57(6)	O4–Mn1–O6 85.31(5)
	Mn1–O6 2.1998(13)	O4–Mn1–N1 99.27(6)	O1–Mn1–N1 74.65(6)
	Mn1–N1 2.3192(15)	N1–Mn1–O3 72.16(6)	O3–Mn1–O6 80.84(5)
		O5–Mn1–O6 55.66(5)	O5–Mn1–O1 82.49(5)

### Cyclic Voltammetry

Cyclic voltammograms were recorded in 50 mM collidine buffer, pH 7.5, using an eDAQ 466 Integrated Potentiostat System with EChem software. Mn^2+^ complexes were formed in situ in the presence of excess ligand to ensure a high percentage of complex formation (see Table S35 in Supporting Information) according to our stability studies vide infra. A glassy carbon working electrode, Ag/AgCl reference electrode, and Pt/Ti auxiliary electrode were employed. Prior to recording CVs, solutions were bubbled with nitrogen gas for 15 min to eliminate oxygen. CVs were then recorded at a scan rate of 100 mV/s.

### McCord-Fridovich Assays

The McCord–Fridovich assay^27^ was used to measure the SOD activity of the Mn^2+^ complexes of our ligands formed in situ. This assay utilizes the reaction of xanthine oxidase with xanthine to produce superoxide ion which, in turn, reduces ferricytochrome C. The reduction of ferricytochrome C is monitored at 550 nm in the absence and presence of SOD mimics to determine the concentration at which the complexes inhibit ferricytochrome C reduction by 50%, their IC_50_ value. Assays were run in 50 mM HEPES buffer, pH 7.5, containing an excess of ligand to ensure as high a concentration of Mn^2+^ complex formation possible at the concentrations of MnCl_2_ employed (see Table S36 in Supporting Information). Stock solutions of cytochrome C, xanthine, xanthine oxidase, catalase, and MnCl_2_ were also prepared with this buffer. The temperature of the reactions was held constant at 25 °C using a Peltier attachment to the spectrophotometer. For a typical reaction, a solution was prepared in a cuvette containing 50 μM cytochrome c, 50 μM xanthine, 30 μg/mL catalase, and enough buffer to provide a total of 3 mL of solution. Approximately 100 μg/mL xanthine oxidase was added to initiate the reaction. This quantity was adjusted to give an initial velocity of 0.025–0.030 ΔAbs_550nm_/min. The reaction was monitored at 550 nm. After 2.5 min, a quantity of MnCl_2_ was added and the absorbance was monitored for another 2.5 min. Reactions were run in triplicate for each different concentration of Mn^2+^ examined, and percent inhibition was calculated as $\%I=\left(\frac{V_{0}-V_{f}}{V_{0}}\right)\times 100\%$ , where *V*_0_ and *V*_f_ are the initial and final velocities respectively. Plots of %*I* vs [Mn^2+^] were used to determine the IC_50_ for each complex. To verify the fidelity of the McCord-Fridovich assay, the production of urate by the xanthine/xanthine oxidase reaction at 290 nm in the absence and presence of each complex, and no inhibition at a concentration higher than the IC_50_ was observed. Likewise, ferricytochrome C was found to be stable in the presence of our complexes.

## Results and Discussion

### Ligand Syntheses

The tetradentate ligands examined in this study, aside from TEA (obtained commercially), were synthesized in good yield by the reaction of 2-chloromethylpyridine with 2-(aminomethyl)­amine, hydroxyethylamine or methoxyethylamine starting materials. The tripodal structure of these compounds permitted the ability to make sequential substitutions of oxygen moieties (−OH or –OCH_3_) for pyridyl groups, resulting in a series of compounds that differ in their nitrogen and oxygen content without altering geometric or steric characteristics to any great extent (see Figure 1). Variations with either hydroxide or methoxide groups also permitted examination of these groups on the influence of Mn^2+^ complexation, and the catalytic activity of the resulting [MnL]^2+^ complexes.

### Complex Syntheses

Mn^2+^ complexes of each of the ligands were formed by the reaction of Mn­(OAc)_2_ with the ligand followed by anion exchange with one equivalent of tetraphenylborate, promoting crystallization of a compound in which one acetate ion serves as an additional ligand. We originally set out to make Mn^3+^ complexes of TMPA, DPEA, and DPMEA using Mn­(OAc)_3_, but found that these reactions produced Mn^2+^ compounds in approximately 50% yield. Although this might suggest a disproportionation, the process is accompanied by a color change of the reaction solution from dark brown to straw color as the reaction proceeds, and no evidence of a colored Mn^4+^ byproduct is observed. Reduction of Mn^3+^ involving the tripodal ligands and/or acetate is therefore more likely. Using Mn­(OAc)_2_ as a starting material results in yields that are typically higher. However, for ligands with higher oxygen content, PDEA, PDMEA, or TEA, reactions with Mn­(OAc)_2_ darken with extended reaction times (>30 min), indicating that they promote oxidation of pale-colored Mn^2+^ to a higher oxidation state of Mn. These observations suggest that this set of tripodal ligands occupies an interesting range of influence over their Mn­(II/III) complexes, with higher N-donor content stabilizing Mn^2+^ and higher O-donor content promoting oxidation of Mn^2+^.

### X-ray Crystallography

Crystals suitable for X-ray diffraction of the complexes were obtained by cooling methanolic solutions of the compounds at 4 °C (see Tables S1–S33 in Supporting Information for crystallographic parameters and data). Note that the structure of [Mn­(TMPA)­(OAc)­(MeOH)]­BPh_4_ has been described previously.^22^ Structural analysis of each compound reveals a mononuclear species where the cationic portion is a hepta–coordinate complex in which the geometry is best described as distorted, pentagonal bipyramidal (see Figure 2). Selected bond lengths and angles are given in Table 1. Although a coordination number of seven is high for first row transition metals, it is not uncommon for Mn^2+^ ions with N-donor ligands.^28,29^ In each case, the tripodal ligand is tetra-coordinate with the central amine nitrogen and the coordinating atoms of two other arms (pyridyl nitrogen and/or oxygen from hydroxy or methoxy) sitting in the pentagonal plane, and the coordinating atom of a third arm occupying an axial position. The remaining two positions of the pentagonal plane are occupied by the asymmetric, bidentate coordination of an acetate ion, while the final axial position is filled by oxygen of a methanol ligand. For each complex, distortion away from a pentagonal pyramidal geometry results from the constraints of the tripodal ligand and bidentate coordination of the acetate ligand. For example, the bond angle in the metallacycle formed between the axially located atom of the tripodal ligand, manganese, and the equatorial amine nitrogen ranges from 73.30(7)° (**5**) to 76.83(5)° (**6**), significantly less than 90°. Likewise, the bond angle formed by the oxygens and manganese in the chelate ring that result from bidentate coordination of the acetate ligands ranges from 54.74(4)° (**1**) to 57.17(4)° (**4**), significantly reduced from the ideal 72° bond angle within the pentagonal plane. The bond angle formed by the axial ligands and manganese also deviates significantly from 180°, ranging from 160.35(5)° (**4**) to 176.07(6)° (**6**). Using the complex numbering scheme identified in Figure 2, axial distortion follows (from smallest to largest) (**6**) < (**2**) < (**1**) < (**3**) < (**5**) ∼ (**4**); there appears to be no correlation between measured distortions and reduction potentials or SOD activity. The average Mn–N bond lengths for pyridyl nitrogen atoms is 2.27(3) Å, falling in a typical range for Mn^2+^ bond lengths with N- or O-donor ligands, 2.2–2.3 Å.^30–33^ The same is true for the Mn–O (hydroxyl) bonds lengths in compounds (**2**), (**4**), and (**6**), averaging 2.26(4) Å. However, the Mn–O (methoxy) bond lengths in (**3**) and (**5**) are somewhat longer averaging 2.36(2) Å, indicating that the methyl groups impose a steric or electronic effect on bonding. Likewise, the longer Mn–N (central amine) bond distances, which average 2.36(4) Å for the complexes, have been observed previously in complexes with other tripodal, tetradentate ligands.^28–30,33^ Mn–N (central amine) bond distances range from 2.3192(16) Å (**6**) to 2.411(3) Å (**4**) following (**6**) ∼ (**5**) < (**2**) < (**1**) < (**3**) < (**4**) (from shortest to longest); there appears to be no correlation between measured bond distances and reduction potentials or SOD activity (vide infra). Structural overlays of (**1**)/(**2**), (**1**)/(**3**), and (**2**)/(**3**) bis­(pyridyl) complexes, (**4**)/(**6**) bis­(CH_2_CH_2_OH) complexes, and of (**5**)/(**6**), through Mn and N (central amine) matching, showed very similar atomic positions (nearly superimposed). Mn, N (central amine), and N (pyridyl) matching of (**4**)/(**5**) overlaid nicely, however showed differences in acetate ligand orientation and methanol coordination (Figure 2).

**Figure 2:**
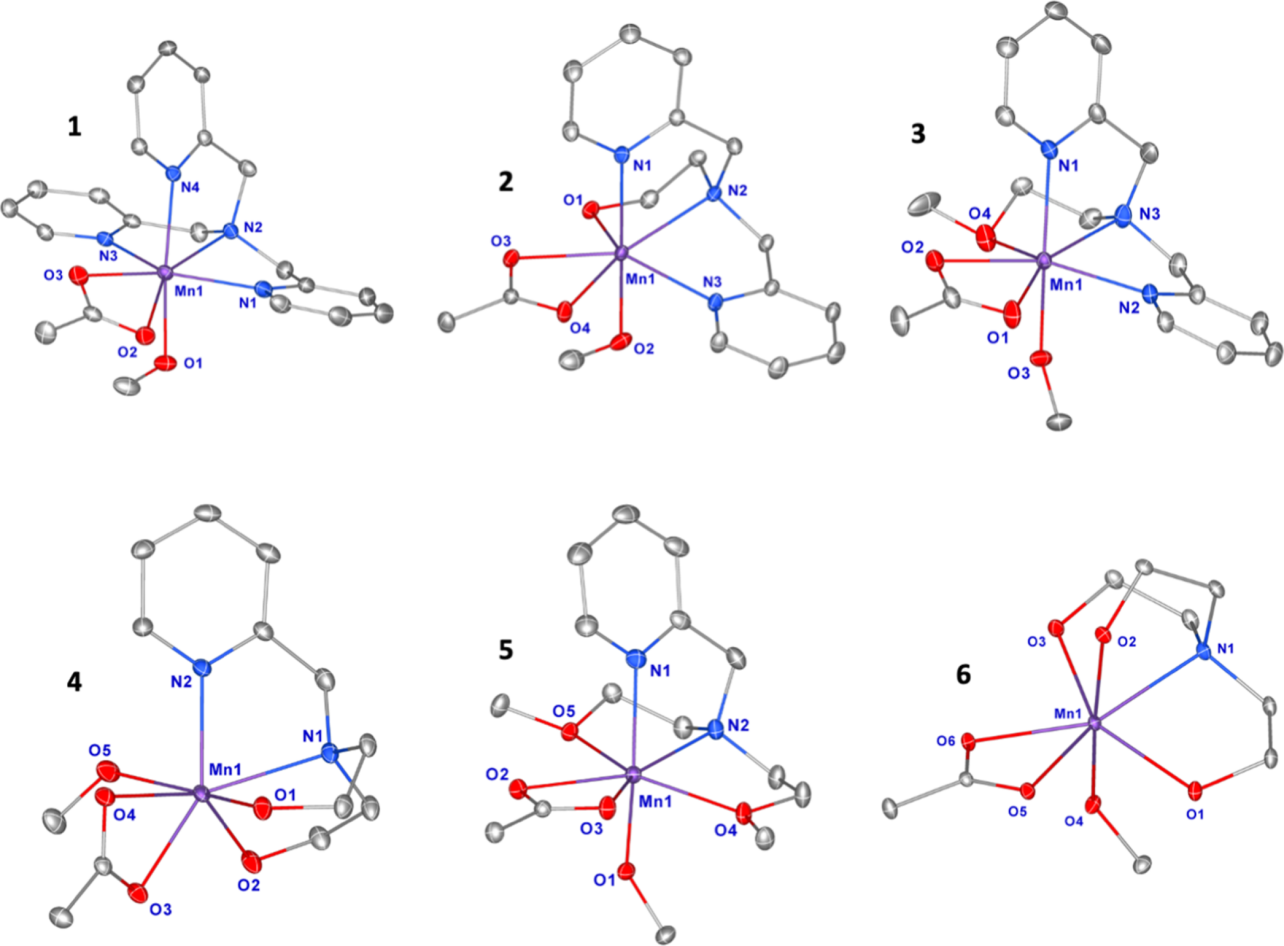
Ellipsoid structures of the cationic portion of [Mn­(TMPA)­(OAc)­(MeOH)]­BPh_4_ (**1**),^22^ [Mn­(DPEA)­(OAc)­(MeOH)]­BPh_4_·MeOH (**2**), [Mn­(DPMEA)­(OAc)­(MeOH)]­BPh_4_·2 MeOH (**3**), [Mn­(PDEA)­(OAc)­(MeOH)]­BPh_4_ (**4**), [Mn­(PDMEA)­(OAc)­(MeOH)]­BPh_4_ (**5**) and [Mn­(TEA)­(OAc)­(MeOH)]­BPh_4_·2 MeOH (**6**) shown with (40% ellipsoids). Hydrogen atoms, the tetraphenylborate anion, and noncoordinating solvent molecules have been omitted for clarity.

### Aqueous Stability of the Complexes

Prior to conducting reactivity studies and electrochemical measurements, it was necessary to determine the aqueous stability of in situ complex formation between Mn^2+^ and each of the tripodal ligands. This was examined through potentiometric titrations of the acidified ligands in the absence and presence of MnCl_2_, using NaOH as the titrant. Examples of these titrations are shown in Figure 3 along with speciation plots that result from their analyses. Protonation constants (β_LH1_ and β_LH2_) for the ligands and stability constants for [MnL]^2+^ complex formation (β_MnL_) were determined by a nonlinear, least-squares fitting of the potentiometric data to speciation models in which the ligands bind one (PDEA, PDMEA, or TEA) or two (TMPA, DPEA, or DPMEA) protons and form mononuclear complexes with Mn^2+^. The crystal structures vide infra lead us to believe that these complexes, formed in the absence of acetate and methanol, must also include aqua ligands to complete the Mn^2+^ coordination sphere. This is corroborated by an event that occurs typically between pH 8–9 that models best as deprotonation of a coordinated water molecule, providing p*K*_a_ values for the complexes. Between pH 9 and 10, precipitation occurs, presumably due to aggregation of hydroxyl-complex species, and we have excluded points at and beyond this event in our analyses. Results of the potentiometric experiments are summarized in Table 2. Fitted titration plots and speciation plots for the [MnL]^2+^ complexes can be found in the Supporting Information (Figures S22–S28).

**Figure 3:**
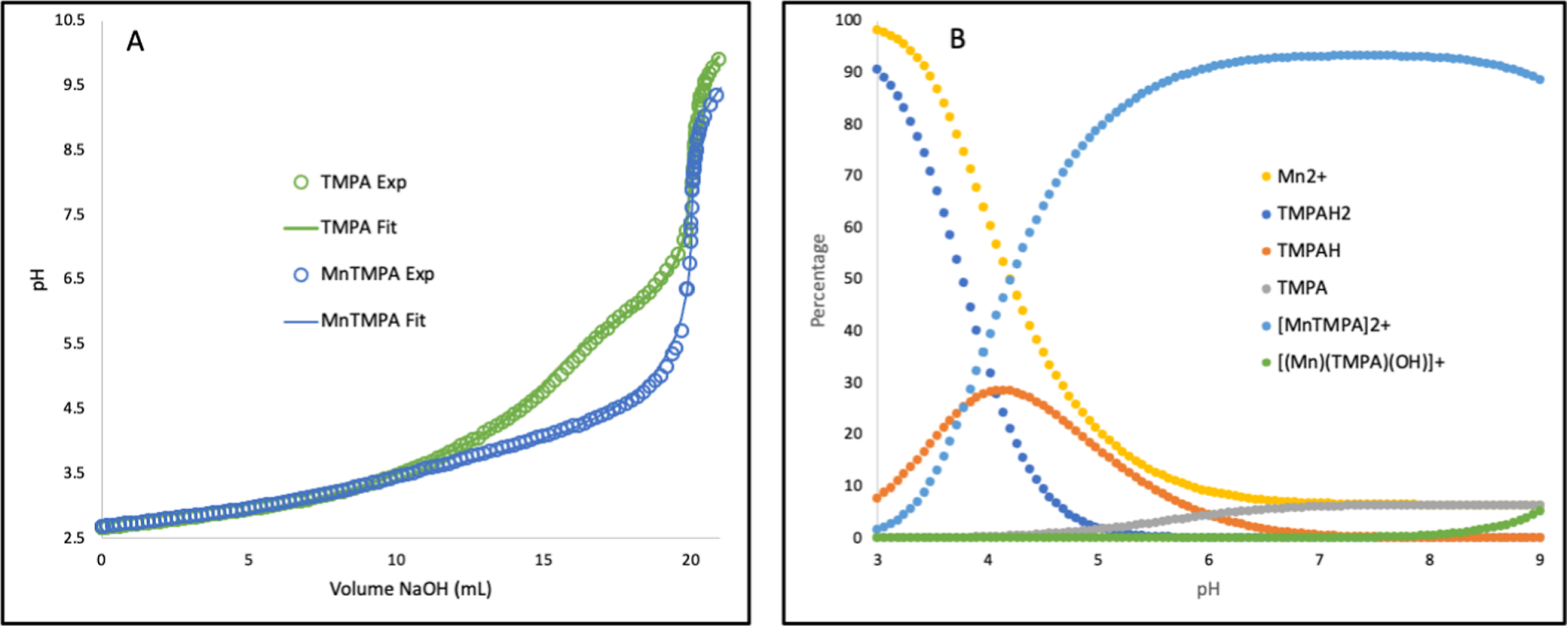
(A) Potentiometric titrations of 0.0500 mmol tris­(pyridin-2-ylmethyl)­amine (TMPA) and 0.100 mmol HCl in DI water containing 0.100 M KCl by 0.0100 M NaOH (green circles), and 0.0500 mmol TMPA, 0.100 mmol HCl, and 0.0500 mmol MnCl_2_ in DI water containing 0.100 M KCl by 0.0100 M NaOH (blue circles). The protonation constants for TMPA and stability constant for [MnTMPA­(H_2_O)_*x*_]^2+^ complex formation were determined by a nonlinear, least-squares fitting (Hyperquad 2013 ^21^) of the potentiometric data (solid lines) to speciation models in which the TMPA binds two protons and forms a mononuclear complex with Mn^2+^. The *K*_a_ value of a coordinated water molecule is also accounted for in this model. (B) Speciation plot (HYSS^26^) produced from the protonation constants for the TMPA ligand, stability constant (β_MnL_) for the [MnTMPA­(H_2_O)_*x*_]^2+^ complex, and *K*_a_ value of a coordinated water molecule, where [TMPA] = [Mn^2+^] = 1.25 mM.

**Table 2:** Protonation Constants of the Tripodal Ligands Examined in This Study, Stability Constants of Their Mn^2+^ Complexes, and Acid Dissociation Constant of an Associated Aqua Ligand, MnL­(H_2_O) (*I* = 0.10 M KCl, *t* = 25 °C)

	TMPA	DPEA	DPMEA	PDEA	PDMEA	TEA
Log β_LH1_	6.01(1)	6.10(1)	6.19(1)	6.78(1)	6.90(2)	7.783(4)
Log β_LH2_	10.08(1)	9.40(3)	9.81(1)	N/A	N/A	N/A
Log β_MnL_	5.24(2)	4.12(1)	3.71(2)	2.38(2)	2.32(2)	N/A
p*K*_a_MnL(H_2_O)	4.98(3)	5.63(1)	6.07(2)	7.00(2)	6.6(1)	N/A

As shown in Table 2, the stability constants for Mn^2+^ complexation by the ligands vary considerably, over 5 orders of magnitude, and reveal weak to moderate stability of the complexes in aqueous solution. This wide variation permits inspection of the N/O content of the ligands on Mn^2+^ complex stability. Indeed, the log of the stability constants (Log β_MnL_) correlate linearly to the number of coordinating oxygen atoms supplied by each ligand, whereby higher oxygen content results in lower stability, as shown in Figure 4. Note that [Mn­(TEA)]^2+^ is not included in Figure 4 since we were unable to measure any formation of this complex under the conditions of the titration. This correlation may seem counterintuitive, given that Mn^2+^ is considered a hard metal ion with a preference for oxygen-donor ligands.^34^ Nonetheless, within this series of ligands, higher nitrogen content leads to greater Mn^2+^ complex stability. This is consistent with our observation that reacting Mn­(OAc)_3_ with TMPA, DPEA, or DPMEA results in the isolation of Mn^2+^ complexes.

**Figure 4:**
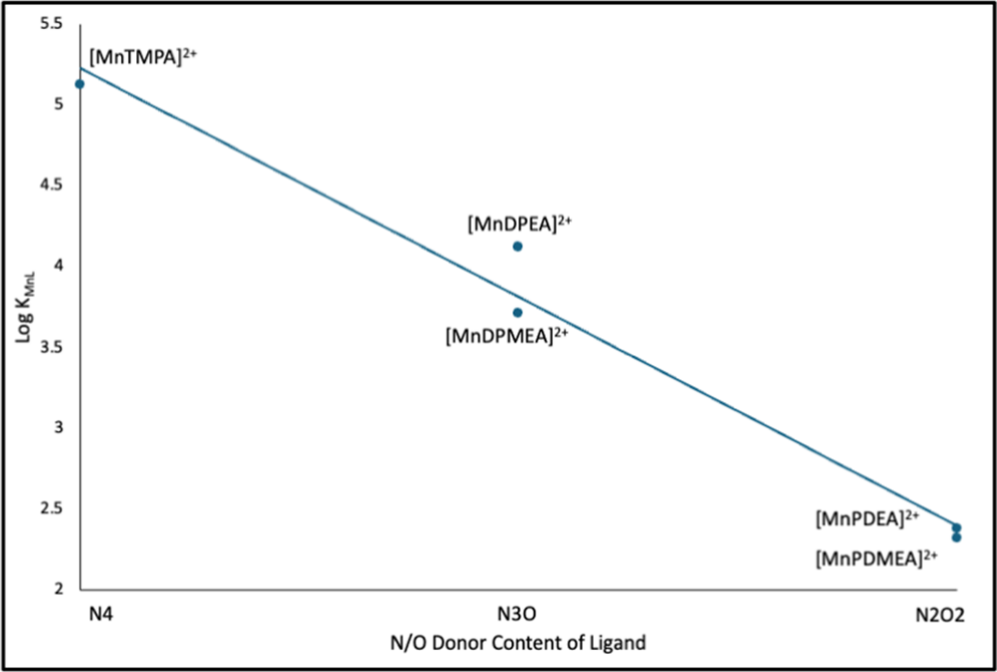
Plot of the log of the stability constant (log β_MnL_) for the formation of the Mn^2+^ complex with each ligand in aqueous solution as a function of the N/O donor content of the ligand.

### Cyclic Voltammetry

Cyclic voltammograms of the Mn^2+^ complexes of TMPA, DPEA, DPMEA, PDEA, and PDMEA, prepared in situ in buffered aqueous solution (pH 7.5), were recorded vs Ag/AgCl to determine the reduction potential (*E*_1/2_) for the Mn^III^/Mn^II^ couple. A cyclic voltammogram of MnCl_2_ in the buffered solution was also recorded for the purpose of comparison. Collidine buffer was employed for analysis since it tends to permit visualization of electrochemical waves for Mn^2+^ complexes with pyridine-containing ligands, where other solvents/buffers can lead to ill-defined or absent features for these species.^35^ Ligand and MnCl_2_ concentrations were chosen to ensure near complete (N_4_ and N_3_O), or substantial (N_2_O_2_) complex formation according to our stability studies. For [MnTMPA]^2+^, an equimolar amount of TMPA and MnCl_2_ were used (0.001 M), and for all other complexes, ligand concentration (0.01 M) was 10-fold the concentration of MnCl_2_ (0.001 M). In each case, irreversible waves were recorded where the difference in oxidation and reduction peak positions (Δ*E*_p_) is greater than 59 mV. Redox potential values (*E*_1/2_), determined from the average of the positions of the oxidation and reduction peaks, are given in Table 3 along with Δ*E*_p_ values. The distinct positions of these peaks, relative to those of MnCl_2_, further corroborate [MnL]^2+^ aqueous complex formation of the N_4_, N_3_O, and N_2_O_2_ ligands employed. Voltammograms can be found in Supporting Information (Figures S29–S34 **).**

**Table 3:** Cyclic Voltammetry Data for Mn^2+^ Complexes Formed in situ and MnCl_2_ in Collidine Buffer, pH 7.5^a^

Mn Species	N/O	*E*_oxid_ (V)	*E*_red_ (V)	Δ*E*_p_	*E*_1/2_ (V) vs Ag/AgCl	*E*_1/2_ (V) vs SHE*
[MnTMPA]^2+^	N4	0.786	0.282	0.504	0.534	0.731
[MnDPEA]^2+^	N3O	0.658	0.288	0.370	0.473	0.670
[MnDPMEA]^2+^	N3O	0.620	0.278	0.342	0.449	0.646
[MnPDEA]^2+^	N2O2	0.462	0.230	0.232	0.346	0.543
[MnPDMEA]^2+^	N2O2	0.474	0.228	0.246	0.351	0.548
MnCl_2_	N/A	0.415	0.130	0.285	0.273	0.470
Escherichia coli MnSOD^8^	N/A	-	-	-	-	0.40
Bascillus stearothermophilus MnSOD^8^	N/A	-	-	-	-	0.28
Human MnSOD^12^	N/A	-	-	-	-	0.29

a Data was obtained at a scan rate of 100 mV/s against an Ag/AgCl working electrode. Redox potentials (*E*_1/2_) converted to V vs SHE using *E*_1/2_ SHE = *E*_1/2_ Ag/AgCl + 0.197 V are given in the table for the purpose of comparison to those of MnSOD enzymes.

The *E*_1/2_ values for the complexes correlate well with the oxygen content of the ligands, where increasing oxygen content results in a lower *E*_1/2_ value (see Figure 5). This has been observed previously in Mn^2+^ complexes with tripodal ligands containing methylimidazole and carboxylate groups and can be ascribed to the stabilization of Mn^3+^ by the hard oxygen donors.^35^ The plot also suggests the opposite of course, that higher nitrogen content of the ligands stabilizes Mn^2+^, as demonstrated by the potentiometry studies herein. The Δ*E*_p_ values also correlate with N/O donor content of the ligands where higher oxygen content results in lower Δ*E*_p_ values. Irreversibility of the waves likely results from the instability of the Mn^3+^ species formed by oxidation. The smaller Δ*E*_p_ values observed for the oxygen-rich ligands further suggests that they help stabilize Mn^3+^, moving the process in the direction of reversibility.

**Figure 5:**
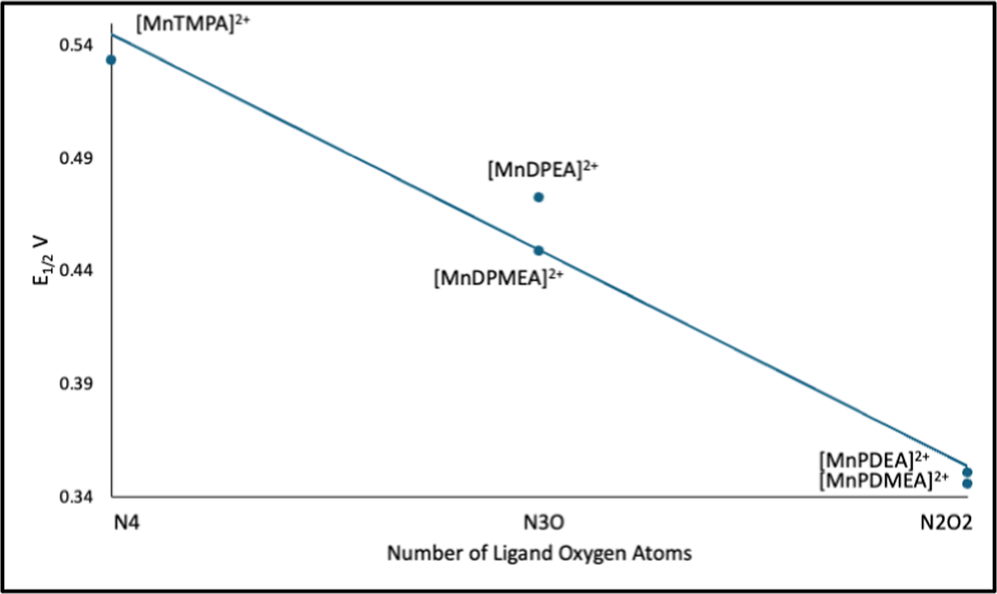
Plot of the reduction potential, *E*_1/2_ vs Ag/AgCl for Mn^2+^ complexes formed in situ in collidine buffer, pH 7.5 vs the N/O donor content for each ligand.

The *E*_1/2_ values measured for each of the complexes fall in a range between the reduction potentials of molecular oxygen (−0.16 V) and superoxide ion (0.89 V), a critical characteristic for catalysts of superoxide disproportionation (see Figure 6).^8,9^ Note that reduction potentials for the complexes have been converted to values relative to SHE in Figure 6 (*E*_1/2_ SHE = *E*_1/2_ Ag/AgCl + 0.197 V) for the purpose of comparing them to SOD enzyme values. The *E*_1/2_ values of SOD enzymes are centrally positioned between the limits of oxygen and superoxide ion reduction, a factor undoubtedly enhances their catalytic efficiency.^8^ The reduction potentials for each of the complexes is greater than those of known SODs,^8^ implying that they would more favorably oxidize O_2_^•–^ than reduce it.

**Figure 6:**
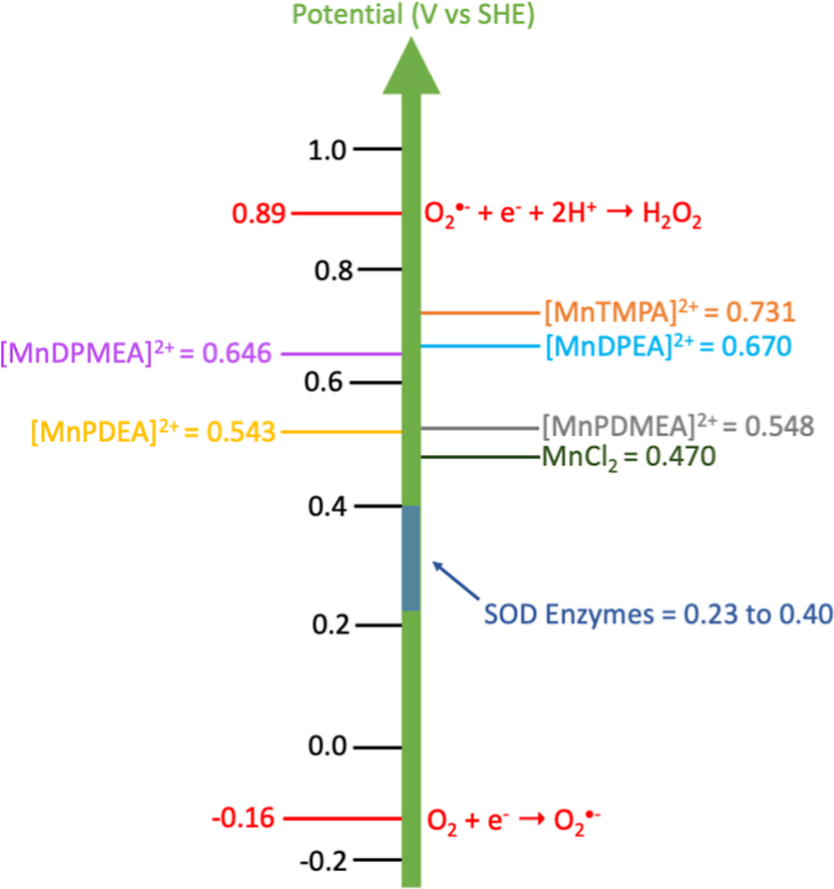
Diagram showing the redox potentials, *E*_1/2_ vs SHE for the complexes studied herein, along with SOD enzymes^8^ and MnCl_2_. These potentials are within the range of −0.16 and 0.89 V where reduction of O_2_ and O_2_^•–^ occur respectively, a requirement for SOD activity.

### SOD Activity

The SOD-like activity of Mn^2+^ complexes of the tripodal ligands was evaluated using the McCord-Fridovich assay.^28^ This is an indirect assay that examines the reduction of ferricytochrome c by superoxide ion in the absence and presence of SOD or potential SOD mimetic compounds. The reaction of xanthine with xanthine oxidase provides a source of superoxide ion for the assay, and the reduction of ferricytochrome c is monitored at 550 nm. Given their low to moderate aqueous stability, it was not possible to use preformed complexes for these assays since we would not expect them to remain intact at the micromolar concentrations employed. Instead, Mn^2+^ complexes were formed in situ, in buffered, aqueous solution (pH 7.5), by combining the ligands with manganese­(II) chloride. In each case, an excess of ligand was included to produce Mn^2+^ complexation as close to 100% as possible, while avoiding solubility issues. Percent complexation of the complexes under these conditions has been estimated from the 1:1 [MnL]^2+^ stability constants (β_MnL_) in Table 2, based on the ligand complex concentration employed and an Mn^2+^ concentration close to the IC_50_ values of the complexes (0.1 μM). We note that, given the high concentrations of ligand present in our experiments, higher speciation complexes (e.g., [MnL_2_]^2+^) may also be present in our reaction solutions, resulting from adventitious mono-or bidentate-coordination of an additional ligand. These types of species have been observed previously for Mn^2+^ complexes with a related ligand (HPClNOL), where HPClNOL is the tripodal ligand {1-[bis­(pyridine-2-ylmethyl)­amino]-3-chloropropan-2-ol}, yet in a 50:50, MeOH/H_2_O solution where overall complex stability is expected to be much higher.^36^ Since we cannot rule out higher speciation under the conditions of these in situ experiments, our use of the term Mn^2+^ complexes is meant to indicate all [MnL]^2+^ or [MnL_*x*_]^2+^ species present in reaction solutions here.

We also chose to run the assay in HEPES buffer to avoid the “phosphate effect,” whereby free Mn^2+^ is reported to exhibit SOD activity in phosphate buffer, but not in HEPES buffer.^37,38^ We in fact do not observe measurable SOD activity of MnCl_2_ in HEPES buffer in the range of the IC_50_ values of the complexes in our study (see below). Hence, the activity observed in our experiments is presumably due to Mn^2+^ complexes of our ligands only, and not to the presence of any free Mn^2+^ ion. Notably, we do observe SOD activity of MnCl_2_ in HEPES buffer at higher concentrations with an IC_50_ value of 13.0 μM (Table 4). This is about 30 times higher than the IC_50_ value of 0.47 μM that we measure for MnCl_2_ in phosphate buffer. Percent inhibition plots for MnCl_2_ in both HEPES and phosphate buffer are provided in Supporting Information (Figures S35 and S36).

**Table 4:** SOD Activity Data for Mn^2+^ Complexes Formed in situ in HEPES Buffer, pH 7.5

ligand or MnCl_2_	N/O	measured IC_50_ (μM)	conditional complex formation	apparent IC_50_ (μM)	*k*_cat_ (M^–1^ s^–1^)
TMPA	N4	0.30	99%	0.30	4.3 × 10^7^
DPEA	N3O	0.15	92%	0.14	9.3 × 10^7^
DPMEA	N3O	0.60	80%	0.48	2.7 × 10^7^
PDEA	N2O2	0.11	66%	0.07	1.9 × 10^8^
PDMEA]	N2O2	1.3	61%	0.79	1.6 × 10^7^
MnCl_2_	N/A	13.0	N/A	N/A	1.0 × 10^6^
MnCl_2_ ^a^	N/A	0.47	N/A	N/A	2.8 × 10^7^

a The second measurement of MnCl_2_ was done in 50 mM phosphate buffer, pH 7.5.

For each individual data point of our assay experiments, reduction of ferricytochrome c in HEPES buffer containing the specified concentration of ligand was first monitored in the absence of Mn^2+^ for a period of ∼2.5 min. Following this period, MnCl_2_ was added from a stock solution also containing the specified concentration of ligand, and the reaction was continued for an additional 2.5 min. Figure 7 shows the plot of a typical data set where the initial velocity (*V*_0_) is obtained from the slope prior to MnCl_2_ addition, and velocity in the presence of complex (*V*_c_) is obtained from the slope after the addition. Doing the experiment this way ensures that the presence of higher concentrations of the ligands does not interfere with the enzymatic production of superoxide ion or inhibit ferricytochrome c reduction, and that the latter is only brought on by the formation of Mn^2+^ complexes of the ligands in solution.

**Figure 7:**
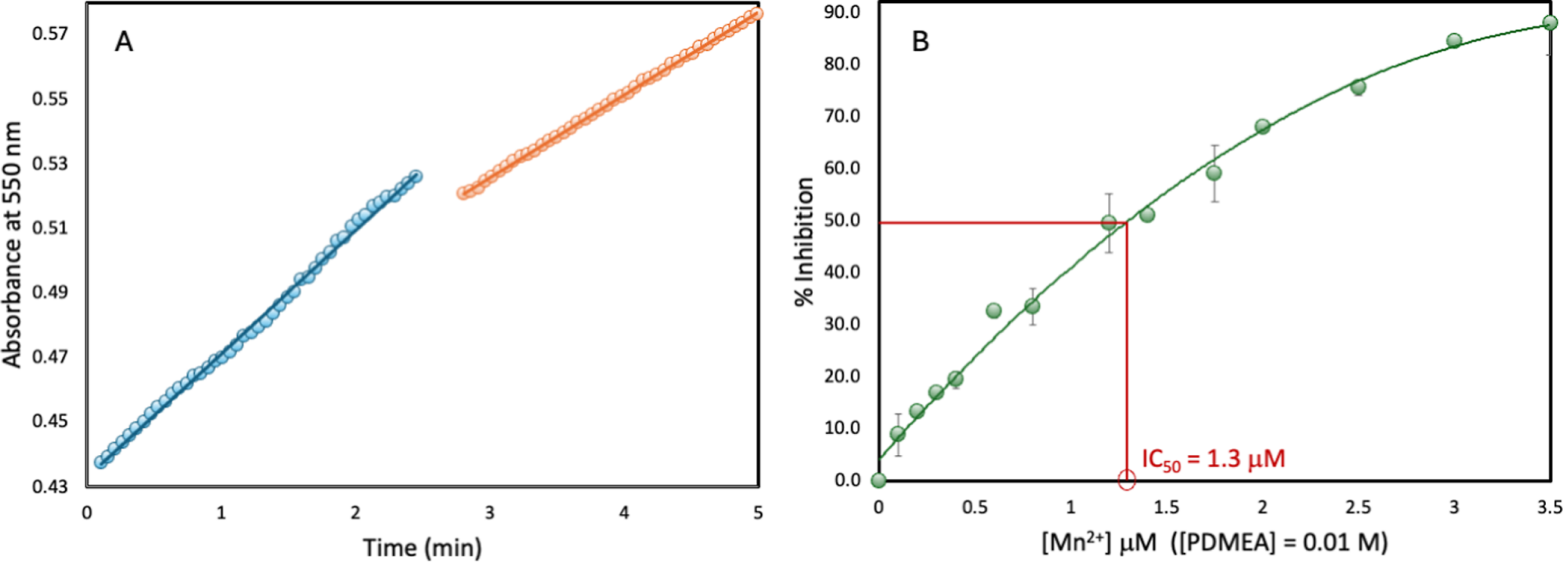
A typical data set of the McCord-Fridovich assay is shown in A, where the change in absorbance at 550 nm, due to ferricytochrome c reduction by superoxide ion, is plotted vs time. For this data set, 0.01 M PDMEA was present throughout, while an addition of MnCl_2_ was made after 2 min giving an Mn^2+^ concentration of 0.6 μM. Percent inhibition is given by the initial rate of ferricytochrome c reduction (V_0_) to the rate after MnCl_2_ addition (V_c_) using the equation $\%\;inhibition=\frac{\left(V_{0}-V_{c}\right)}{V_{0}}\times 100\%$ . Plot B shows the percent inhibition of ferricytochrome c reduction versus Mn^2+^ concentration in HEPES buffer, pH 7.5 containing PDMEA at an excess concentration of 0.01 M, at 25 °C. Each data point was run in triplicate, and error bars represent the standard deviation.

The Mn^2+^ complexes of each ligand, excluding TEA which does not form stable Mn^2+^ complexes in aqueous solution, promote significant SOD-like activity. A representative plot of the percent inhibition of ferricytochrome c reduction versus concentration of Mn^2+^ in excess ligand is shown in Figure 7. Percent inhibition plots for the other complexes and MnCl_2_ are provided in Supporting Information (Figures S35–S41). Both measured and apparent inhibition constants at 50% (IC_50_) values are listed in Table 4, the latter taking into account the expected percent formation of the complexes in the reaction solutions, where ${IC}_{50}\;\left(apparent\right)=\frac{{IC}_{50}\;\left(measured\right)}{\left(\%\;complex\;formation/100\right)}$ . The catalytic rate constants (*k*_cat_) listed in Table 4 were estimated from the apparent IC_50_ values using $k_{cat}=\frac{k_{cytc}\left[cytc\right]}{{IC}_{50}}$ , where *k*_cytc_ = 2.6 × 10^5^ M^–1^ s^–1^ (measured at pH 7.8 and 21 °C)^39^ and [cyt *c*] = 5 × 10^–5^ M.

Given the likelihood that multiple complex species exist in the reaction solutions, *k*_cat_ values cannot be assigned to a single species. Nonetheless, it is clear that complexation of Mn^2+^ by the ligands promotes efficient SOD activity. The measured *k*_cat_ values are high among SOD mimetic compounds that have been studied previously. In a recent review, *k*_cat_ values ranging from 1.5 × 10^3^ to 1.6 × 10^9^ M^–1^ s^–1^ were reported for 87 different compounds. Among these compounds, only 22% had a *k*_cat_ value greater than 1 × 10^7^ M^–1^ s^–1^.^12^ We also note that an IC_50_ of 0.35 μM was reported previously for Mn­(HPClNOL)­Cl_2_,^36^ which is comparable to the value of 0.15 μM that we report here for the Mn^2+^ complexes of DPEA which is structurally similar to HPClNOL.

The *k*_cat_ values of the Mn^2+^ complexes are shown graphically in Figure 8, grouped according to the N/O donor content of their ligands. This plot suggests that the *k*_cat_ values correlate with N/O donor content within two separate groupings, those containing methoxy and those containing hydroxy (Figure 8). For those containing methoxy, *k*_cat_ values appear to decrease with increasing O-donor content and therefore decreasing redox potential (*E*_1/2_ in Table 3). This may suggest that activity within this series is improved by stabilization of the Mn^2+^ oxidation state, and that the rate-limiting step of their mechanism is therefore oxidation of superoxide ion (and concomitant reduction of Mn^3+^ to Mn^2+^). Similar behavior has been observed previously for Mn^3+^ porphyrin compounds,^40,41^ and is consistent with our observation that ligands with higher nitrogen content stabilize Mn^2+^. However, the opposite trend is observed for complexes with hydroxy ligands. For this group of complexes, an increase in oxygen content, and therefore number of hydroxide moieties, results in higher *k*_*cat*_ values. The significant difference in *k*_cat_ values between complexes with methoxy ligands and those containing hydroxy ligands is most evident among equivalent NxOy groupings, DPEA/DPMEA and PDEA/PDMEA where other factors (*E*_1/2_ and K_MnL_) are nearly the same. This indicates that hydroxide groups may play a significant role in their mechanism, perhaps through proton transfer and/or hydrogen bonding with the superoxide ion substrate. Indeed, the roles of hydrogen-bonding and proton-coupled electron transfer (PCET) in the mechanisms of manganese-containing superoxide dismutase and SOD biomimetic complexes have been discussed widely.^42–45^

**Figure 8:**
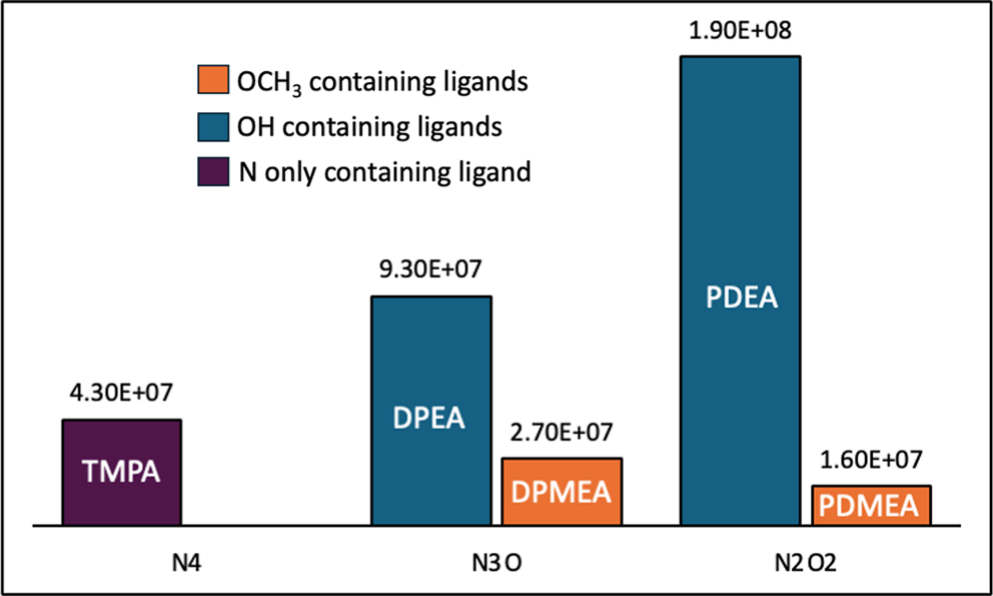
Column graph of the *k*_cat_ values for Mn^2+^ complexes grouped according to the N/O donor content of their ligand. Actual *k*_cat_ values (M^–1^ s^–1^) are shown above each column. Ligands containing methoxy or hydroxy groups are denoted by color, orange and blue respectively, while the N-donating only ligand, TMPA is shown in purple.

## Conclusion

The series of tripodal ligands studied herein demonstrate that ligands with high nitrogen donor content help stabilize Mn^2+^ complex formation in aqueous solution. This is revealed not only by potentiometric titrations, but also by the positive correlation between nitrogen content and Mn­(III/II) reduction potential, *E*_1/2_. The SOD activity of the Mn^2+^ complexes of these ligands correlates negatively with their oxygen content, but only when oxygen donating moieties are methoxy groups. For complexes with ligands that contain hydroxide groups, SOD activity increases with the number of hydroxy groups present. The dramatic difference in *k*_cat_ values between complexes with ligands containing methoxy and those containing hydroxy lead us to believe that the later may be a useful target for incorporation in future biomimetic SOD compounds.

## Supplementary Material



## Notes

^1^H and ^13^C NMR spectra of the ligands; IR spectra of the ligands and complexes; X-ray crystallographic data and refinement details for the complexes; fitted potentiometric titration plots of the ligands in the absence and presence of Mn^2+^ along with speciation plots; cyclic voltammograms of the complexes; and McCord–Fridovich assay data (PDF)
