# Supplementary material for: Effects of Ligand N and O Donor Content on the Structure, Aqueous Stability, and SOD Activity of a Series of Mn2+ Complexes with Pyridine-Containing Tripodal Ligands
Source: ACS Omega. 2025 Nov 14;10(46):56817–29. doi: 10.1021/acsomega.5c10215 (PMC12658643; doi:10.1021/acsomega.5c10215)
Supplement: Supplementary file 1 [file ao5c10215_si_001.pdf]

Supporting Information for

Effects of Ligand N and O Donor Content on the Structure, Aqueous Stability, and SOD Activity of a Series of Manganese(II) Complexes with Pyridine-Containing Tripodal Ligands

Steven T. Frey,[†] Haley A. Cirka,[†] Jasper G. Ballot,[†] Katheryn C. Rinaolo,[†] Nich Nearyrat Phalkun,[†] Anthony Mirkovic,[†] Aidan Spengler,[†] Avery J. Stamps,[†] Jacob R. Hale,[†] Yuta Nakagawa,[†] Hanyu Ruan,[†] Haiyang Lin,[†] Ian T. Frey,[†] Samuel J. Frey,[†] and Peter J. Bonitatibus.[‡]*

[†]Department of Chemistry, Skidmore College, 815 North Broadway, Saratoga Springs, NY 12866

[‡]Department of Chemistry, Rensselaer Polytechnic Institute, 110 Eighth Street, Troy, NY 12180

Contents

^{1}H and ^{13}C Spectra……………………………………………………2

IR Spectra……………………………………………………………………7

X-ray crystallographic data………………………………………7

Potentiometric Titration data…………………………………53

Cyclic voltammetry data…………………………………………60

McCord-Fridovich assay data…………………………………66

^{1}H and ^{13}C Spectra of the Tripodal ligands

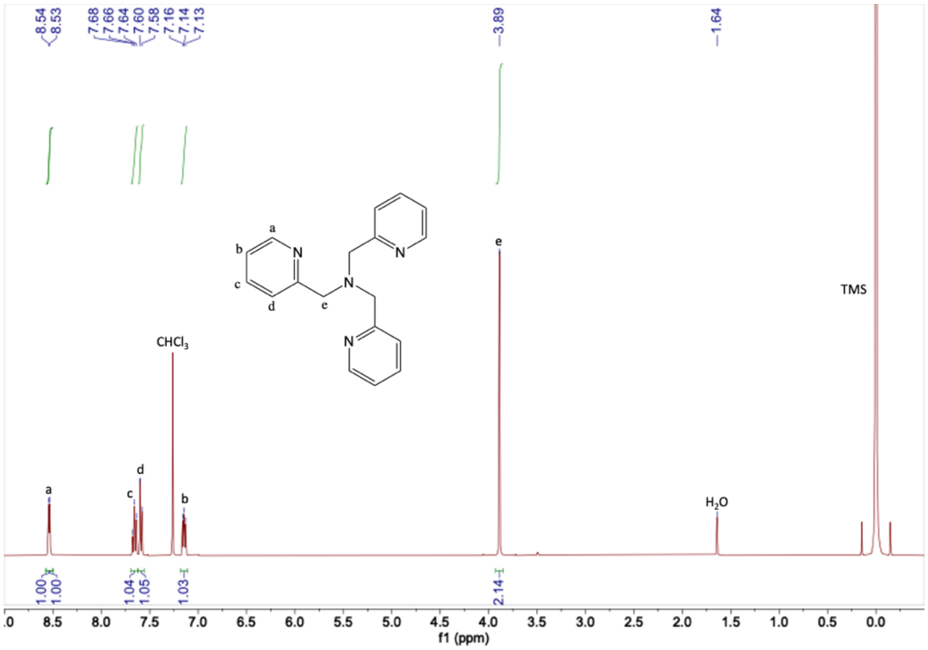

Figure S1. ^{1}H NMR (400 MHz, $CDCl_3$) of Tris(pyridin-2-ylmethyl)amine (TMPA) referenced to TMS: δ 8.54 (d, *J* = 4.8 Hz, 3H), 7.66 (t, *J* = 7.6 Hz, 3H), 7.59 (d, *J* = 6.6 Hz, 3H), 7.14 (t, *J* = 6.3 Hz, 3H), 3.89 (s, 6H). Water in the sample was present in the $CDCl_3$ solvent.

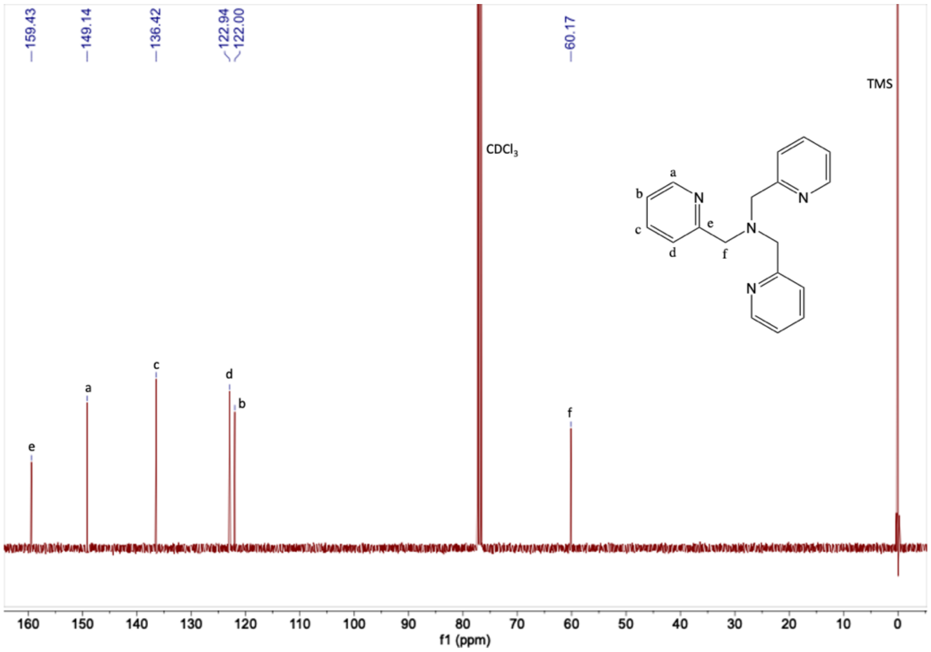

Figure S2. ^{13}C NMR (101 MHz, $CDCl_3$) of Tris(pyridin-2-ylmethyl)amine (TMPA) referenced to TMS: δ 159.43, 149.14, 136.42, 122.94, 122.00, 60.17.

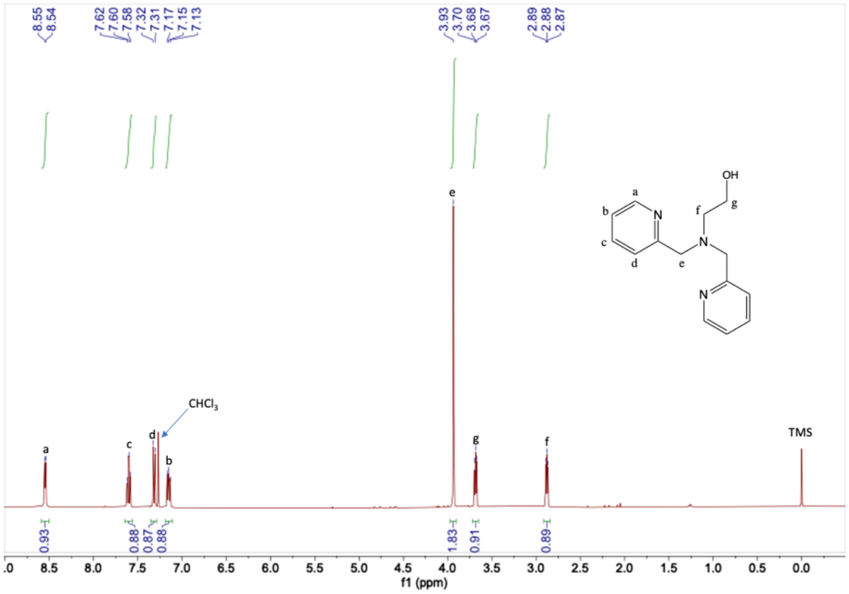

Figure S3. ^{1}H NMR (400 MHz, $CDCl_3$) of N,N-bis(2-pyridylmethyl)ethanolamine (DPEA) referenced to TMS: δ 8.55 (d, *J* = 4.9 Hz, 2H), 7.60 (t, *J* = 7.6 Hz, 2H), 7.32 (d, *J* = 7.8 Hz, 2H), 7.15 (t, *J* = 6.6 Hz, 2H), 3.93 (s, 4H), 3.70 (t, *J* = 4.8 Hz, 2H), 2.88 (t, *J* = 5.2 Hz, 2H).

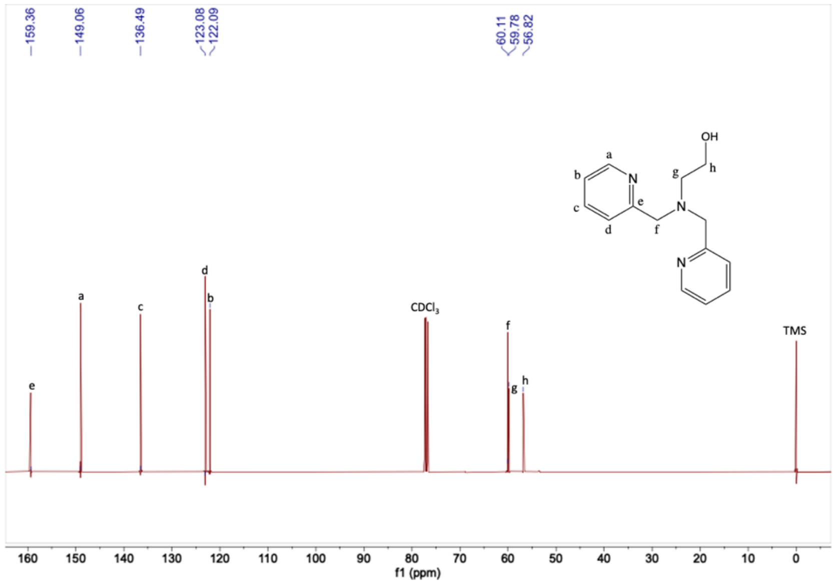

Figure S4. ^{13}C NMR (101 MHz, $CDCl_3$) of N,N-bis(2-pyridylmethyl)ethanolamine (DPEA) referenced to TMS: δ 159.36, 149.06, 136.49, 123.08, 122.09, 60.11, 59.78, 56.82.

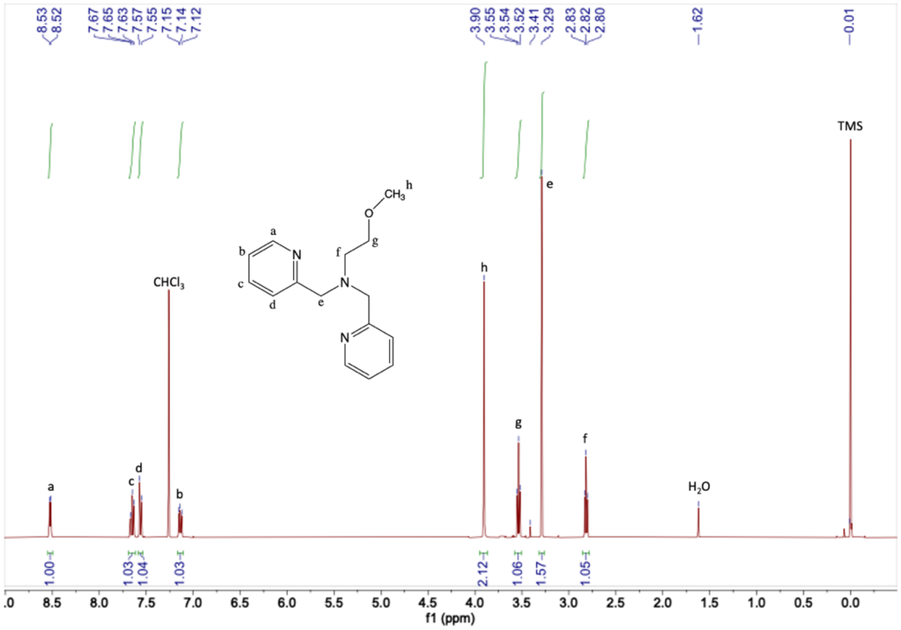

Figure S5. ^{1}H NMR (400 MHz, $CDCl_3$) of 2-Methoxy-N,N-bis(pyridin-2-ylmethyl)ethan-1-amine (DPMEA) referenced to TMS: δ 8.52 (d, *J* = 4.8 Hz, 2H), 7.65 (t, *J* = 7.6 Hz, 2H), 7.56 (d, *J* = 7.9 Hz, 2H), 7.13 (t, *J* = 6.0 Hz, 2H), 3.90 (s, 4H), 3.54 (t, *J* = 5.8 Hz, 2H), 3.29 (s, 3H), 2.82 (t, *J* = 5.8 Hz, 2H). Water in the sample was present in the $CDCl_3$ solvent.

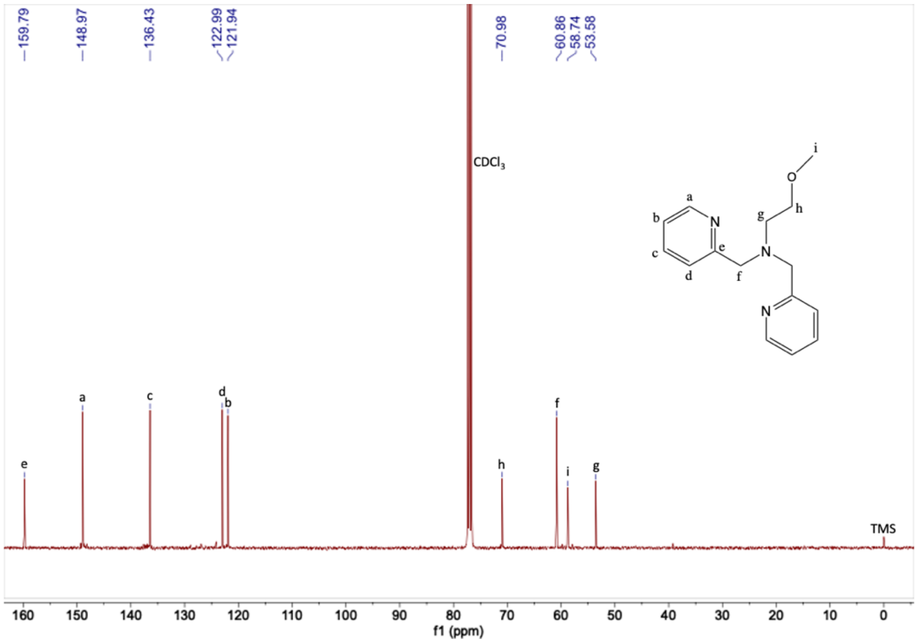

Figure S6. ^{13}C NMR (101 MHz, $CDCl_3$) of 2-Methoxy-N,N-bis(pyridin-2-ylmethyl)ethan-1-amine (DPMEA) referenced to TMS: δ 159.36, 149.06, 136.49, 123.08, 122.09, 60.11, 59.78, 56.82.

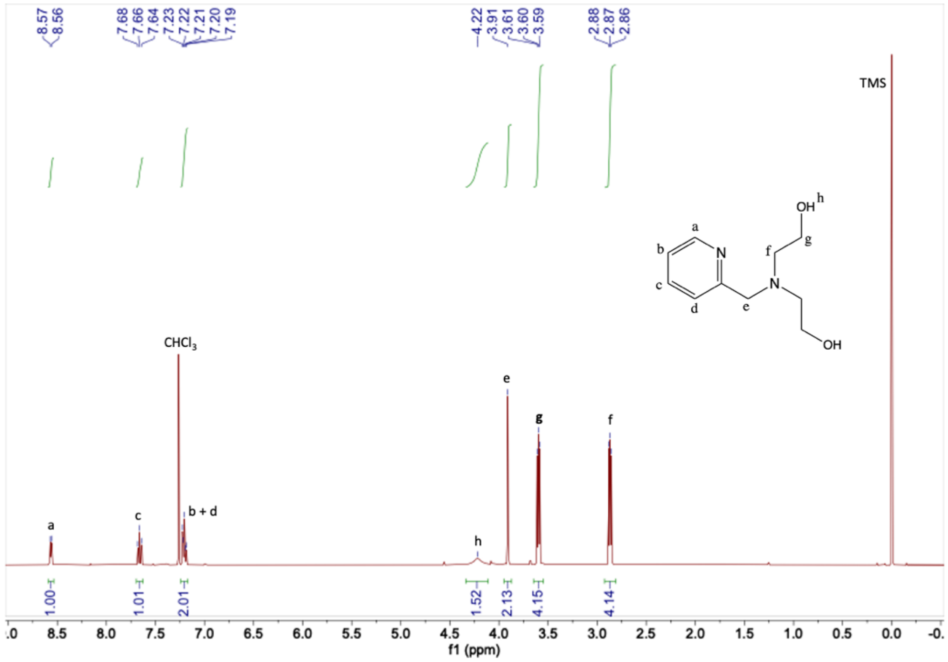

Figure S7. ^{1}H NMR (400 MHz, $CDCl_3$) of 2,2'-((Pyridin-2-ylmethyl)azanediyl)bis(ethan-1-ol) (PDEA) referenced to TMS: δ 8.57 (d, *J* = 4.9 Hz, 1H), 7.66 (t, *J* = 7.6 Hz, 1H), 7.24 – 7.17 (m, 2H), 4.22 (s, 2H), 3.91 (s, 2H), 3.59 (t, *J* = 4.8 Hz, 4H), 2.85 (t, *J* = 5.2 Hz, 4H).

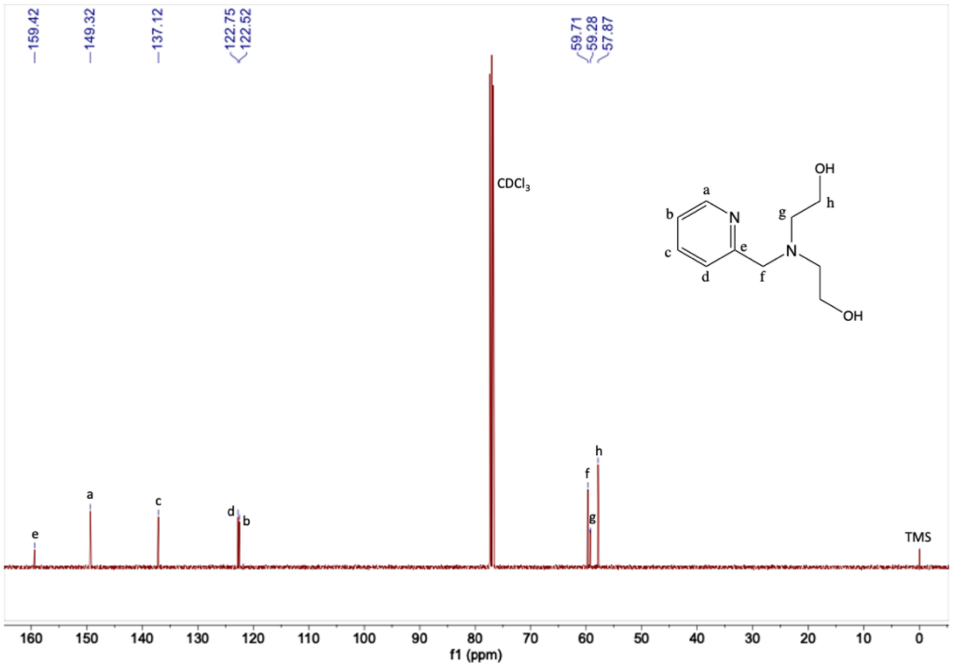

Figure S8. ^{13}C NMR (101 MHz, $CDCl_3$) of 2,2'-((Pyridin-2-ylmethyl)azanediyl)bis(ethan-1-ol) (PDEA) referenced to TMS: δ 159.42, 149.32, 137.12, 122.75, 122.52, 59.71, 59.28, 57.87.

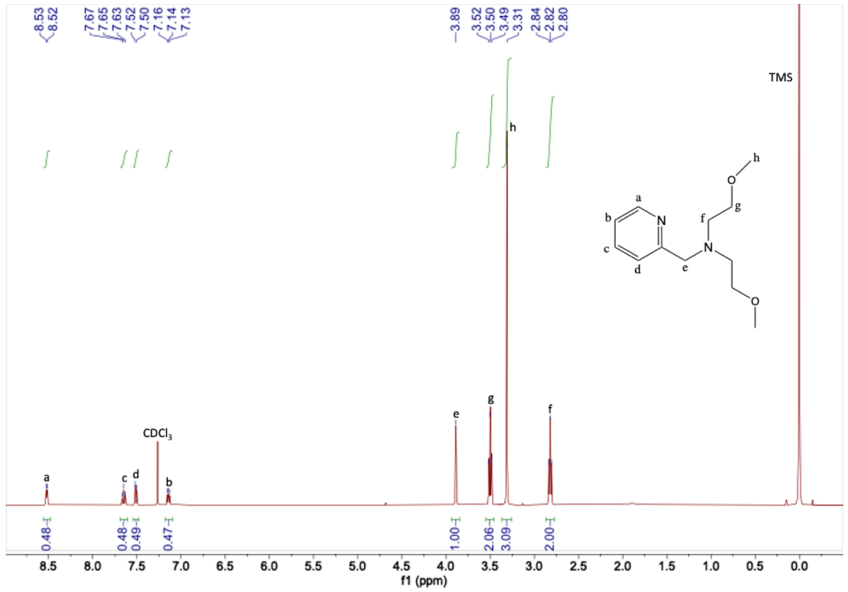

Figure S9. ^{1}H NMR (400 MHz, $CDCl_3$) of 2-Methoxy-N-(2-methoxyethyl)-N-(pyridin-2-ylmethyl)ethan-1-amine (PDMEA) referenced to TMS: δ 8.50 (d, *J* = 4.9 Hz, 1H), 7.63 (t, *J* = 8.1 Hz, 1H), 7.49 (d, *J* = 7.8 Hz, 1H), 7.12 (t, *J* = 6.0 Hz, 1H), 3.87 (s, 2H), 3.48 (t, *J* = 6.5 Hz, 4H), 3.29 (s, 3H), 2.80 (t, *J* = 6.0 Hz, 4H).

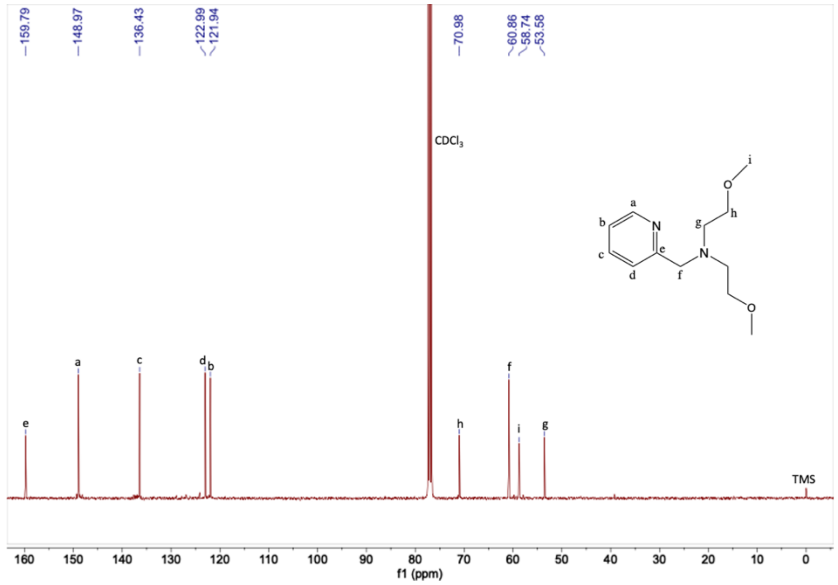

Figure S10. ^{13}C NMR (101 MHz, $CDCl_3$) of 2-Methoxy-N-(2-methoxyethyl)-N-(pyridin-2-ylmethyl)ethan-1-amine (PDMEA) referenced to TMS: δ 159.79, 148.97, 136.43, 122.99, 121.94, 70.98, 60.86, 58.74, 53.58.

FT-IR Spectra of Ligands and Complexes

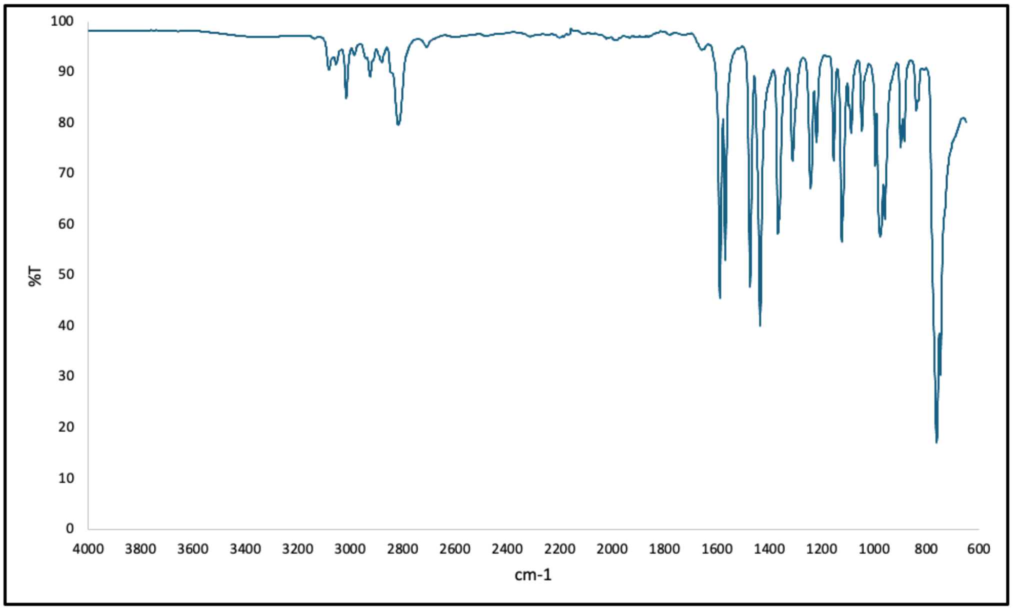

Figure S11. IR (ATR, cm^{-1}) of Tris(pyridin-2-ylmethyl)amine (**TMPA**): 3081-2708 (m, ν_{C-H}), 1588, 1564, 1474, 1436 (s, ν_{pyr}).

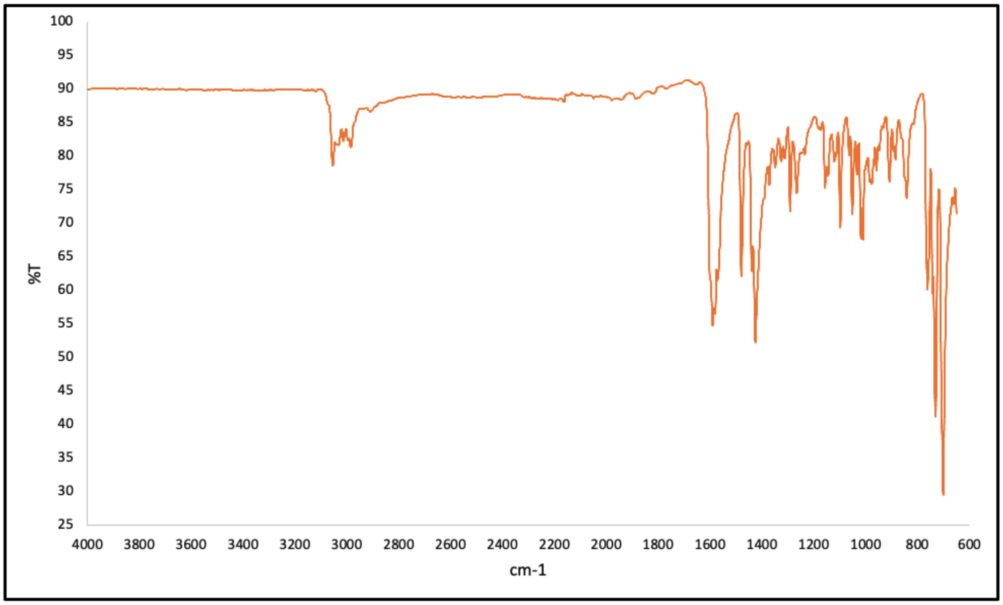

Figure S12. IR (ATR, cm^{-1}) of [Mn(TMPA)(OAc)]BPh_4**:** 3200-2800 (m, ν_{C-H}), 1529, 1416 (s, ν_{C-O}), 704, 729 (s, ν_{B-C}).

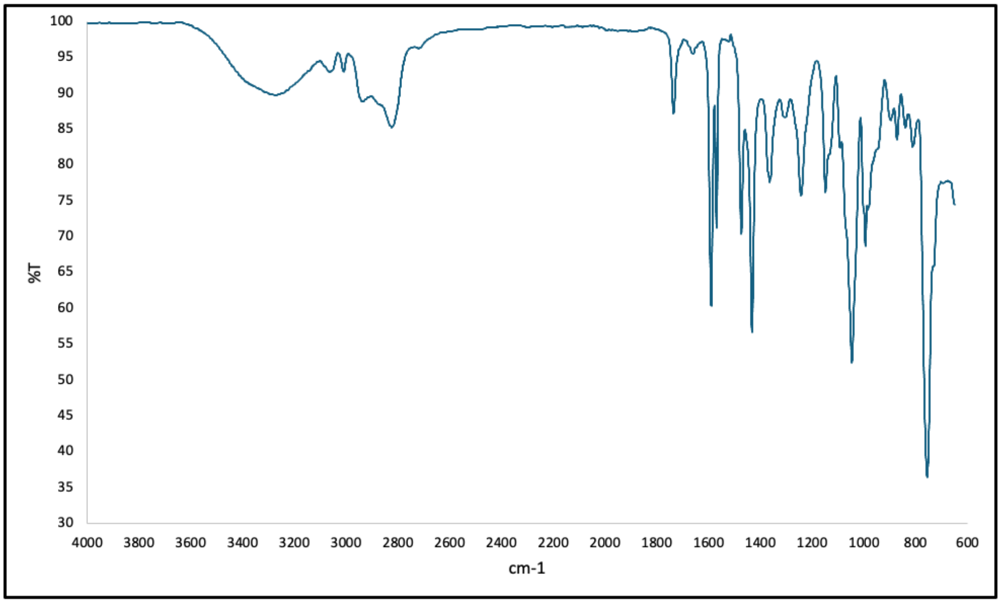

Figure S13. IR (ATR, cm^{-1}) of N,N-bis(2-pyridylmethyl)ethanolamine (**DPEA**): 3293 (b, $\nu_{O\text{-}H}$), 3030-2820 (m, $\nu_{C\text{-}H}$), 1589, 1569, 1474, 1432 (s, ν_{pyr}), 1046 (s, $\nu_{C\text{-}OH}$).

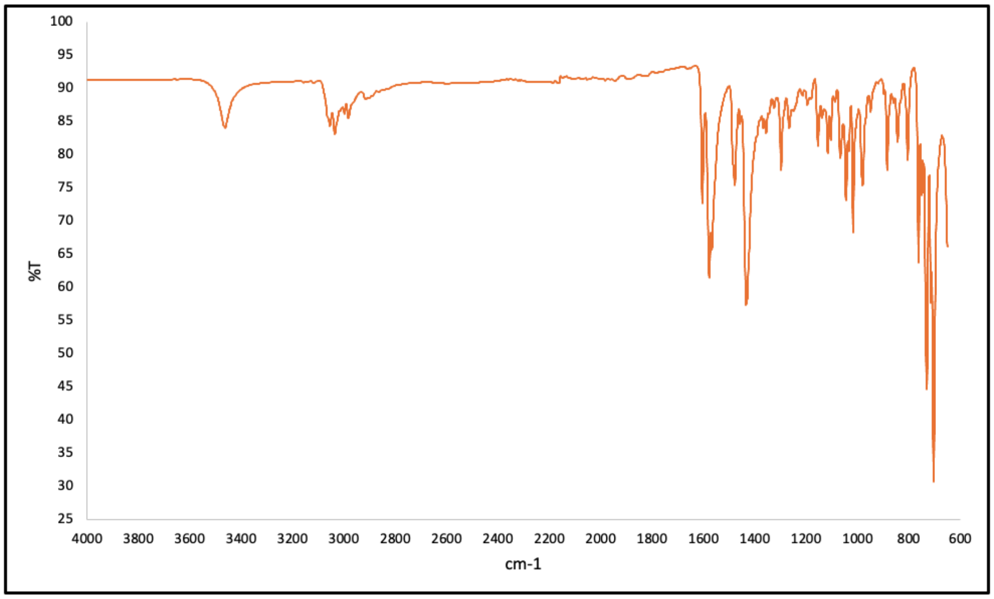

Figure S14. IR (ATR, cm^{-1}) of [Mn(DPEA)(OAc)]BPh_4: IR (ATR, cm^{-1}): 3437 (b, $\nu_{O\text{-}H}$), 3200-2800 (m, $\nu_{C\text{-}H}$), 1580, 1418 (s, $\nu_{C\text{-}O}$), 734, 707 (s, $\nu_{B\text{-}C}$).

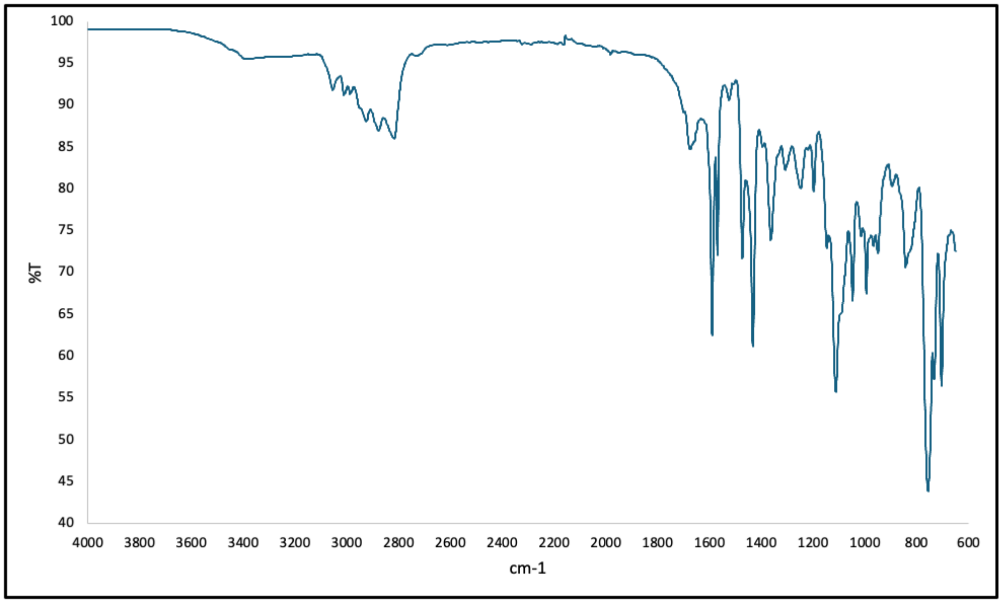

Figure S15. IR (ATR, cm^{-1}) of 2-Methoxy-N,N-bis(pyridin-2-ylmethyl)ethan-1-amine (**DPMEA**): 3200-2800 (m, ν_{C-H}), 1589, 1569, 1473, 1432 (s, ν_{pyr}), 1112 (s, ν_{C-OH}).

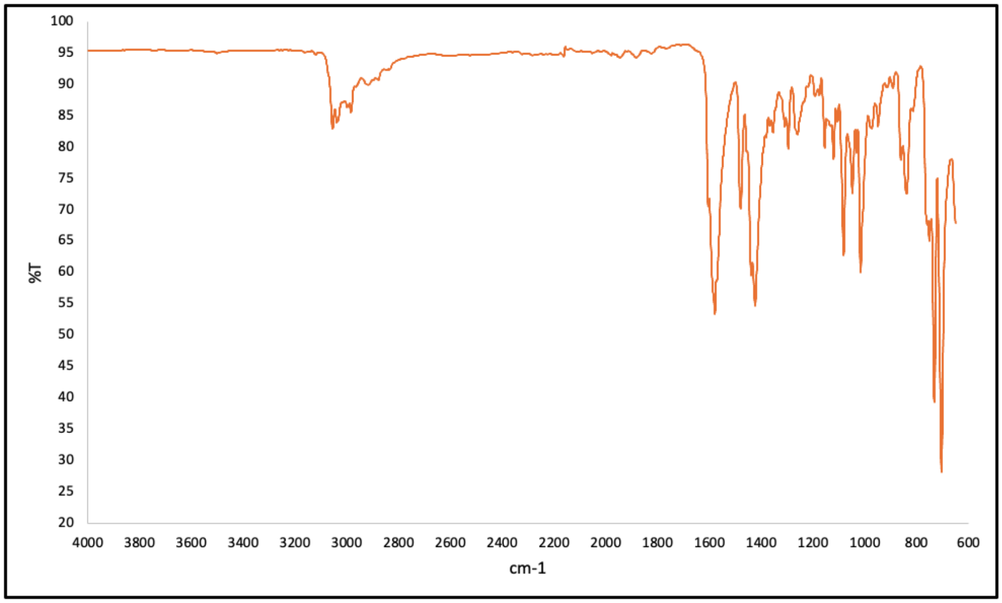

Figure S16. IR (ATR, cm^{-1}) of $[Mn(DPMEA)(OAc)]BPh_4$: 3200-2800 (m, ν_{C-H}), 1605, 1578 (s, νC-O), 758, 704 (s, ν_{B-C}).

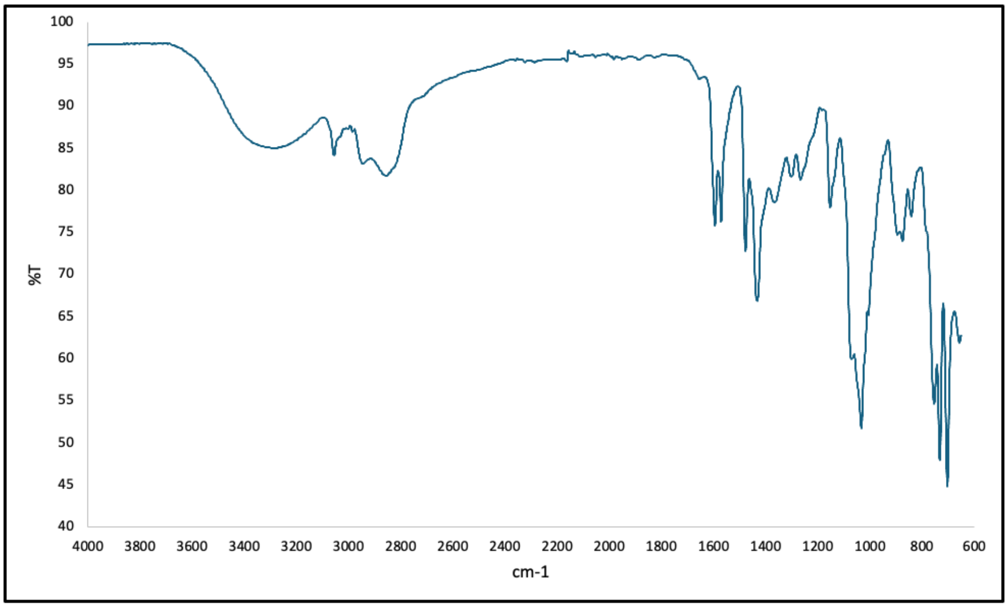

Figure S17. IR (ATR, cm^{-1}) of 2,2'-((Pyridin-2-ylmethyl)azanediyl)bis(ethan-1-ol) (**PDEA**): 3284 (b, $\nu_{O\text{-}H}$), 3030-2820 (m, $\nu_{C\text{-}H}$), 1595, 1571, 1478, 1432 (s, ν_{pyr}), 1033 (s, $\nu_{C\text{-}OH}$).

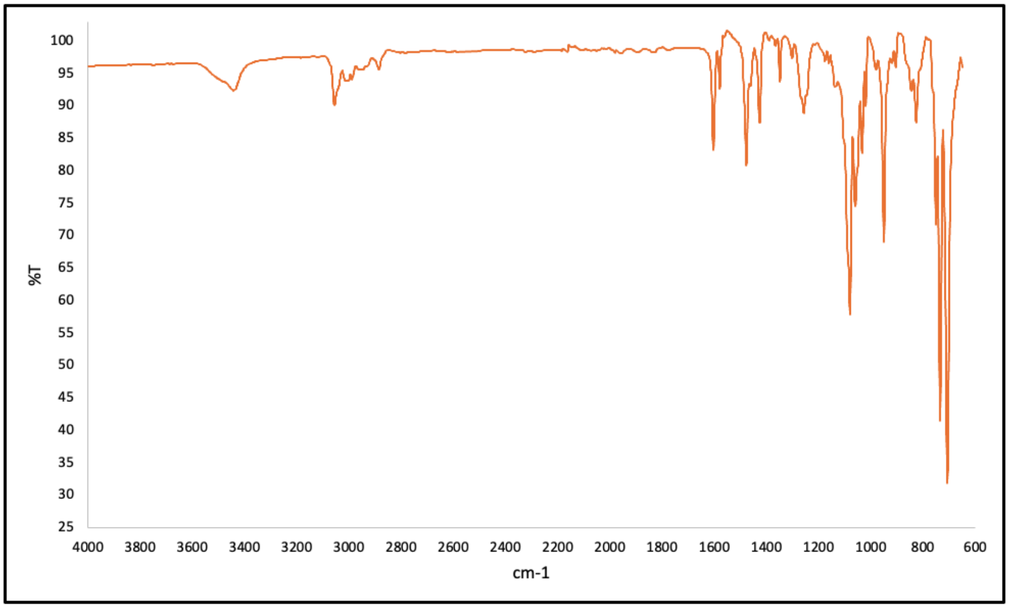

Figure S18. IR (ATR, cm^{-1}) of $[Mn(PDEA)(OAc)]BPh_4$: 3200-2800 (m, $\nu_{C\text{-}H}$), 1578, 1426 (s, $\nu_{C\text{-}O}$), 732, 704 (s, $\nu_{B\text{-}C}$).

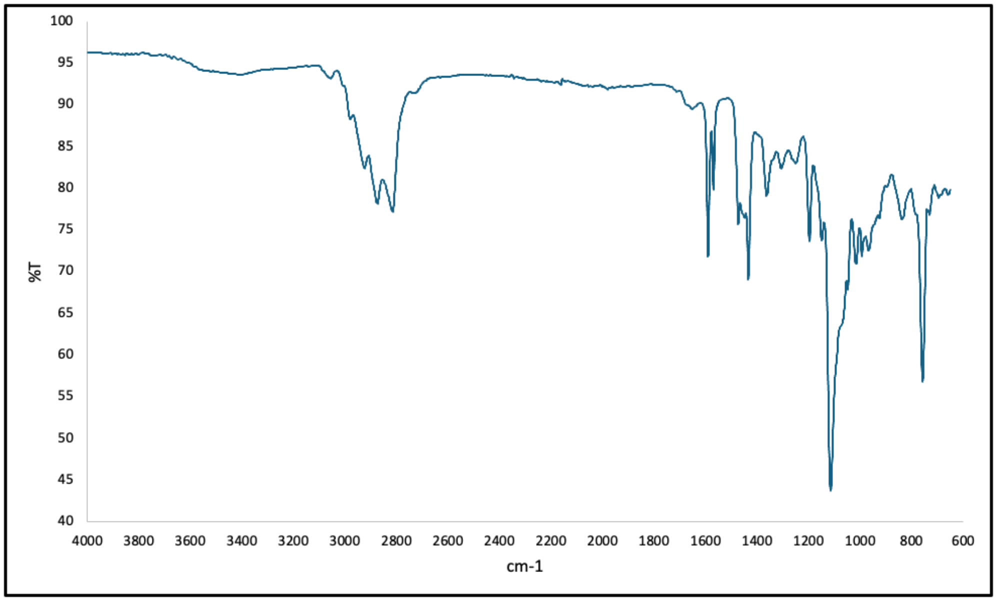

Figure S19. IR (ATR, cm^{-1}) of 2-Methoxy-N-(2-methoxyethyl)-N-(pyridin-2-ylmethyl)ethan-1-amine (**PDMEA**): 3200-2800 (m, ν_{C-H}), 1587, 1568, 1472, 1431 (s, ν_{pyr}), 1114 (s, ν_{C-OH}).

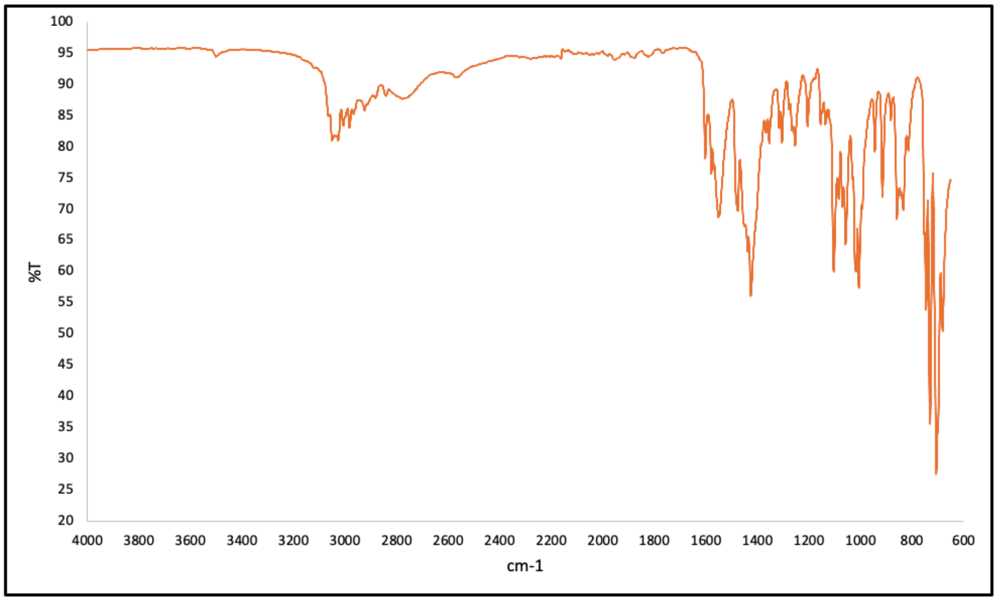

Figure S20. IR (ATR, cm^{-1}) of [Mn(PDMEA)(OAc)]BPh_4: 3200-2800 (m, ν_{C-H}), 1548, 1426 (s, ν_{C-O}), 731, 706 (s, ν_{B-C}).

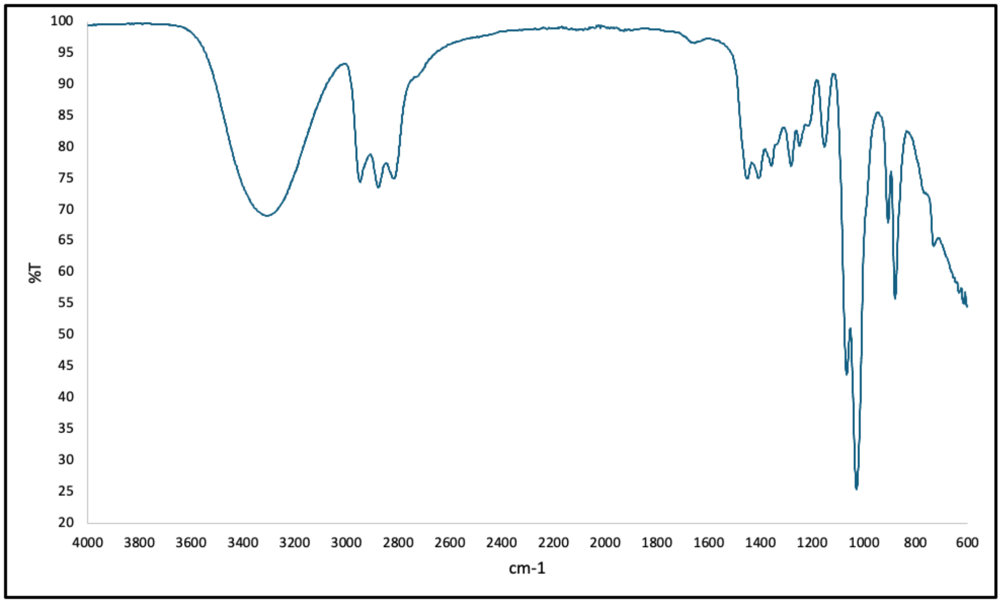

Figure S21. IR (ATR, cm^{-1}) of 2,2',2''-nitrilotris(ethan-1-ol) (**TEA**)**:** 3300 (b, ν_{O-H}), 3000-2800 (m, ν_{C-H}), 1029 (s, ν_{C-OH}).

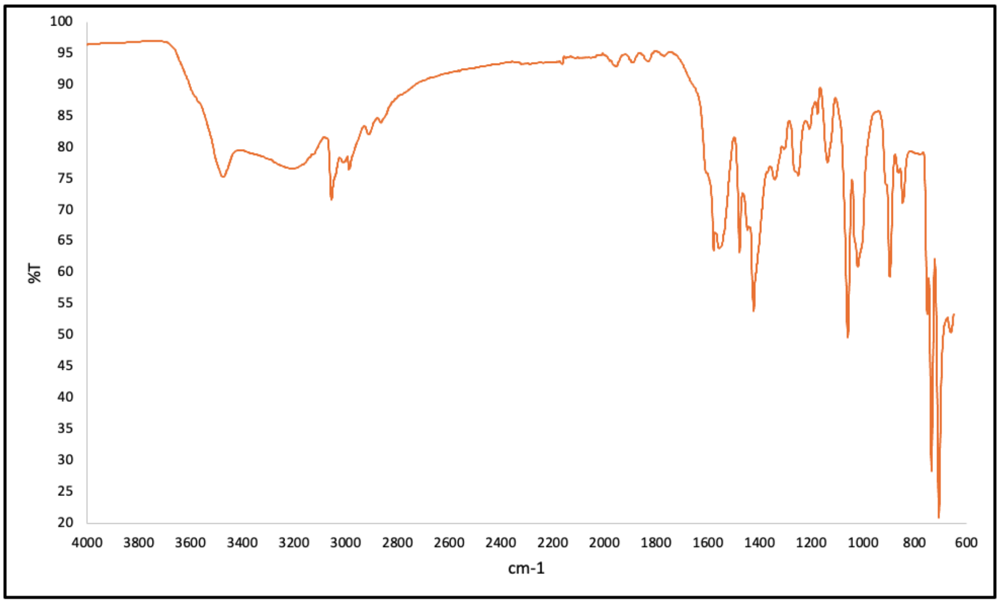

Figure S22. IR (ATR, cm^{-1}) of $[Mn(TEA)(OAc)]BPh_4$: 3600-3100 (b, ν_{O-H}), 3000-2800 (m, ν_{C-H}), 1578, 1423 (s, ν_{C-O}), 735, 707 (s, ν_{B-C}).

Crystal Data and Refinement Details

Crystal data and refinement details for **[Mn(DPEA)(OAc)(MeOH)]BPh_4·MeOH** are as follows: triclinic *a* = 12.1807(6) Å, *b* = 12.8728(7) Å, *c* = 13.0961(8) Å, with α = 74.486(5)°, β = 86.350(4)°, γ = 77.779(4) ° (at 173 K), and the space group is $P\bar{1}$. The asymmetric unit consists of one molecule of methanol. In the primary coordination sphere of Mn, the acetate ligand features hydrogen-bonding interactions with hydrogen atoms of ligands on either side of it, namely H17b of the methyl group (bound methanol) and H14 of a pyridine ring. A total of 25,976 reflections were measured between 6.456° ≤ 2θ ≤ 65.544° (MoKα), of which 12,745 were unique (R_{int} = 0.0290, R_s = 0.0466). The final R_1 was 0.0448 (I > 2σ (I)) and wR_2 was 0.1001. CCDC 1948836.

Crystal data and refinement details are as follows: for **[Mn(DPMEA)(OAc)(MeOH)]BPh_4·2 MeOH**, the unit cell is triclinic *a* = 10.7350(6) Å, *b* = 12.0836(5) Å, *c* = 16.8786(6) Å, with α = 78.775(3)°, β = 88.865(4)°, γ = 74.451(5)° (at 173 K), and the space group is $P\bar{1}$. The asymmetric unit consists of two molecules of methanol hydrogen-bonded to ligands in the primary coordination sphere of the Mn ion. The ethylene linker of the ligand was disordered (C17 and C18) and modeled with 45%/55% atomic occupancy (C17/C17A and C18/C18A). A total of 17,923 reflections were measured between 6.148° ≤ 2θ ≤ 54.998° (MoKα), of which 9,464 were unique (R_{int} = 0.0312, R_s = 0.0484). The final R_1 was 0.0476 (I > 2σ (I)) and wR_2 was 0.1250. CCDC 2431198.

For **[Mn(PDEA)(OAc)(MeOH)]BPh_4**, a dataset was collected and processed as described above (299.2 K) using a suitable crystal. Crystal data are as follows: triclinic $P\bar{1}$, *a* = 10.63990(10) Å, *b* = 11.7269(2) Å, *c* = 15.7331(3) Å, with α = 111.191(2)°, β = 95.4590(10)°, and γ = 90.3140(10)°. A

total of 32,924 reflections were measured between 6.058° ≤ 2θ ≤ 137° (Cu$K\alpha$), of which 6,666 were unique (R_{int} = 0.0573, R_s = 0.0418). The final R_1 was 0.0481 (I > 2σ (I)) and wR_2 was 0.1151. A solvent mask was required to handle two methanol molecules. CCDC 2431199.

Crystal data and refinement details for **[Mn(PDMEA)(OAc)(MeOH)]BPh$_4$** are as follows: the unit cell is triclinic: *a* = 11.3885(5) Å, *b* = 12.0841(5) Å, *c* = 14.7728(7) Å, with α = 66.057(4)°, β = 82.169(4)°, γ = 71.989(4)° (at 173 K), and the space group is P$\overline{1}$. A total of 20,687 reflections were measured between 4.562° ≤ 2θ ≤ 65.672° (Mo$K\alpha$), of which 11,634 were unique (R_{int} = 0.0278, R_s = 0.0437). The final R_1 was 0.0431 (I > 2σ (I)) and wR_2 was 0.1057. CCDC 2431203.

For **[Mn(TEA)(OAc)(MeOH)]BPh$_4$·2 MeOH**, a dataset was collected and processed as described above (298.82.2 K) using a suitable crystal. Crystal data are as follows: monoclinic P2$_1$/c, *a* = 10.0738(2) Å, *b* = 14.8304(3) Å, *c* = 24.6141(4) Å, with α = 99.3840(10)°. A total of 34,992 reflections were measured between 6.984° ≤ 2θ ≤ 154.806° (Cu$K\alpha$), of which 7,238 were unique (R_{int} = 0.0325, R_s = 0.0242). The final R_1 was 0.0402 (I > 2σ (I)) and wR_2 was 0.1009. The asymmetric unit consists of two molecules of methanol. CCDC 2431204.

X-ray crystallographic tables for [Mn(DPEA)(OAc)(MeOH)]BPh_4·MeOH (2)

Table S1. Crystal data and structure refinement for [Mn(DPEA)(OAc)(MeOH)]BPh_4·MeOH (2).

Identification code	**MnDPEA**
Empirical formula	$C_{42}H_{48}BMnN_3O_5$
Formula weight	740.58 g/mol
Temperature/K	173(2)
Crystal system	triclinic
Space group	$P\bar{1}$
a/Å	12.1807(6)
b/Å	12.8728(7)
c/Å	13.0961(8)
α/°	74.486(5)
β/°	86.350(4)
γ/°	77.779(4)
Volume/$Å^3$	1933.77(18)
Z	2
$\rho_{calc}g/cm^3$	1.272
μ/mm^{-1}	0.389
F(000)	782.0
Crystal size/mm^3	0.38 × 0.34 × 0.24
Radiation	Mo Kα (λ = 0.71073)
2Θ range for data collection/°	6.456 to 65.544
Index ranges	-17 ≤ h ≤ 18, -19 ≤ k ≤ 19, -19 ≤ l ≤ 19
Reflections collected	25976
Independent reflections	12745 [R_{int} = 0.0290, R_{sigma} = 0.0466]
Data/restraints/parameters	12745/6/482
Goodness-of-fit on F^2	1.022
Final R indexes [I>=2σ (I)]	R_1 = 0.0448, wR_2 = 0.1001
Final R indexes [all data]	R_1 = 0.0660, wR_2 = 0.1119
Largest diff. peak/hole / e $Å^{-3}$	0.36/-0.33

Table S2. Fractional Atomic Coordinates (×10^4) and Equivalent Isotropic Displacement Parameters (Å^2×10^3) for [Mn(DPEA)(OAc)(MeOH)]BPh_4·MeOH (2). U_{eq} is defined as 1/3 of the trace of the orthogonalised U_{IJ} tensor.

Atom	*x*	*y*	*z*	U(eq)
Mn1	6975.1(2)	5831.4(2)	4159.7(2)	21.44(6)
O1	5696.7(9)	4884.8(9)	3783.0(8)	27.5(2)
O2	8067.8(9)	4303.6(9)	3987.4(10)	37.0(3)
O3	6509.1(9)	4956.0(9)	5792.9(8)	28.2(2)
O4	7954.9(10)	5728.1(10)	5699.2(9)	34.4(2)
N1	5693.1(10)	7372.2(9)	4254.8(9)	24.2(2)
N2	6371.0(10)	6738.0(9)	2417.4(9)	22.4(2)
N3	8416.7(10)	6588.3(10)	3246.1(10)	27.0(2)
C1	5218.4(14)	7577.3(12)	5155.7(12)	31.2(3)
C2	4446.3(16)	8524.3(13)	5181.1(14)	39.0(4)
C3	4141.1(16)	9295.2(13)	4235.6(14)	39.1(4)
C4	4609.7(14)	9095.7(12)	3303.3(13)	30.9(3)
C5	5385.0(12)	8127.9(11)	3338.5(11)	23.1(3)
C6	5971.2(13)	7921.7(11)	2342.7(11)	26.5(3)
C7	5465.2(12)	6261.8(12)	2134.0(11)	26.5(3)
C8	5669.0(13)	5045.8(12)	2659.9(12)	28.5(3)
C9	7370.3(12)	6571.0(12)	1741.3(11)	25.9(3)
C10	8349.1(12)	6841.8(11)	2186.8(11)	24.2(3)
C11	9138.4(13)	7327.2(13)	1522.6(13)	31.9(3)
C12	10022.2(15)	7567.2(15)	1963.5(14)	39.1(4)
C13	10095.4(15)	7310.2(15)	3050.5(15)	40.2(4)
C14	9284.8(14)	6820.6(14)	3664.4(13)	34.9(3)
C15	7314.3(12)	5135.0(11)	6240.5(11)	24.4(3)
C16	7490.8(15)	4657.4(15)	7405.3(12)	37.5(4)
C17	7886.2(17)	3207.9(14)	4415.6(18)	48.9(5)
C18	5676.3(12)	1829.7(10)	1119.0(11)	22.6(3)
C19	5060.5(13)	2323.4(11)	1870.1(12)	27.0(3)
C20	3927.0(14)	2321.1(12)	2080.6(13)	32.4(3)
C21	3352.0(13)	1813.1(12)	1539.0(13)	32.1(3)
C22	3924.5(13)	1310.3(13)	800.2(12)	30.0(3)
C23	5062.1(12)	1320.6(12)	596.4(11)	25.9(3)
C24	7537.8(11)	1196.5(11)	21.7(11)	22.6(3)
C25	8360.7(13)	236.5(12)	140.3(13)	30.3(3)
C26	8780.2(15)	-180.0(14)	-718.8(15)	39.5(4)
C27	8378.5(15)	340.4(15)	-1728.8(14)	39.6(4)
C28	7555.7(14)	1287.9(15)	-1877.3(13)	34.7(3)

Table S2. Fractional Atomic Coordinates (×10^4) and Equivalent Isotropic Displacement Parameters (Å^2×10^3) for [Mn(DPEA)(OAc)(MeOH)]BPh_4·MeOH (2). U_{eq} is defined as 1/3 of the trace of the orthogonalised U_{IJ} tensor.

Atom	*x*	*y*	*z*	U(eq)
C29	7158.5(12)	1704.3(13)	-1019.0(12)	28.3(3)
C30	7335.4(12)	3021.0(11)	587.0(11)	23.8(3)
C31	6546.4(13)	4013.2(11)	371.4(11)	25.3(3)
C32	6847.8(14)	5034.4(12)	0.6(11)	28.2(3)
C33	7962.9(14)	5104.9(12)	-159.6(12)	30.8(3)
C34	8773.9(14)	4145.1(13)	31.0(13)	32.9(3)
C35	8458.0(13)	3130.0(12)	380.0(13)	30.6(3)
C36	7609.8(12)	1068.8(11)	2120.0(11)	24.3(3)
C37	8476.9(17)	1295.3(15)	2610.4(15)	42.7(4)
C38	8935.6(19)	632.2(17)	3571.7(16)	51.8(5)
C39	8530.9(17)	-290.2(15)	4094.0(14)	43.2(4)
C40	7665.4(16)	-538.9(14)	3645.1(14)	39.8(4)
C41	7220.4(14)	129.8(12)	2688.1(13)	32.7(3)
B1	7036.5(13)	1782.6(12)	971.2(12)	22.2(3)
O5	214.0(11)	3974.2(15)	3453.9(13)	55.5(4)
C42	553(2)	3340(3)	2750(2)	86.3(10)

Table S3. Anisotropic Displacement Parameters (Å^2×10^3) for [Mn(DPEA)(OAc)(MeOH)]BPh_4·MeOH (2). The Anisotropic displacement factor exponent takes the form: $-2\pi^2[h^2a^{*2}U_{11}+2hka^*b^*U_{12}+\ldots]$.

Atom	U_{11}	U_{22}	U_{33}	U_{23}	U_{13}	U_{12}
Mn1	19.22(10)	26.18(10)	19.32(10)	-4.99(7)	2.12(7)	-7.35(8)
O1	22.7(5)	35.5(5)	27.5(5)	-9.6(4)	2.4(4)	-11.5(4)
O2	23.4(6)	33.3(5)	56.1(8)	-15.9(5)	10.0(5)	-7.2(4)
O3	23.0(5)	40.3(6)	23.0(5)	-8.0(4)	1.8(4)	-11.0(4)
O4	31.1(6)	48.3(6)	25.1(5)	-3.0(5)	0.7(4)	-19.7(5)
N1	27.6(6)	24.7(5)	21.2(5)	-7.2(4)	2.4(5)	-6.3(5)
N2	20.1(5)	27.1(5)	21.0(5)	-8.0(4)	2.5(4)	-5.7(4)
N3	24.1(6)	34.7(6)	23.0(6)	-5.2(5)	2.1(5)	-11.5(5)
C1	41.2(9)	28.5(7)	23.9(7)	-8.1(5)	4.7(6)	-6.9(6)
C2	51.0(11)	34.1(8)	32.5(8)	-15.5(7)	11.4(8)	-4.2(7)
C3	43.5(10)	29.4(7)	42.0(9)	-13.5(7)	5.7(8)	1.6(7)
C4	32.8(8)	26.0(7)	31.7(8)	-6.0(6)	-1.0(6)	-2.9(6)
C5	23.9(7)	24.1(6)	23.2(6)	-7.0(5)	0.4(5)	-7.8(5)
C6	29.7(7)	25.6(6)	22.0(6)	-4.3(5)	1.6(6)	-3.5(5)

Table S3. Anisotropic Displacement Parameters ($Å^2 \times 10^3$) for [Mn(DPEA)(OAc)(MeOH)]BPh_4·MeOH (2). The Anisotropic displacement factor exponent takes the form: $-2\pi^2[h^2a^{*2}U_{11}+2hka^*b^*U_{12}+...]$.

Atom	U_{11}	U_{22}	U_{33}	U_{23}	U_{13}	U_{12}
C7	21.1(7)	36.6(7)	23.7(7)	-10.8(6)	-1.1(5)	-6.1(6)
C8	26.4(7)	36.5(7)	28.2(7)	-15.0(6)	2.0(6)	-10.7(6)
C9	24.6(7)	34.1(7)	20.0(6)	-8.6(5)	4.6(5)	-7.6(6)
C10	22.2(7)	25.6(6)	24.6(7)	-6.2(5)	4.6(5)	-6.3(5)
C11	31.4(8)	36.1(8)	27.2(7)	-4.5(6)	8.6(6)	-12.2(6)
C12	32.1(9)	45.4(9)	41.4(9)	-6.9(7)	11.6(7)	-20.4(7)
C13	30.4(9)	53.8(10)	42.1(10)	-12.4(8)	4.1(7)	-22.5(8)
C14	28.8(8)	49.4(9)	29.4(8)	-7.4(7)	0.7(6)	-17.8(7)
C15	22.0(7)	27.5(6)	21.9(6)	-4.7(5)	0.5(5)	-3.5(5)
C16	35.5(9)	50.9(10)	22.9(7)	-0.6(7)	-5.0(6)	-11.5(8)
C17	40.5(10)	33.4(8)	70.4(14)	-11.7(9)	2.7(9)	-5.0(8)
C18	22.7(6)	20.8(6)	22.4(6)	-1.5(5)	-0.8(5)	-5.2(5)
C19	28.4(7)	25.3(6)	27.5(7)	-6.9(5)	2.4(6)	-6.8(5)
C20	30.3(8)	28.9(7)	35.0(8)	-7.5(6)	8.2(6)	-2.9(6)
C21	21.4(7)	30.9(7)	38.9(9)	-1.2(6)	3.4(6)	-5.4(6)
C22	24.9(7)	33.6(7)	30.8(7)	-2.8(6)	-3.6(6)	-10.8(6)
C23	23.5(7)	28.9(7)	25.5(7)	-5.4(5)	-0.4(5)	-7.8(5)
C24	18.7(6)	24.3(6)	26.7(7)	-7.1(5)	0.1(5)	-7.8(5)
C25	28.8(8)	27.8(7)	32.9(8)	-7.7(6)	-0.3(6)	-2.9(6)
C26	37.5(9)	34.2(8)	48.4(10)	-18.3(7)	10.8(8)	-4.6(7)
C27	41.9(10)	49.4(10)	38.6(9)	-24.0(8)	15.4(8)	-22.3(8)
C28	30.4(8)	52.5(10)	26.8(7)	-11.4(7)	1.4(6)	-19.7(7)
C29	21.4(7)	34.8(7)	27.7(7)	-5.3(6)	-1.5(6)	-6.9(6)
C30	23.8(7)	25.1(6)	23.6(6)	-7.0(5)	0.3(5)	-6.7(5)
C31	25.1(7)	26.7(6)	23.6(6)	-6.4(5)	1.5(5)	-4.6(5)
C32	35.9(8)	23.2(6)	24.3(7)	-7.1(5)	2.5(6)	-3.1(6)
C33	43.2(9)	26.3(7)	26.8(7)	-8.3(5)	7.0(6)	-15.5(6)
C34	30.0(8)	34.3(7)	38.1(8)	-11.0(6)	5.9(7)	-14.5(6)
C35	24.7(7)	27.3(7)	39.9(8)	-9.0(6)	2.1(6)	-5.4(6)
C36	23.9(7)	25.4(6)	25.6(7)	-9.9(5)	-1.5(5)	-4.8(5)
C37	47.8(11)	41.1(9)	41.8(10)	-3.2(7)	-15.6(8)	-20.0(8)
C38	56.5(13)	55.6(11)	46.9(11)	-8.3(9)	-27.0(9)	-17.7(10)
C39	52.7(12)	41.8(9)	30.8(8)	-5.3(7)	-15.2(8)	-0.9(8)
C40	46.7(10)	30.7(8)	36.2(9)	1.8(6)	-6.2(8)	-7.1(7)
C41	34.4(8)	28.3(7)	35.2(8)	-3.9(6)	-9.0(7)	-9.2(6)
B1	20.3(7)	21.8(6)	25.4(7)	-6.5(5)	-0.5(6)	-5.7(5)

Table S3. Anisotropic Displacement Parameters (Å^2×10^3) for [Mn(DPEA)(OAc)(MeOH)]BPh_4·MeOH (2). The Anisotropic displacement factor exponent takes the form: $-2\pi^2[h^2a^{*2}U_{11}+2hka^*b^*U_{12}+\ldots]$.

**Atom**	**U_{11}**	**U_{22}**	**U_{33}**	**U_{23}**	**U_{13}**	**U_{12}**
O5	24.4(7)	95.9(12)	64.0(10)	-48.3(9)	6.7(6)	-17.7(7)
C42	72.5(18)	133(3)	95(2)	-80(2)	40.4(16)	-59.5(18)

Table S4. Bond Lengths for [Mn(DPEA)(OAc)(MeOH)]BPh_4·MeOH (2).

Atom	Atom	Length/Å	Atom	Atom	Length/Å
Mn1	O1	2.3185(10)	C18	C19	1.4046(19)
Mn1	O2	2.1866(11)	C18	C23	1.4036(19)
Mn1	O3	2.2346(10)	C18	B1	1.646(2)
Mn1	O4	2.3670(11)	C19	C20	1.391(2)
Mn1	N1	2.2768(12)	C20	C21	1.388(2)
Mn1	N2	2.3528(12)	C21	C22	1.378(2)
Mn1	N3	2.3016(12)	C22	C23	1.397(2)
O1	C8	1.4310(18)	C24	C25	1.396(2)
O2	C17	1.430(2)	C24	C29	1.405(2)
O3	C15	1.2649(17)	C24	B1	1.643(2)
O4	C15	1.2596(17)	C26	C25	1.396(2)
N1	C1	1.3459(18)	C27	C26	1.381(3)
N1	C5	1.3437(18)	C28	C27	1.382(3)
N2	C6	1.4767(17)	C29	C28	1.389(2)
N2	C7	1.4801(18)	C30	C31	1.399(2)
N2	C9	1.4716(17)	C30	C35	1.406(2)
N3	C10	1.3411(18)	C30	B1	1.649(2)
N3	C14	1.343(2)	C32	C31	1.392(2)
C2	C1	1.380(2)	C33	C32	1.380(2)
C3	C2	1.379(2)	C34	C33	1.384(2)
C4	C3	1.378(2)	C35	C34	1.391(2)
C5	C4	1.387(2)	C36	C37	1.389(2)
C6	C5	1.5058(19)	C36	C41	1.399(2)
C8	C7	1.506(2)	C36	B1	1.649(2)
C9	C10	1.502(2)	C38	C37	1.392(3)
C10	C11	1.3887(19)	C39	C38	1.375(3)
C11	C12	1.380(2)	C40	C39	1.372(3)
C12	C13	1.377(3)	C41	C40	1.385(2)
C13	C14	1.382(2)	O5	C42	1.378(3)
C15	C16	1.495(2)			

Table S5. Bond Angles for [Mn(DPEA)(OAc)(MeOH)]BPh_4·MeOH (2).

Atom	Atom	Atom	Angle/˚	Atom	Atom	Atom	Angle/˚
O1	Mn1	O4	134.28(4)	N3	C10	C9	116.67(12)
O1	Mn1	N2	74.11(4)	N3	C10	C11	122.44(14)
O2	Mn1	O1	78.01(4)	C11	C10	C9	120.89(13)
O2	Mn1	O3	89.66(4)	C12	C11	C10	119.09(15)
O2	Mn1	O4	90.42(5)	C13	C12	C11	118.75(14)
O2	Mn1	N1	173.93(5)	C12	C13	C14	119.13(16)
O2	Mn1	N2	103.23(5)	N3	C14	C13	122.76(16)
O2	Mn1	N3	82.25(4)	O3	C15	C16	120.06(13)
O3	Mn1	O1	79.13(4)	O4	C15	O3	119.44(13)
O3	Mn1	O4	56.47(4)	O4	C15	C16	120.50(14)
O3	Mn1	N1	90.77(4)	C19	C18	B1	120.55(12)
O3	Mn1	N2	146.91(4)	C23	C18	C19	114.84(13)
O3	Mn1	N3	140.86(4)	C23	C18	B1	124.40(12)
N1	Mn1	O1	96.13(4)	C20	C19	C18	123.06(14)
N1	Mn1	O4	94.86(4)	C21	C20	C19	120.07(14)
N1	Mn1	N2	73.35(4)	C22	C21	C20	118.89(14)
N1	Mn1	N3	101.14(4)	C21	C22	C23	120.39(14)
N2	Mn1	O4	151.10(4)	C22	C23	C18	122.74(14)
N3	Mn1	O1	135.11(4)	C25	C24	C29	115.26(13)
N3	Mn1	O4	85.23(4)	C25	C24	B1	125.69(13)
N3	Mn1	N2	71.82(4)	C29	C24	B1	119.00(12)
C8	O1	Mn1	108.72(8)	C26	C25	C24	122.11(15)
C17	O2	Mn1	127.14(10)	C27	C26	C25	120.80(16)
C15	O3	Mn1	94.94(8)	C26	C27	C28	118.83(15)
C15	O4	Mn1	88.95(9)	C27	C28	C29	119.83(16)
C1	N1	Mn1	124.97(10)	C28	C29	C24	123.16(15)
C5	N1	Mn1	117.22(9)	C31	C30	C35	114.91(13)
C5	N1	C1	117.81(12)	C31	C30	B1	125.34(13)
C6	N2	Mn1	108.76(8)	C35	C30	B1	119.61(12)
C6	N2	C7	110.63(11)	C32	C31	C30	122.73(14)
C7	N2	Mn1	110.19(8)	C33	C32	C31	120.42(14)
C9	N2	Mn1	105.54(9)	C32	C33	C34	118.97(13)
C9	N2	C6	110.30(11)	C33	C34	C35	119.93(15)
C9	N2	C7	111.28(11)	C34	C35	C30	122.98(14)
C10	N3	Mn1	115.37(9)	C37	C36	C41	114.12(14)
C10	N3	C14	117.84(12)	C37	C36	B1	126.51(13)
C14	N3	Mn1	126.76(10)	C41	C36	B1	119.37(12)

Table S5. Bond Angles for [Mn(DPEA)(OAc)(MeOH)]BPh_4·MeOH (2).

Atom	Atom	Atom	Angle/˚	Atom	Atom	Atom	Angle/˚
N1	C1	C2	123.26(15)	C36	C37	C38	122.96(16)
C3	C2	C1	118.34(14)	C39	C38	C37	120.67(17)
C4	C3	C2	119.29(15)	C40	C39	C38	118.39(17)
C3	C4	C5	119.22(15)	C39	C40	C41	120.13(16)
N1	C5	C4	122.08(13)	C40	C41	C36	123.72(15)
N1	C5	C6	117.34(12)	C18	B1	C30	112.21(11)
C4	C5	C6	120.47(13)	C18	B1	C36	106.34(11)
N2	C6	C5	112.63(11)	C24	B1	C18	110.49(11)
N2	C7	C8	109.71(12)	C24	B1	C30	104.62(11)
O1	C8	C7	108.80(11)	C24	B1	C36	111.13(11)
N2	C9	C10	110.03(11)	C36	B1	C30	112.14(11)

Table S6. Hydrogen Atom Coordinates (Å×10^4) and Isotropic Displacement Parameters (Å^2×10^3) for [Mn(DPEA)(OAc)(MeOH)]BPh_4·MeOH (2).

Atom	*x*	*y*	*z*	U(eq)
H1	4995(9)	4913(15)	4011(12)	41
H2	8767(9)	4273(13)	3813(17)	55
H1a	5425.06	7043.67	5806.41	37
H2a	4132.84	8642.21	5833.68	47
H3	3613.38	9956.77	4226.94	47
H4	4404.01	9615.06	2644.36	37
H6a	5447.35	8249.65	1740.04	32
H6b	6619.82	8292.48	2196.81	32
H7a	5446.47	6386.11	1355.38	32
H7b	4730.31	6627.61	2367.41	32
H8a	5061.71	4725.49	2476.05	34
H8b	6391.82	4673.87	2410.07	34
H9a	7212.88	7048.74	1014.98	31
H9b	7555.49	5795.87	1701.84	31
H11	9070.62	7491.68	774.84	38
H12	10570.03	7903.5	1525.32	47
H13	10695.25	7467.42	3374.33	48
H14	9344.21	6640.76	4413.87	42
H16a	6989.45	5125.03	7792.32	56
H16b	8272.89	4617.93	7575.75	56
H16c	7325.85	3916.04	7611.4	56
H17a	7625.55	2947.61	3854.1	73
H17b	7317.6	3203.92	4979.99	73

Table S6. Hydrogen Atom Coordinates (Å×10^4) and Isotropic Displacement Parameters (Å^2×10^3) for [Mn(DPEA)(OAc)(MeOH)]BPh_4·MeOH (2).

**Atom**	***x***	***y***	***z***	**U(eq)**
H17c	8591.1	2721.61	4705.98	73
H19	5435.64	2675.39	2252.26	32
H20	3546.03	2667.53	2595.6	39
H21	2576.73	1812.05	1675.25	39
H22	3543.05	954.64	427.37	36
H23	5435.94	968.3	82.65	31
H25	8644.25	-145.43	827.73	36
H26	9348.96	-829.44	-607.43	47
H27	8662.78	52.26	-2311.19	47
H28	7262.68	1653.21	-2564.77	42
H29	6603.49	2364.45	-1141.4	34
H31	5772.78	3989.39	482.67	30
H32	6281.87	5686.64	-142.96	34
H33	8170.85	5801.79	-397.1	37
H34	9545.5	4179.15	-76.45	39
H35	9026.67	2481.03	483.23	37
H37	8770.18	1931.36	2274.63	51
H38	9534.25	818.81	3869.91	62
H39	8843.03	-744.18	4749.65	52
H40	7370.7	-1171.11	3991.52	48
H41	6616.43	-59.92	2401.22	39
H5	740(20)	4109(19)	3673(19)	58(7)
H42a	1096.34	3662.21	2246.26	129
H42b	-100.61	3314.16	2364.9	129
H42c	903.69	2591.74	3139.04	129

X-ray crystallographic tables for [Mn(DPMEA)(OAc)(MeOH)]BPh_4·2 MeOH (3)

Table S7. Crystal data and structure refinement for [Mn(DPMEA)(OAc)(MeOH)]BPh_4·2 MeOH (3).

Identification code	Tripodal 3 MNDPMEA
Empirical formula	$C_{44}H_{54}BMnN_3O_6$
Formula weight	786.65 g/mol
Temperature/K	173(2)
Crystal system	triclinic
Space group	$P\bar{1}$
a/Å	10.7350(6)
b/Å	12.0836(5)
c/Å	16.8786(6)
α/°	78.775(3)
β/°	88.865(4)
γ/°	74.451(5)
Volume/Å^3	2067.77(18)
Z	2
ρ_{calc}g/cm^3	1.263
μ/mm^{-1}	0.369
F(000)	834.0
Crystal size/mm^3	0.49 × 0.26 × 0.14
Radiation	Mo Kα (λ = 0.71073)
2Θ range for data collection/°	6.148 to 54.998
Index ranges	-12 ≤ h ≤ 13, -15 ≤ k ≤ 15, -21 ≤ l ≤ 20
Reflections collected	17923
Independent reflections	9464 [R_{int} = 0.0312, R_{sigma} = 0.0484]
Data/restraints/parameters	9464/0/517
Goodness-of-fit on F^2	1.080
Final R indexes [I>=2σ (I)]	R_1 = 0.0476, wR_2 = 0.1250
Final R indexes [all data]	R_1 = 0.0622, wR_2 = 0.1341
Largest diff. peak/hole / e Å^{-3}	0.85/-0.64

Table S8. Fractional Atomic Coordinates (×10^4) and Equivalent Isotropic Displacement Parameters ($Å^2$×10^3) for [Mn(DPMEA)(OAc)(MeOH)]BPh_4·2 MeOH (3). U_{eq} is defined as 1/3 of the trace of the orthogonalised U_{IJ} tensor.

**Atom**	**X**	**Y**	**z**	**U(eq)**
Mn1	3026.3(3)	4279.7(2)	1596.2(2)	24.36(10)
O1	1827.3(16)	3831.3(14)	645.5(9)	44.5(4)
O2	2692.6(15)	5276.1(13)	257.2(8)	37.0(3)
O3	4628.3(14)	2813.3(12)	1412.5(10)	37.1(3)
O4	4822.6(16)	5064.2(15)	1486.4(9)	46.9(4)
N1	1792.9(16)	5912.6(14)	1968.8(9)	29.4(3)
N2	2308.3(16)	2869.5(14)	2459.0(9)	28.8(3)
N3	3745.0(18)	4195.4(15)	2923.1(10)	35.7(4)
C1	2054.1(19)	4610.8(19)	97.2(12)	32.5(4)
C2	1532(3)	4755(2)	-750.9(13)	50.3(6)
C3	4556(3)	1710.9(19)	1253.2(16)	48.3(6)
C4	1035.6(19)	6834.9(18)	1462.2(13)	34.4(4)
C5	378(2)	7848(2)	1706.4(14)	41.2(5)
C6	507(2)	7942(2)	2499.7(15)	45.5(5)
C7	1292(2)	7011(2)	3022.4(13)	43.3(5)
C8	1911(2)	6002.3(18)	2744.5(12)	34.5(4)
C9	2676(3)	4927.6(19)	3311.8(12)	43.6(6)
C10	4020(2)	2971.4(19)	3349.7(12)	37.3(5)
C11	2992.5(19)	2407.6(17)	3162.9(11)	29.7(4)
C12	2813(2)	1431.3(19)	3679.0(13)	39.3(5)
C13	1935(2)	886(2)	3465.9(14)	43.7(5)
C14	1212(2)	1364.9(19)	2752.6(14)	39.7(5)
C15	1421.0(19)	2352.7(18)	2271.6(12)	31.4(4)
C16	4812(3)	6119(2)	960(2)	75.1(10)
C17	5005(4)	5491(4)	2226(3)	32.3(10)
C18	5086(10)	4398(7)	2858(7)	37(2)
C17A	5642(4)	4506(4)	2256(3)	41.0(9)
C18A	4793(8)	4788(6)	2930(6)	36.1(15)
C19	5525.4(16)	7645.8(14)	4165.8(10)	21.2(3)
C22	4987.8(18)	6691.5(15)	4375.5(11)	25.9(4)
C23	3927.2(18)	6710.6(16)	4861.4(11)	28.6(4)
C24	3353.6(18)	7698.6(16)	5172.2(11)	27.3(4)
C25	3864.4(18)	8651.6(16)	4995.4(11)	26.4(4)
C26	4927.4(17)	8619.5(15)	4505.7(11)	23.7(4)
C27	7476.2(17)	8580.1(16)	3622.8(11)	25.0(4)

C28	8488(2)	8269.4(19)	4203.2(12)	34.0(4)
C29	9183(2)	9044(2)	4332.1(14)	43.0(5)
C30	8896(2)	10164(2)	3885.0(14)	43.3(6)
C31	7898(2)	10517(2)	3311.5(14)	42.1(5)
C32	7202(2)	9732.7(18)	3189.0(12)	33.3(4)
C33	7635.7(17)	6329.9(15)	3571.6(11)	23.8(4)
C34	8040.7(19)	5629.2(17)	4336.9(12)	32.6(4)
C35	8907(2)	4523.6(18)	4446.0(14)	38.4(5)
C36	9412(2)	4056.1(18)	3787.9(15)	40.8(5)
C37	9035(2)	4719(2)	3021.6(15)	43.3(5)
C38	8170.0(19)	5828.6(17)	2921.1(12)	31.8(4)
C39	5894.4(18)	8118.7(15)	2590.2(11)	24.6(4)
C40	6572(2)	8329.7(16)	1875.5(12)	31.6(4)
C41	5968(2)	8720.9(17)	1114.5(12)	37.5(5)
C42	4640(2)	8955.3(17)	1031.3(13)	40.0(5)
C43	3933(2)	8786.0(18)	1714.7(13)	37.4(5)
C44	4547.3(19)	8372.2(16)	2476.3(12)	30.0(4)
B1	6638.5(19)	7660.1(17)	3487.2(12)	22.7(4)
O5	6941.2(18)	2916.8(16)	912.2(13)	65.0(5)
C45	7845(4)	1861(4)	846(3)	107.1(13)
O6	716(2)	2195.9(19)	176.2(14)	80.6(7)
C46	1277(3)	1016(3)	499(2)	66.0(8)

Table S9. Anisotropic Displacement Parameters (Å^2×10^3) for [Mn(DPMEA)(OAc)(MeOH)]BPh_4·2 MeOH (3). The Anisotropic displacement factor exponent takes the form: -$2\pi^2[h^2a^{*2}U_{11}+2hka^*b^*U_{12}+…]$.

**Atom**	**U11**	**U22**	**U33**	**U23**	**U13**	**U12**
Mn1	27.66(16)	26.62(16)	20.38(15)	-4.25(11)	0.07(11)	-10.20(11)
O1	50.8(10)	61.1(10)	25.6(7)	2.3(7)	-5.7(7)	-29.2(8)
O2	42.3(8)	42.1(8)	28.8(7)	-6.6(6)	3.8(6)	-15.8(7)
O3	38.2(8)	28.3(7)	49.4(9)	-16.1(6)	10.7(7)	-11.3(6)
O4	52.4(10)	68.3(11)	34.5(8)	-10.7(8)	1.5(7)	-40.6(9)
N1	30.5(8)	35.5(9)	23.5(8)	-6.8(7)	3.0(7)	-10.4(7)
N2	30.8(8)	32.6(8)	23.1(8)	-2.4(6)	2.1(7)	-11.0(7)
N3	43.0(10)	42.2(10)	25.2(8)	-0.1(7)	-6.6(8)	-21.4(8)
C1	26.7(10)	46.1(12)	24.9(10)	-6.8(8)	3.2(8)	-10.6(8)
C2	50.8(14)	77.8(18)	24.1(11)	-0.2(11)	-3.8(10)	-26.8(13)
C3	62.8(16)	31.6(11)	55.0(15)	-18.9(10)	5.1(12)	-13.0(10)
C4	31.4(10)	41.5(11)	28.8(10)	-7.6(9)	0.6(8)	-6.9(8)
C5	32.4(11)	41.6(12)	45.5(13)	-7.5(10)	5.0(10)	-3.8(9)
C6	48.9(14)	40.2(12)	49.7(14)	-18.2(11)	19.0(11)	-10.4(10)
C7	59.9(15)	46.9(13)	30.4(11)	-17.0(10)	11.8(10)	-20.8(11)
C8	45.0(12)	39.8(11)	24.6(9)	-9.1(8)	5.7(9)	-19.7(9)
C9	73.4(17)	39.9(12)	20.3(10)	-6.5(9)	-2.5(10)	-19.4(11)
C10	37.8(11)	43.6(12)	26.5(10)	4.1(9)	-8.0(9)	-11.6(9)
C11	28.1(10)	31.3(10)	25.2(9)	-0.5(8)	0.9(8)	-3.8(7)
C12	36.3(11)	38.2(11)	34.7(11)	6.9(9)	0.4(9)	-5.2(9)
C13	43.2(13)	37.6(12)	45.0(13)	6.9(10)	7.8(11)	-13.2(10)
C14	36.1(11)	44.2(12)	42.6(12)	-5.0(10)	9.8(10)	-20.5(9)
C15	28.7(10)	39.3(11)	27.3(10)	-4.3(8)	4.7(8)	-12.9(8)
C16	55.4(16)	33.4(13)	144(3)	-18.2(16)	32.8(19)	-26.7(12)
C17	33(2)	30(2)	40(3)	-11.9(19)	-4.7(19)	-16.1(18)
C18	41(5)	32(5)	39(4)	-4(4)	-18(4)	-11(4)
C17A	36(2)	51(3)	41(2)	-7.2(19)	-4.4(19)	-20.8(19)
C18A	42(4)	32(4)	39(3)	-8(3)	-11(3)	-15(3)
C19	22.0(8)	23.9(8)	18.4(8)	-3.9(6)	-0.6(7)	-7.5(7)
C22	29.3(9)	24.2(9)	26.7(9)	-9.3(7)	2.6(8)	-8.7(7)
C23	30.9(10)	28.3(9)	29.5(10)	-2.5(7)	0.4(8)	-15.1(8)
C24	22.8(9)	33.7(10)	24.5(9)	-2.4(7)	2.8(7)	-8.5(7)
C25	27.4(9)	25.0(9)	25.7(9)	-5.6(7)	4.1(7)	-5.0(7)
C26	26.8(9)	20.9(8)	23.6(9)	-1.9(7)	0.1(7)	-8.4(7)
C27	24.1(9)	31.9(9)	22.1(9)	-9.9(7)	7.8(7)	-10.3(7)

C28	31.7(10)	43.1(11)	30.4(10)	-12.9(9)	1.5(8)	-11.3(9)
C29	32.0(11)	66.5(16)	41.6(12)	-28.7(12)	4.0(10)	-19.2(10)
C30	40.4(12)	62.0(15)	48.4(13)	-34.6(12)	21.8(10)	-34.0(11)
C31	56.2(14)	36.8(11)	43.7(12)	-14.6(10)	17.2(11)	-26.3(10)
C32	37.2(11)	35.1(11)	32.4(10)	-10.1(8)	5.3(9)	-15.7(8)
C33	22.0(9)	26.0(9)	25.6(9)	-7.0(7)	1.3(7)	-8.8(7)
C34	31.5(10)	35.1(10)	28.2(10)	-4.4(8)	3.7(8)	-5.2(8)
C35	31.8(11)	34.6(11)	41.0(12)	4.6(9)	-1.6(9)	-4.0(8)
C36	31.6(11)	30.4(11)	55.1(14)	-8.6(10)	-3.1(10)	0.9(8)
C37	38.7(12)	43.8(13)	43.2(13)	-21.4(10)	-1.7(10)	5.4(9)
C38	31.0(10)	35.0(10)	28.1(10)	-10.2(8)	-1.0(8)	-3.6(8)
C39	30.9(10)	21.4(8)	22.6(9)	-6.5(7)	2.7(7)	-7.4(7)
C40	38.6(11)	29.2(10)	27.7(10)	-4.4(8)	3.9(8)	-11.2(8)
C41	60.4(15)	29.0(10)	23.3(10)	-3.4(8)	6.1(9)	-13.6(9)
C42	62.7(15)	27.5(10)	26.0(10)	-1.6(8)	-12.4(10)	-7.0(9)
C43	38.9(12)	31.6(10)	38.2(12)	-4.9(9)	-11.3(9)	-4.0(8)
C44	33.5(10)	29.1(10)	25.7(9)	-4.4(8)	-2.6(8)	-6.0(8)
B1	23.8(10)	23.9(9)	21.0(9)	-5.1(8)	3.9(8)	-7.0(7)
O5	48.7(10)	52.1(11)	79.9(14)	5.8(10)	27.8(10)	-4.5(8)
O6	86.0(16)	72.5(14)	82.7(15)	19.3(11)	-43.5(13)	-41.6(12)
C46	57.3(17)	68.1(19)	71(2)	-14.4(15)	0.5(15)	-13.6(14)

Table S10. Bond Lengths for [Mn(DPMEA)(OAc)(MeOH)]BPh_4·2 MeOH (3).

Atom	Atom	Length/Å	Atom	Atom	Length/Å
Mn1	O1	2.3064(14)	C17	C18	1.511(12)
Mn1	O2	2.3296(14)	C17A	C18A	1.478(10)
Mn1	O3	2.1800(14)	C19	C22	1.406(2)
Mn1	O4	2.3556(15)	C19	C26	1.403(2)
Mn1	N1	2.2564(16)	C19	B1	1.640(3)
Mn1	N2	2.3049(16)	C22	C23	1.388(3)
Mn1	N3	2.3578(16)	C23	C24	1.384(3)
O1	C1	1.256(3)	C24	C25	1.384(3)
O2	C1	1.256(2)	C25	C26	1.392(3)
O3	C3	1.431(2)	C27	C28	1.400(3)
O4	C16	1.402(3)	C27	C32	1.398(3)
O4	C17	1.475(4)	C27	B1	1.655(3)
O4	C17A	1.517(4)	C28	C29	1.393(3)
N1	C4	1.344(3)	C29	C30	1.372(3)
N1	C8	1.346(2)	C30	C31	1.379(3)
N2	C11	1.349(2)	C31	C32	1.400(3)
N2	C15	1.343(2)	C33	C34	1.405(3)
N3	C9	1.477(3)	C33	C38	1.396(3)
N3	C10	1.467(3)	C33	B1	1.657(3)
N3	C18	1.523(12)	C34	C35	1.389(3)
N3	C18A	1.488(9)	C35	C36	1.379(3)
C1	C2	1.508(3)	C36	C37	1.386(3)
C4	C5	1.374(3)	C37	C38	1.393(3)
C5	C6	1.378(3)	C39	C40	1.412(3)
C6	C7	1.375(3)	C39	C44	1.404(3)
C7	C8	1.386(3)	C39	B1	1.650(3)
C8	C9	1.501(3)	C40	C41	1.388(3)
C10	C11	1.510(3)	C41	C42	1.382(3)
C11	C12	1.379(3)	C42	C43	1.380(3)
C12	C13	1.375(3)	C43	C44	1.394(3)
C13	C14	1.383(3)	O5	C45	1.403(4)
C14	C15	1.376(3)	O6	C46	1.393(3)

Table S11. Bond Angles for [Mn(DPMEA)(OAc)(MeOH)]BPh_4·2 MeOH (3).

Atom	Atom	Atom	Angle/˚	Atom	Atom	Atom	Angle/˚
O1	Mn1	O2	56.02(5)	C7	C8	C9	121.13(18)
O1	Mn1	O4	132.05(5)	N3	C9	C8	113.40(17)
O1	Mn1	N3	154.17(6)	N3	C10	C11	111.60(15)
O2	Mn1	O4	81.04(5)	N2	C11	C10	117.09(17)
O2	Mn1	N3	148.49(5)	N2	C11	C12	122.13(18)
O3	Mn1	O1	88.35(6)	C12	C11	C10	120.72(17)
O3	Mn1	O2	97.83(6)	C13	C12	C11	119.47(19)
O3	Mn1	O4	76.45(6)	C12	C13	C14	118.8(2)
O3	Mn1	N1	164.33(6)	C15	C14	C13	118.79(19)
O3	Mn1	N2	84.54(6)	N2	C15	C14	122.95(18)
O3	Mn1	N3	93.29(6)	O4	C17	C18	100.4(4)
O4	Mn1	N3	73.05(6)	C17	C18	N3	107.9(6)
N1	Mn1	O1	106.97(6)	C18A	C17A	O4	106.2(4)
N1	Mn1	O2	88.41(5)	C17A	C18A	N3	107.4(5)
N1	Mn1	O4	90.45(6)	C22	C19	B1	121.56(15)
N1	Mn1	N2	100.25(6)	C26	C19	C22	114.74(16)
N1	Mn1	N3	74.42(6)	C26	C19	B1	123.27(15)
N2	Mn1	O1	83.04(5)	C23	C22	C19	123.22(16)
N2	Mn1	O2	138.75(5)	C24	C23	C22	120.05(17)
N2	Mn1	O4	138.39(6)	C25	C24	C23	118.83(18)
N2	Mn1	N3	71.49(6)	C24	C25	C26	120.43(17)
C1	O1	Mn1	92.38(12)	C25	C26	C19	122.72(16)
C1	O2	Mn1	91.31(12)	C28	C27	B1	122.18(16)
C3	O3	Mn1	127.45(14)	C32	C27	C28	115.00(17)
C16	O4	Mn1	122.25(16)	C32	C27	B1	122.79(16)
C16	O4	C17	94.7(2)	C29	C28	C27	122.6(2)
C16	O4	C17A	127.6(2)	C30	C29	C28	120.6(2)
C17	O4	Mn1	110.4(2)	C29	C30	C31	119.04(18)
C17A	O4	Mn1	106.30(18)	C30	C31	C32	119.9(2)
C4	N1	Mn1	124.85(13)	C27	C32	C31	122.8(2)
C4	N1	C8	117.86(17)	C34	C33	B1	120.50(16)
C8	N1	Mn1	117.06(13)	C38	C33	C34	114.81(17)
C11	N2	Mn1	115.65(12)	C38	C33	B1	124.66(16)
C15	N2	Mn1	125.71(12)	C35	C34	C33	123.11(19)
C15	N2	C11	117.77(17)	C36	C35	C34	120.4(2)
C9	N3	Mn1	107.37(12)	C35	C36	C37	118.32(19)
C9	N3	C18	122.5(3)	C36	C37	C38	120.7(2)

C9	N3	C18A	103.1(3)	C37	C38	C33	122.68(19)
C10	N3	Mn1	107.54(12)	C40	C39	B1	121.93(16)
C10	N3	C9	109.61(17)	C44	C39	C40	114.76(17)
C10	N3	C18	102.5(4)	C44	C39	B1	123.26(17)
C10	N3	C18A	117.1(3)	C41	C40	C39	123.0(2)
C18	N3	Mn1	106.6(4)	C42	C41	C40	120.1(2)
C18A	N3	Mn1	111.8(4)	C43	C42	C41	118.89(18)
O1	C1	C2	119.47(19)	C42	C43	C44	120.7(2)
O2	C1	O1	120.20(18)	C43	C44	C39	122.4(2)
O2	C1	C2	120.33(19)	C19	B1	C27	110.03(13)
N1	C4	C5	122.76(19)	C19	B1	C33	109.71(14)
C4	C5	C6	119.3(2)	C19	B1	C39	107.57(14)
C7	C6	C5	118.5(2)	C27	B1	C33	109.01(14)
C6	C7	C8	119.5(2)	C39	B1	C27	108.95(14)
N1	C8	C7	122.0(2)	C39	B1	C33	111.55(14)
N1	C8	C9	116.77(18)				

Table S12. Hydrogen Atom Coordinates (Å×10^4) and Isotropic Displacement Parameters (Å^2×10^3) for [Mn(DPMEA)(OAc)(MeOH)]BPh_4·2 MeOH (3).

**Atom**	***x***	***y***	***z***	**U(eq)**
H3	5299.93	2967.3	1213.24	56
H2A	615.99	5189.97	-790.29	76
H2B	1625.01	3981.53	-880.7	76
H2C	2017.53	5187.01	-1132.64	76
H3A	4345.53	1796.82	678.25	72
H3B	3881.46	1452.4	1577.64	72
H3C	5390.79	1128.69	1394.66	72
H4	951	6784.58	912.09	41
H5	-159.59	8477.57	1332.32	49
H6	63.97	8635.28	2681.15	55
H7	1408.86	7057.21	3570.18	52
H9A	3037.11	5169.58	3764.77	52
H9B	2086.12	4451.24	3540.66	52
H10A	4068.64	2939.32	3939.21	45
H10B	4870.98	2526.08	3189.52	45
H12	3293.02	1137.78	4177.72	47
H13	1826.93	193.18	3802.81	52
H14	582.65	1018.1	2597.41	48
H15	915.11	2684.18	1785.18	38
H16A	4856.45	5997.75	401.78	113
H16B	5558.74	6380.46	1089.69	113
H16C	4012.65	6715.53	1021.43	113
H17A	4260.64	6144.85	2307.08	39
H17B	5810.93	5742.52	2218.46	39
H18A	5380.25	4498.08	3385.89	45
H18B	5712.8	3718.14	2700.84	45
H17C	5958.64	3647.34	2299.34	49
H17D	6397.43	4828.88	2261.05	49
H18C	4420.61	5645.35	2859.35	43
H18D	5292.35	4504.32	3450.72	43
H22	5369.52	5999.83	4174.71	31
H23	3594.55	6045.32	4980.77	34
H24	2622.21	7721.74	5501.18	33
H25	3487.29	9332.35	5209.52	32
H26	5261.75	9284.36	4397.18	28
H28	8709.4	7499.68	4521.7	41

H29	9862.54	8795.32	4732.95	52
H30	9376.72	10688.74	3969.44	52
H31	7683.01	11290.96	2999.79	51
H32	6513.42	9995.28	2794.16	40
H34	7706	5925.64	4801.87	39
H35	9154	4086.67	4977.07	46
H36	10002.07	3299.02	3858.12	49
H37	9371.16	4412.81	2560.28	52
H38	7933.63	6262.15	2388.07	38
H40	7483.42	8197.85	1917.59	38
H41	6469.2	8827.76	649.64	45
H42	4219.79	9228.46	512.42	48
H43	3018.13	8953.24	1665.67	45
H44	4035.93	8257.47	2935.11	36
H5A	7083.38	3465.64	565.3	98
H45A	7822.82	1738.68	289.67	161
H45B	7630.07	1210.35	1215.31	161
H45C	8713.14	1895.79	986.36	161
H6A	1154.97	2603.19	325.54	121
H46A	1505.22	583.66	58.53	99
H46B	662.26	698.49	845.88	99
H46C	2059.83	937.69	818.02	99

Table S13. Atomic Occupancy for [Mn(DPMEA)(OAc)(MeOH)]BPh_4·2 MeOH (3).

Atom	*Occupancy*	Atom	*Occupancy*	Atom	*Occupancy*
C17	0.45	H17A	0.45	H17B	0.45
C18	0.45	H18A	0.45	H18B	0.45
C17A	0.55	H17C	0.55	H17D	0.55
C18A	0.55	H18C	0.55	H18D	0.55

X-ray crystallographic tables for [Mn(PDEA)(OAc)(MeOH)]BPh_4 (4)

Table S14. Crystal data and structure refinement for [Mn(PDEA)(OAc)(MeOH)]BPh_4 (**4**).

Identification code	Tripodal 4 MnPDEA
Empirical formula	$C_{37}H_{43}BMnN_2O_5$
Formula weight	661.48 g/mol
Temperature/K	299.2(2)
Crystal system	triclinic
Space group	$P\bar{1}$
a/Å	10.63990(10)
b/Å	11.7269(2)
c/Å	15.7331(3)
α/°	111.191(2)
β/°	95.4590(10)
γ/°	90.3140(10)
Volume/Å^3	1820.34(5)
Z	2
ρ_{calc}g/cm^3	1.207
μ/mm^{-1}	3.281
F(000)	698.0
Crystal size/mm^3	0.298 × 0.238 × 0.116
Radiation	Cu Kα (λ = 1.54184)
2Θ range for data collection/°	6.058 to 137
Index ranges	-12 ≤ h ≤ 10, -14 ≤ k ≤ 14, -18 ≤ l ≤ 18
Reflections collected	32924
Independent reflections	6666 [R_{int} = 0.0573, R_{sigma} = 0.0418]
Data/restraints/parameters	6666/0/420
Goodness-of-fit on F^2	1.081
Final R indexes [I>=2σ (I)]	R_1 = 0.0481, wR_2 = 0.1151
Final R indexes [all data]	R_1 = 0.0555, wR_2 = 0.1197
Largest diff. peak/hole / e Å^{-3}	0.36/-0.18

Table S15. Fractional Atomic Coordinates (×10^4) and Equivalent Isotropic Displacement Parameters (Å^2×10^3) for [Mn(PDEA)(OAc)(MeOH)]BPh_4 (**4**). U_{eq} is defined as 1/3 of the trace of the orthogonalised U_{IJ} tensor.

**Atom**	**X**	**Y**	**z**	**U(eq)**
Mn1	2821.4(3)	136.0(3)	5992.4(3)	45.48(13)
O1	1322.0(17)	-1341.6(17)	5228.5(15)	61.1(5)
O2	4781.0(17)	-577(2)	5993.4(16)	69.6(6)
O3	3042.1(15)	237.6(17)	4659.9(12)	54.3(4)
O4	1236.8(16)	991.8(16)	5124.4(12)	55.9(4)
O5	3845(2)	1985(2)	6604.1(14)	73.5(6)
N1	2902.8(19)	-1318.1(19)	6754.6(16)	53.8(5)
N2	1876.8(19)	945.2(19)	7271.7(15)	51.4(5)
C1	1426(3)	-2448(3)	5411(2)	68.1(8)
C2	1766(3)	-2149(3)	6419(2)	63.2(7)
C3	4054(3)	-2025(3)	6570(3)	71.6(9)
C4	5160(3)	-1222(3)	6573(2)	69.3(8)
C5	2903(3)	-600(3)	7731(2)	67.0(8)
C6	2002(2)	424(3)	7906.8(18)	54.4(6)
C7	1385(3)	852(3)	8692(2)	67.0(8)
C8	668(3)	1858(3)	8839(2)	71.4(8)
C9	569(3)	2423(3)	8212(2)	68.2(8)
C10	1172(3)	1933(3)	7434(2)	58.9(7)
C11	1960(2)	633(2)	4505.3(18)	48.3(6)
C12	1559(3)	656(3)	3579(2)	66.0(8)
C13	4623(4)	2559(3)	6191(3)	86.4(10)
C14	8162(2)	5908(2)	6868.0(17)	48.5(6)
C15	6871(3)	5757(3)	6617(2)	79.3(10)
C16	6337(3)	5585(4)	5756(2)	95.3(12)
C17	7073(4)	5542(3)	5073(2)	79.3(9)
C18	8351(4)	5707(3)	5285(2)	72.7(9)
C19	8877(3)	5882(2)	6170.7(18)	59.4(7)
C20	8254(2)	5016(2)	8235.5(18)	50.2(6)
C21	7772(3)	3879(3)	7627(2)	66.2(7)
C22	7451(3)	2930(3)	7930(3)	84.7(11)

C23	7610(3)	3121(4)	8851(3)	85.5(11)
C24	8101(3)	4213(3)	9459(3)	80.0(10)
C25	8414(3)	5127(3)	9152(2)	66.0(7)
C26	8032(2)	7428(2)	8507.3(17)	50.8(6)
C27	6920(3)	7446(3)	8911(2)	65.3(7)
C28	6248(3)	8495(3)	9258(2)	83.8(10)
C29	6681(4)	9580(3)	9230(3)	87.3(11)
C30	7787(4)	9614(3)	8854(2)	80.0(9)
C31	8436(3)	8557(2)	8495(2)	62.5(7)
C32	10247(2)	6218(2)	8080.6(18)	50.1(6)
C33	11010(3)	7167(3)	8727.3(19)	58.4(7)
C34	12321(3)	7134(3)	8821(2)	74.4(9)
C35	12920(3)	6151(4)	8276(3)	84.3(11)
C36	12207(3)	5191(3)	7641(3)	79.4(9)
C37	10909(3)	5227(3)	7551(2)	64.7(7)
B1	8696(3)	6148(3)	7931.9(19)	47.1(6)

Table S16. Anisotropic Displacement Parameters ($Å^2 \times 10^3$) for [Mn(PDEA)(OAc)(MeOH)]BPh_4 (**4**). The Anisotropic displacement factor exponent takes the form: $-2\pi^2[h^2a^{*2}U_{11}+2hka^*b^*U_{12}+\ldots]$.

**Atom**	**U11**	**U22**	**U33**	**U23**	**U13**	**U12**
Mn1	43.4(2)	48.1(2)	53.7(2)	26.12(18)	16.65(17)	3.52(16)
O1	49.2(10)	57.4(11)	86.1(14)	36.8(10)	9.1(10)	-1.9(8)
O2	45.4(10)	90.3(14)	102.5(16)	65.2(13)	28.0(10)	15.1(10)
O3	39.4(9)	72.0(11)	63.8(11)	38.3(9)	9.8(8)	1.2(8)
O4	51.3(10)	61.9(11)	60.7(11)	27.3(9)	15.8(9)	2.3(8)
O5	80.7(14)	70.7(13)	67.5(13)	21.3(11)	15.8(11)	-20.0(11)
N1	42.3(11)	55.8(12)	76.9(15)	37.4(12)	18.0(10)	7.6(9)
N2	50.4(12)	55.2(12)	57.0(13)	27.5(10)	17.7(10)	6.5(9)
C1	62.8(17)	50.2(15)	96(2)	30.8(16)	12.8(16)	-4.0(13)
C2	51.9(15)	56.4(15)	97(2)	44.4(16)	19.1(15)	2.0(12)
C3	51.0(16)	72.3(19)	116(3)	59.9(19)	24.9(16)	15.8(14)
C4	42.5(14)	89(2)	100(2)	59.4(19)	18.8(15)	13.9(14)
C5	62.7(17)	79(2)	78(2)	49.9(17)	10.4(15)	8.3(15)
C6	51.3(14)	63.3(16)	55.8(15)	29.1(13)	11.3(12)	-2.5(12)
C7	65.1(18)	87(2)	54.0(16)	30.4(15)	11.9(14)	-6.8(16)
C8	64.5(18)	85(2)	55.6(17)	10.2(16)	23.8(14)	-5.0(16)

C9	62.3(18)	64.2(17)	74(2)	15.7(15)	25.0(15)	9.1(14)
C10	57.8(16)	58.4(16)	65.5(17)	24.6(13)	20.7(13)	10.5(13)
C11	42.6(13)	50.3(13)	58.7(15)	26.6(12)	10.2(11)	-2.7(10)
C12	52.0(16)	92(2)	60.9(17)	36.6(16)	5.6(13)	6.2(14)
C13	96(3)	79(2)	88(2)	33.9(19)	12(2)	-29.9(19)
C14	54.3(14)	40.8(12)	49.0(14)	13.5(11)	10.2(11)	4.7(10)
C15	59.8(18)	115(3)	54.0(18)	19.4(18)	8.4(14)	15.1(18)
C16	68(2)	132(3)	68(2)	16(2)	-1.5(17)	25(2)
C17	100(3)	77(2)	55.3(18)	20.9(16)	-7.2(18)	12.2(19)
C18	99(3)	68.1(19)	51.8(17)	21.8(14)	13.0(16)	-12.7(17)
C19	66.4(17)	59.6(16)	53.2(16)	21.7(13)	7.5(13)	-12.8(13)
C20	46.5(13)	48.4(13)	59.2(15)	20.9(12)	16.4(11)	4.8(11)
C21	59.6(17)	58.5(17)	83(2)	28.3(15)	9.4(15)	-3.7(13)
C22	62.1(19)	56.2(18)	139(4)	38(2)	14(2)	-6.7(14)
C23	65(2)	91(3)	136(3)	77(3)	34(2)	12.8(18)
C24	83(2)	90(2)	96(3)	60(2)	39(2)	23.8(19)
C25	78(2)	65.1(17)	64.4(18)	31.3(15)	21.8(15)	8.8(15)
C26	57.5(15)	49.3(14)	43.1(13)	13.5(11)	5.7(11)	3.5(11)
C27	63.2(17)	60.2(17)	68.1(18)	14.9(14)	19.7(14)	9.5(13)
C28	69(2)	86(2)	83(2)	12.4(19)	20.3(17)	18.0(18)
C29	95(3)	65(2)	83(2)	4.4(18)	7(2)	29.8(19)
C30	100(3)	51.0(17)	83(2)	18.2(16)	4(2)	11.8(16)
C31	74.1(19)	52.4(15)	60.9(17)	20.1(13)	8.9(14)	5.4(13)
C32	52.7(14)	53.2(14)	53.0(14)	29.0(12)	9.1(11)	-0.5(11)
C33	64.4(17)	65.6(17)	52.9(15)	31.5(13)	3.5(13)	-8.1(13)
C34	66(2)	92(2)	80(2)	53(2)	-9.8(16)	-23.5(18)
C35	50.8(17)	112(3)	118(3)	76(3)	5.2(19)	-2.5(19)
C36	60.2(19)	85(2)	110(3)	50(2)	27.0(19)	20.2(17)
C37	56.7(16)	60.1(16)	81(2)	29.2(15)	13.3(14)	4.4(13)
B1	50.2(16)	45.1(14)	46.0(15)	15.2(12)	10.2(12)	-0.8(12)

Table S17. Bond Lengths for [Mn(PDEA)(OAc)(MeOH)]BPh_4 (**4**).

Atom	Atom	Length/Å	Atom	Atom	Length/Å
Mn1	O1	2.2565(19)	C14	B1	1.637(4)
Mn1	O2	2.2506(18)	C15	C16	1.361(5)
Mn1	O3	2.1767(17)	C16	C17	1.375(5)
Mn1	O4	2.5072(18)	C17	C18	1.364(5)
Mn1	O5	2.254(2)	C18	C19	1.393(4)
Mn1	N1	2.411(2)	C20	C21	1.390(4)
Mn1	N2	2.231(2)	C20	C25	1.394(4)
O1	C1	1.429(3)	C20	B1	1.645(4)
O2	C4	1.414(3)	C21	C22	1.411(4)
O3	C11	1.278(3)	C22	C23	1.379(5)
O4	C11	1.252(3)	C23	C24	1.357(5)
O5	C13	1.405(4)	C24	C25	1.376(4)
N1	C2	1.478(3)	C26	C27	1.392(4)
N1	C3	1.475(3)	C26	C31	1.397(4)
N1	C5	1.461(4)	C26	B1	1.651(4)
N2	C6	1.344(3)	C27	C28	1.386(4)
N2	C10	1.342(3)	C28	C29	1.368(5)
C1	C2	1.503(4)	C29	C30	1.373(5)
C3	C4	1.502(4)	C30	C31	1.382(4)
C5	C6	1.504(4)	C32	C33	1.396(4)
C6	C7	1.385(4)	C32	C37	1.400(4)
C7	C8	1.368(5)	C32	B1	1.642(4)
C8	C9	1.371(5)	C33	C34	1.390(4)
C9	C10	1.373(4)	C34	C35	1.367(5)
C11	C12	1.489(4)	C35	C36	1.368(5)
C14	C15	1.385(4)	C36	C37	1.377(4)
C14	C19	1.385(4)			

Table S18. Bond Angles for [Mn(PDEA)(OAc)(MeOH)]BPh_4 (**4**).

Atom	Atom	Atom	Angle/˚	Atom	Atom	Atom	Angle/˚
O1	Mn1	O4	71.82(6)	C7	C8	C9	119.8(3)
O1	Mn1	N1	73.30(7)	C8	C9	C10	118.3(3)
O2	Mn1	O1	113.08(8)	N2	C10	C9	123.0(3)
O2	Mn1	O4	143.19(6)	O3	C11	C12	119.5(2)
O2	Mn1	O5	83.90(9)	O4	C11	O3	120.5(2)
O2	Mn1	N1	71.15(6)	O4	C11	C12	120.1(2)
O3	Mn1	O1	85.46(7)	C15	C14	B1	119.1(2)
O3	Mn1	O2	88.03(7)	C19	C14	C15	114.5(3)
O3	Mn1	O4	55.39(6)	C19	C14	B1	126.4(2)
O3	Mn1	O5	86.61(7)	C16	C15	C14	123.3(3)
O3	Mn1	N1	140.47(8)	C15	C16	C17	120.9(3)
O3	Mn1	N2	144.56(7)	C18	C17	C16	118.3(3)
O5	Mn1	O1	160.94(8)	C17	C18	C19	119.8(3)
O5	Mn1	O4	89.43(8)	C14	C19	C18	123.1(3)
O5	Mn1	N1	122.48(8)	C21	C20	C25	114.8(3)
N1	Mn1	O4	139.32(6)	C21	C20	B1	124.6(2)
N2	Mn1	O1	96.82(8)	C25	C20	B1	120.5(2)
N2	Mn1	O2	122.43(8)	C20	C21	C22	121.8(3)
N2	Mn1	O4	91.61(7)	C23	C22	C21	119.9(3)
N2	Mn1	O5	79.90(8)	C24	C23	C22	119.6(3)
N2	Mn1	N1	72.40(7)	C23	C24	C25	119.8(3)
C1	O1	Mn1	115.52(17)	C24	C25	C20	124.1(3)
C4	O2	Mn1	119.79(15)	C27	C26	C31	114.8(3)
C11	O3	Mn1	98.93(14)	C27	C26	B1	122.8(2)
C11	O4	Mn1	84.36(14)	C31	C26	B1	121.6(2)
C13	O5	Mn1	128.9(2)	C28	C27	C26	122.8(3)
C2	N1	Mn1	108.86(16)	C29	C28	C27	120.3(3)
C3	N1	Mn1	110.14(15)	C28	C29	C30	119.1(3)
C3	N1	C2	110.2(2)	C29	C30	C31	120.1(3)
C5	N1	Mn1	106.10(15)	C30	C31	C26	122.9(3)
C5	N1	C2	110.2(2)	C33	C32	C37	114.5(3)
C5	N1	C3	111.2(2)	C33	C32	B1	126.3(2)
C6	N2	Mn1	119.26(17)	C37	C32	B1	119.1(2)
C10	N2	Mn1	122.62(17)	C34	C33	C32	122.4(3)
C10	N2	C6	118.1(2)	C35	C34	C33	120.7(3)
O1	C1	C2	109.7(2)	C34	C35	C36	118.8(3)
N1	C2	C1	110.7(2)	C35	C36	C37	120.3(3)

N1	C3	C4	110.9(2)	C36	C37	C32	123.3(3)
O2	C4	C3	107.5(2)	C14	B1	C20	111.1(2)
N1	C5	C6	112.2(2)	C14	B1	C26	102.7(2)
N2	C6	C5	115.7(2)	C14	B1	C32	111.9(2)
N2	C6	C7	121.6(3)	C20	B1	C26	111.2(2)
C7	C6	C5	122.7(3)	C32	B1	C20	105.6(2)
C8	C7	C6	119.1(3)	C32	B1	C26	114.5(2)

Table S19. Hydrogen Atom Coordinates (Å×10^4) and Isotropic Displacement Parameters (Å^2×10^3) for [Mn(PDEA)(OAc)(MeOH)]BPh_4 (**4**).

**Atom**	**x**	**y**	**z**	**U(eq)**
H1	575.28	-1230.73	5112.05	92
H2	5407.25	-346.67	5823.46	104
H5	4002.39	2288.96	7162.68	110
H1A	2070.85	-2937.97	5069.68	82
H1B	628.96	-2918.67	5214.89	82
H2A	1064.36	-1764.21	6750.87	76
H2B	1923.29	-2898.64	6532.02	76
H3A	3911.23	-2686.13	5977.86	86
H3B	4239.61	-2382.78	7032.76	86
H4A	5412.39	-652.89	7190.62	83
H4B	5872.5	-1716.59	6351.17	83
H5A	3749.31	-261	7983	80
H5B	2668.48	-1133.93	8044.56	80
H7	1455.81	461.18	9112.46	80
H8	250.01	2157.6	9363.8	86
H9	104.69	3120.86	8310.18	82
H10	1088.85	2302.1	6999.42	71
H12A	706.36	329.71	3388.18	99
H12B	2108.04	169.57	3150.56	99
H12C	1605.37	1484.52	3601.59	99
H13A	4167.75	2603.17	5647.2	130
H13B	5362.51	2095.54	6031.23	130
H13C	4869.28	3370.84	6611.08	130
H15	6341.56	5774.89	7058.66	95
H16	5463.04	5495.35	5627.4	114
H17	6709.2	5403.54	4481.09	95
H18	8868.81	5702.31	4840.14	87
H19	9750.82	5986.82	6299.98	71
H21	7658.39	3740.3	7003.62	79
H22	7132.76	2177.47	7507.84	102
H23	7383.12	2504.18	9055.36	103
H24	8224.95	4344.47	10081.07	96
H25	8753.32	5864.82	9583.31	79
H27	6614.89	6723.21	8949.32	78
H28	5499.48	8460.92	9510.79	101
H29	6231.61	10285.32	9462.58	105

H30	8099.82	10349.02	8841.3	96
H31	9174.28	8598.49	8233.14	75
H33	10628.12	7846.77	9109.1	70
H34	12794.44	7787.48	9259.5	89
H35	13797.06	6134.34	8335.4	101
H36	12600.49	4512.64	7269.39	95
H37	10448.87	4559.73	7116.49	78

Table S20. Solvent masks information for [Mn(PDEA)(OAc)(MeOH)]BPh_4 (**4**).

**Number**	***x***	***y***	***z***	**Volume**	**Electron count**	**Content**
1	0.5	0.5	1	160.9	35.5	2 methanol

X-ray crystallographic tables for [Mn(PDMEA)(OAc)(MeOH)]BPh_4 (5)

Table S21. Crystal data and structure refinement for [Mn(PDMEA)(OAc)(MeOH)]BPh_4 (**5**).

Identification code	Tripodal 5 MnPDMEA
Empirical formula	$C_{39}H_{47}BMnN_2O_5$
Formula weight	689.53 g/mol
Temperature/K	173(2)
Crystal system	triclinic
Space group	P$\bar{1}$
a/Å	11.3885(5)
b/Å	12.0841(5)
c/Å	14.7728(7)
α/°	66.057(4)
β/°	82.169(4)
γ/°	71.989(4)
Volume/Å^3	1766.90(16)
Z	2
ρ_{calc}g/cm^3	1.296
μ/mm^{-1}	0.420
F(000)	730.0
Crystal size/mm^3	0.32 × 0.28 × 0.19
Radiation	Mo Kα (λ = 0.71073)
2Θ range for data collection/°	4.562 to 65.672
Index ranges	-17 ≤ h ≤ 17, -18 ≤ k ≤ 17, -22 ≤ l ≤ 22
Reflections collected	20687
Independent reflections	11634 [R_{int} = 0.0278, R_{sigma} = 0.0437]
Data/restraints/parameters	11634/3/440
Goodness-of-fit on F^2	1.026
Final R indexes [I>=2σ (I)]	R_1 = 0.0431, wR_2 = 0.1057
Final R indexes [all data]	R_1 = 0.0612, wR_2 = 0.1196
Largest diff. peak/hole / e Å^{-3}	0.42/-0.34

Table S22. Fractional Atomic Coordinates (×10^4) and Equivalent Isotropic Displacement Parameters (Å^2×10^3) for [Mn(PDMEA)(OAc)(MeOH)]BPh_4 (**5**). U_{eq} is defined as 1/3 of the trace of the orthogonalised U_{IJ} tensor.

**Atom**	**X**	**Y**	**z**	**U(eq)**
Mn1	6996.6(2)	284.8(2)	3674.0(2)	22.66(6)
O1	5383.1(9)	-87.2(10)	3383.8(8)	28.2(2)
O2	6416.6(9)	-247.8(9)	5348.8(8)	28.0(2)
O3	7915.1(10)	-1515.3(10)	4850.9(8)	31.8(2)
O4	8069.9(10)	-808.7(10)	2684.9(7)	30.9(2)
O5	5514.9(9)	2207.1(9)	3604.8(7)	25.7(2)
N1	8272.9(11)	1350.6(13)	3727.5(10)	30.9(3)
N2	7047.0(11)	1738.9(12)	2061.3(9)	27.8(2)
C1	5559.0(14)	3246.7(13)	2699.7(11)	29.5(3)
C2	5864.8(14)	2747.3(14)	1890.7(11)	31.9(3)
C3	7143.7(16)	1107.9(15)	1370.7(11)	34.7(3)
C4	8171.6(16)	-106.6(16)	1650.7(11)	38.3(4)
C5	8106.3(16)	2253.5(17)	1939.0(12)	38.4(4)
C6	8464.9(13)	2259.5(14)	2876.4(12)	31.7(3)
C7	9023.3(15)	3149.5(17)	2854.2(16)	42.6(4)
C8	9379.1(16)	3091(2)	3724.6(18)	49.7(5)
C9	9179.8(16)	2157(2)	4607.2(16)	48.5(5)
C10	8626.7(15)	1311.7(18)	4574.7(14)	39.8(4)
C11	7229.9(12)	-1300.4(13)	5534.2(10)	23.7(2)
C12	7380.3(14)	-2281.0(14)	6570.7(10)	29.9(3)
C13	8927.9(15)	-2038.7(15)	2986.2(12)	35.7(3)
C14	5248.9(16)	-961.9(17)	3009.3(13)	35.4(3)
C15	5158.4(16)	2597.2(15)	4417.9(12)	36.6(3)
C16	2779.3(12)	6206.8(13)	3209.8(10)	24.6(3)
C17	3442.5(16)	6133.4(16)	3975.8(11)	35.8(3)
C18	3293.1(16)	5406.4(17)	4967.5(12)	37.6(3)
C19	2438.6(16)	4729.2(15)	5252.4(11)	34.4(3)
C20	1726.0(16)	4809.7(16)	4533.5(12)	36.2(3)
C21	1897.2(14)	5532.3(15)	3540.9(11)	30.5(3)
C22	2022.9(12)	6933.3(13)	1365.1(10)	24.8(3)

C23	2094.3(15)	5768.6(15)	1330.4(11)	31.8(3)
C24	1241.3(16)	5606.3(18)	842.3(13)	39.0(4)
C25	264.6(15)	6627(2)	353.4(13)	40.4(4)
C26	162.3(14)	7795.2(18)	355.6(12)	36.8(4)
C27	1028.9(13)	7937.4(15)	854.5(11)	28.5(3)
C28	2732.9(13)	8544.7(13)	1855.4(10)	26.0(3)
C29	3232.0(16)	9372.6(14)	1039.7(12)	33.7(3)
C30	3004.1(19)	10645.0(16)	858.2(14)	44.4(4)
C31	2247.6(19)	11136.6(16)	1497.5(15)	48.2(5)
C32	1729.6(17)	10362.1(17)	2304.5(15)	44.4(4)
C33	1973.6(14)	9088.1(15)	2476.7(13)	33.9(3)
C34	4434.8(12)	6594.2(12)	1651.8(10)	24.2(3)
C35	4748.5(14)	6290.7(14)	814.5(11)	29.3(3)
C36	5956.5(14)	6009.3(16)	456.6(12)	34.6(3)
C37	6915.3(14)	6017.1(16)	930.8(13)	36.1(3)
C38	6649.6(15)	6311.5(18)	1757.8(14)	41.3(4)
C39	5441.2(15)	6593.9(17)	2104.5(13)	36.8(3)
B1	2993.5(14)	7055.6(14)	2026.3(11)	23.3(3)

Table S23. Anisotropic Displacement Parameters ($Å^2 \times 10^3$) for [Mn(PDMEA)(OAc)(MeOH)]BPh_4 (**5**). The Anisotropic displacement factor exponent takes the form: -$2\pi^2[h^2a^{*2}U_{11}+2hka^*b^*U_{12}+\ldots]$.

**Atom**	**U11**	**U22**	**U33**	**U23**	**U13**	**U12**
Mn1	21.74(10)	23.1(1)	22.05(10)	-8.80(8)	-0.05(7)	-4.77(7)
O1	23.9(5)	31.6(5)	35.3(5)	-20.0(4)	3.7(4)	-8.1(4)
O2	25.0(5)	24.6(5)	29.5(5)	-10.2(4)	0.0(4)	-0.8(4)
O3	31.3(5)	30.3(5)	26.1(5)	-10.9(4)	1.6(4)	1.1(4)
O4	32.6(5)	28.5(5)	25.6(5)	-11.0(4)	5.2(4)	-2.1(4)
O5	29.1(5)	21.1(4)	25.1(5)	-10.0(4)	-0.3(4)	-3.4(4)
N1	23.1(6)	33.0(6)	35.8(7)	-13.0(5)	-1.7(5)	-6.6(5)
N2	28.4(6)	26.5(6)	24.4(5)	-8.9(5)	2.6(4)	-4.7(5)
C1	30.8(7)	20.2(6)	29.8(7)	-5.7(5)	-0.6(5)	-2.1(5)
C2	34.5(8)	26.1(7)	24.8(7)	-4.6(5)	-2.9(6)	-0.6(6)
C3	42.3(9)	34.3(8)	22.0(6)	-11.3(6)	0.8(6)	-3.2(6)
C4	44.7(9)	36.8(8)	25.1(7)	-12.4(6)	6.9(6)	-2.9(7)
C5	36.7(8)	41.1(9)	33.1(8)	-9.2(7)	9.3(6)	-15.9(7)
C6	20.6(6)	29.2(7)	42.4(8)	-13.1(6)	4.3(6)	-6.0(5)
C7	29.4(8)	34.5(8)	63.0(12)	-18.4(8)	7.8(7)	-12.1(6)
C8	29.6(8)	52.5(11)	83.4(15)	-40.2(11)	5.5(9)	-17.0(8)
C9	32.7(8)	66.9(13)	61.9(12)	-38.5(11)	-2.3(8)	-16.1(9)
C10	31.1(8)	50.6(10)	41.6(9)	-19.3(8)	-5.8(7)	-12.3(7)
C11	22.5(6)	24.9(6)	24.8(6)	-10.3(5)	-1.3(5)	-6.5(5)
C12	30.0(7)	30.9(7)	24.1(6)	-7.7(5)	-2.7(5)	-5.2(6)
C13	39.1(8)	27.5(7)	34.9(8)	-13.1(6)	4.7(6)	-2.4(6)
C14	37.7(8)	42.8(9)	37.7(8)	-24.2(7)	0.8(6)	-16.2(7)
C15	46.5(9)	30.0(7)	32.5(8)	-17.7(6)	0.7(7)	-2.4(6)
C16	25.4(6)	22.6(6)	25.7(6)	-10.6(5)	-1.0(5)	-4.3(5)
C17	42.3(8)	42.1(9)	28.0(7)	-10.3(6)	-2.5(6)	-22.0(7)
C18	45.0(9)	42.4(9)	27.3(7)	-11.3(7)	-6.3(6)	-15.4(7)
C19	44.4(9)	30.6(7)	24.8(7)	-9.5(6)	2.3(6)	-8.7(6)
C20	40.5(8)	38.6(8)	32.2(8)	-12.4(7)	6.0(6)	-19.2(7)
C21	29.8(7)	34.1(7)	28.5(7)	-11.5(6)	-0.9(6)	-10.9(6)
C22	24.5(6)	28.6(6)	22.7(6)	-11.5(5)	1.6(5)	-7.8(5)
C23	35.2(8)	31.7(7)	32.8(7)	-15.5(6)	-1.5(6)	-10.5(6)
C24	42.8(9)	48.7(10)	40.6(9)	-26.7(8)	5.4(7)	-22.9(8)
C25	30.3(8)	69.0(12)	35.4(8)	-28.5(8)	4.7(6)	-23.3(8)
C26	22.2(6)	57.6(10)	30.3(7)	-19.5(7)	1.1(6)	-7.9(7)

C27	23.0(6)	35.3(7)	26.1(6)	-13.5(6)	1.1(5)	-5.1(5)
C28	26.7(6)	22.8(6)	28.8(7)	-10.4(5)	-7.9(5)	-3.5(5)
C29	44.1(9)	24.7(7)	30.3(7)	-7.1(6)	-8.4(6)	-8.3(6)
C30	65.2(12)	23.8(7)	38.9(9)	-1.5(7)	-19.6(8)	-11.9(7)
C31	64.7(12)	23.1(7)	55.3(11)	-14.3(8)	-30.5(9)	1.2(7)
C32	45.0(10)	34.1(8)	57.2(11)	-28.4(8)	-15.9(8)	6.0(7)
C33	31.5(7)	30.4(7)	42.0(8)	-19.2(7)	-4.8(6)	-2.8(6)
C34	25.3(6)	18.9(6)	26.3(6)	-7.0(5)	-2.2(5)	-5.2(5)
C35	28.0(7)	31.2(7)	25.4(6)	-9.6(6)	-2.7(5)	-4.8(5)
C36	33.0(8)	37.6(8)	28.7(7)	-12.7(6)	3.5(6)	-5.6(6)
C37	26.3(7)	34.6(8)	41.3(8)	-11.1(7)	3.8(6)	-7.0(6)
C38	28.3(7)	51.0(10)	52.9(10)	-27.2(9)	-2.4(7)	-11.9(7)
C39	30.4(7)	47.2(9)	44.9(9)	-29.4(8)	0.2(6)	-11.4(7)
B1	24.8(7)	21.5(6)	24.8(7)	-10.6(5)	-1.7(5)	-5.2(5)

Table S24. Bond Lengths for [Mn(PDMEA)(OAc)(MeOH)]BPh_4 (**5**).

Atom	Atom	Length/Å	Atom	Atom	Length/Å
Mn1	O1	2.1551(10)	C16	C17	1.403(2)
Mn1	O2	2.3445(10)	C16	C21	1.401(2)
Mn1	O3	2.2062(11)	C16	B1	1.659(2)
Mn1	O4	2.3361(10)	C17	C18	1.388(2)
Mn1	O5	2.3842(10)	C18	C19	1.377(2)
Mn1	N1	2.2471(13)	C19	C20	1.378(2)
Mn1	N2	2.3225(12)	C20	C21	1.393(2)
Mn1	C11	2.6273(14)	C22	C23	1.406(2)
O1	C14	1.4298(18)	C22	C27	1.401(2)
O2	C11	1.2683(16)	C22	B1	1.646(2)
O3	C11	1.2522(16)	C23	C24	1.388(2)
O4	C4	1.4240(18)	C24	C25	1.388(3)
O4	C13	1.4302(18)	C25	C26	1.382(3)
O5	C1	1.4234(17)	C26	C27	1.397(2)
O5	C15	1.4275(17)	C28	C29	1.401(2)
N1	C6	1.337(2)	C28	C33	1.394(2)
N1	C10	1.345(2)	C28	B1	1.647(2)
N2	C2	1.4798(19)	C29	C30	1.393(2)
N2	C3	1.478(2)	C30	C31	1.380(3)
N2	C5	1.479(2)	C31	C32	1.374(3)
C1	C2	1.502(2)	C32	C33	1.396(2)
C3	C4	1.507(2)	C34	C35	1.400(2)

Table S24. Bond Lengths for [Mn(PDMEA)(OAc)(MeOH)]BPh_4 (**5**).

C5	C6	1.500(2)	C34	C39	1.403(2)
C6	C7	1.397(2)	C34	B1	1.658(2)
C7	C8	1.369(3)	C35	C36	1.395(2)
C8	C9	1.381(3)	C36	C37	1.380(2)
C9	C10	1.374(3)	C37	C38	1.377(2)
C11	C12	1.5005(19)	C38	C39	1.390(2)

Table S25. Bond Angles for [Mn(PDMEA)(OAc)(MeOH)]BPh_4 (**5**).

**Atom**	**Atom**	**Atom**	**Angle/˚**	**Atom**	**Atom**	**Atom**	**Angle/˚**
O1	Mn1	O2	90.23(4)	N1	C6	C5	117.31(14)
O1	Mn1	O3	102.57(4)	N1	C6	C7	121.53(16)
O1	Mn1	O4	86.06(4)	C7	C6	C5	121.10(15)
O1	Mn1	O5	81.56(4)	C8	C7	C6	119.18(17)
O1	Mn1	N1	160.35(5)	C7	C8	C9	119.61(17)
O1	Mn1	N2	91.81(4)	C10	C9	C8	118.15(19)
O1	Mn1	C11	96.34(4)	N1	C10	C9	123.19(18)
O2	Mn1	O5	79.41(3)	O2	C11	Mn1	63.10(7)
O2	Mn1	C11	28.85(4)	O2	C11	C12	120.16(12)
O3	Mn1	O2	57.17(4)	O3	C11	Mn1	56.79(7)
O3	Mn1	O4	81.05(4)	O3	C11	O2	119.80(12)
O3	Mn1	O5	136.17(4)	O3	C11	C12	120.04(12)
O3	Mn1	N1	95.91(5)	C12	C11	Mn1	175.88(10)
O3	Mn1	N2	148.26(4)	C17	C16	B1	122.79(12)
O3	Mn1	C11	28.35(4)	C21	C16	C17	113.78(13)
O4	Mn1	O2	136.11(4)	C21	C16	B1	123.40(12)
O4	Mn1	O5	142.51(4)	C18	C17	C16	123.50(14)
O4	Mn1	C11	108.28(4)	C19	C18	C17	120.43(15)
O5	Mn1	C11	108.18(4)	C18	C19	C20	118.42(14)
N1	Mn1	O2	94.25(4)	C19	C20	C21	120.39(15)
N1	Mn1	O4	103.55(4)	C20	C21	C16	123.38(14)
N1	Mn1	O5	80.48(4)	C23	C22	B1	121.33(12)
N1	Mn1	N2	75.45(5)	C27	C22	C23	115.01(13)
N1	Mn1	C11	96.78(5)	C27	C22	B1	123.54(13)
N2	Mn1	O2	152.12(4)	C24	C23	C22	123.14(15)
N2	Mn1	O4	71.77(4)	C23	C24	C25	119.83(16)
N2	Mn1	O5	73.41(4)	C26	C25	C24	119.24(15)
N2	Mn1	C11	171.83(4)	C25	C26	C27	120.02(16)
C14	O1	Mn1	131.64(9)	C26	C27	C22	122.76(15)
C11	O2	Mn1	88.05(8)	C29	C28	B1	121.55(13)
C11	O3	Mn1	94.86(8)	C33	C28	C29	115.10(14)
C4	O4	Mn1	118.06(9)	C33	C28	B1	123.31(13)
C4	O4	C13	111.50(11)	C30	C29	C28	123.06(16)
C13	O4	Mn1	128.26(9)	C31	C30	C29	119.59(17)
C1	O5	Mn1	113.34(8)	C32	C31	C30	119.43(16)
C1	O5	C15	111.54(11)	C31	C32	C33	120.15(17)
C15	O5	Mn1	125.20(9)	C28	C33	C32	122.67(17)

C6	N1	Mn1	116.59(11)	C35	C34	C39	114.28(13)
C6	N1	C10	118.34(14)	C35	C34	B1	123.67(12)
C10	N1	Mn1	123.50(11)	C39	C34	B1	121.79(13)
C2	N2	Mn1	107.47(8)	C36	C35	C34	123.18(14)
C3	N2	Mn1	108.86(9)	C37	C36	C35	120.32(15)
C3	N2	C2	108.80(12)	C38	C37	C36	118.51(15)
C3	N2	C5	111.25(12)	C37	C38	C39	120.56(16)
C5	N2	Mn1	109.31(9)	C38	C39	C34	123.15(15)
C5	N2	C2	111.06(13)	C22	B1	C16	108.22(11)
O5	C1	C2	107.14(11)	C22	B1	C28	109.69(11)
N2	C2	C1	112.28(12)	C22	B1	C34	110.52(11)
N2	C3	C4	111.69(13)	C28	B1	C16	110.06(11)
O4	C4	C3	108.24(12)	C28	B1	C34	106.33(11)
N2	C5	C6	113.79(12)	C34	B1	C16	112.01(11)

Table S26. Hydrogen Atom Coordinates (Å×10^4) and Isotropic Displacement Parameters (Å^2×10^3) for [Mn(PDMEA)(OAc)(MeOH)]BPh_4 (**5**).

**Atom**	**x**	**y**	**z**	**U(eq)**
H1	4772(12)	30(18)	3779(12)	42
H1A	6198.42	3629.87	2729.12	35
H1B	4751.55	3898.15	2575.48	35
H2A	5192.99	2411.81	1847.3	38
H2B	5913.94	3447.84	1249.97	38
H3A	7295.94	1680.87	691.05	42
H3B	6352.19	927.62	1372.96	42
H4A	8105.59	-598.21	1272.08	46
H4B	8981.87	76.05	1495.2	46
H5A	7897.52	3128	1436.68	46
H5B	8825.29	1746.74	1684.19	46
H7	9154.49	3787.65	2242.43	51
H8	9760.9	3688.84	3721.88	60
H9	9418.27	2101.25	5219.52	58
H10	8487.56	668.47	5180.16	48
H12A	7401.69	-3094.23	6563.14	45
H12B	6684.25	-2031.89	6983.4	45
H12C	8153.13	-2357.45	6842.53	45
H13A	9767.75	-1962.32	2802.79	54
H13B	8734.36	-2519.09	2656.66	54
H13C	8869.53	-2477.43	3705.71	54
H14A	5495.47	-703.26	2308.65	53
H14B	4384.85	-975.64	3077.64	53
H14C	5774.27	-1806.22	3384.93	53
H15A	4887.1	1936.78	4975.5	55
H15B	4479.59	3378.19	4218.03	55
H15C	5863.8	2744.26	4618.27	55
H17	4024.84	6606.54	3806.66	43
H18	3783.76	5375.96	5452.74	45
H19	2342.47	4218.54	5928.72	41
H20	1114.28	4369.57	4716.14	43
H21	1389.53	5569.74	3063.58	37
H23	2758.16	5058.78	1656.84	38
H24	1325.57	4799.09	842.64	47
H25	-326.61	6522.78	21.13	48
H26	-497.42	8502.13	17.91	44

H27	940.35	8748.92	847.15	34
H29	3749.95	9051.38	589.24	40
H30	3367.28	11170.91	297.85	53
H31	2086.14	12002.83	1380.8	58
H32	1204.37	10694.59	2746.06	53
H33	1607.21	8570.36	3040.21	41
H35	4107.83	6275.58	473.43	35
H36	6119.69	5811.31	-116.48	41
H37	7740.25	5823.77	692.08	43
H38	7297.73	6321.67	2094.26	50
H39	5289.29	6796.36	2675.36	44

X-ray crystallographic tables for [Mn(TEA)(OAc)(MeOH)]BPh_4·2 MeOH (6)

Table S27. Crystal data and structure refinement for [Mn(TEA)(OAc)(MeOH)]BPh_4·2 MeOH (**6**).

Identification code	exp_03_auto
Empirical formula	$C_{35}H_{50}BMnNO_8$
Formula weight	678.51 g/mol
Temperature/K	298.82(15)
Crystal system	monoclinic
Space group	$P2_1/c$
a/Å	10.0738(2)
b/Å	14.8304(3)
c/Å	24.6141(4)
α/°	90
β/°	99.3840(10)
γ/°	90
Volume/Å^3	3628.10(12)
Z	4
ρ_{calc}g/cm^3	1.242
μ/mm^{-1}	3.354
F(000)	1444.0
Crystal size/mm^3	0.369 × 0.149 × 0.044
Radiation	Cu Kα (λ = 1.54184)
2Θ range for data collection/°	6.984 to 154.806
Index ranges	-12 ≤ h ≤ 8, -18 ≤ k ≤ 18, -31 ≤ l ≤ 29
Reflections collected	34992
Independent reflections	7238 [R_{int} = 0.0325, R_{sigma} = 0.0242]
Data/restraints/parameters	7238/6/430
Goodness-of-fit on F^2	1.055
Final R indexes [I>=2σ (I)]	R_1 = 0.0402, wR_2 = 0.1009
Final R indexes [all data]	R_1 = 0.0483, wR_2 = 0.1049
Largest diff. peak/hole / e Å^{-3}	0.30/-0.31

Table S28. Fractional Atomic Coordinates (×10^4) and Equivalent Isotropic Displacement Parameters (Å^2×10^3) for [Mn(TEA)(OAc)(MeOH)]BPh_4·2 MeOH (**6**). U_{eq} is defined as 1/3 of the trace of the orthogonalised U_{IJ} tensor.

**Atom**	***x***	***y***	***z***	**U(eq)**
C10	2088.8(17)	8212.8(13)	3099.5(7)	43.8(4)
C11	3217.5(19)	8336.7(15)	2847.1(8)	51.9(5)
C12	3669(2)	7698.4(18)	2510.7(10)	66.7(6)
C13	3018(3)	6890.8(19)	2414.1(9)	70.3(7)
C14	1913(2)	6719.8(17)	2663.1(10)	68.3(6)
C15	1466(2)	7374.0(15)	2994.9(9)	57.3(5)
C16	1822.3(18)	8728.6(14)	4140.3(8)	47.6(4)
C17	2145(2)	7867.7(16)	4351.1(9)	57.0(5)
C18	2332(3)	7673.0(19)	4911.6(10)	74.1(7)
C19	2227(3)	8338(2)	5284.5(10)	85.3(8)
C20	1925(3)	9199(2)	5099.9(10)	85.7(8)
C21	1732(3)	9389.5(18)	4539.9(9)	68.2(6)
C22	-171.6(18)	9015.1(13)	3287.2(8)	45.8(4)
C23	-1090(2)	9163.1(17)	3645.0(10)	64.1(6)
C24	-2459(2)	9248(2)	3465.1(13)	82.5(8)
C25	-2980(2)	9186.7(19)	2918.5(13)	78.2(8)
C26	-2121(2)	9022.4(18)	2551.2(11)	71.3(7)
C27	-756(2)	8938.8(17)	2735.4(9)	59.8(5)
C28	2122(2)	9972.4(14)	3385.3(8)	48.8(4)
C29	3479(2)	10160.8(18)	3576.1(11)	68.3(6)
C30	4070(3)	10982(2)	3474.3(12)	83.7(8)
C31	3311(3)	11646.0(19)	3187.0(11)	79.7(8)
C32	1979(3)	11502.9(16)	3009.9(10)	70.1(7)
C33	1406(2)	10682.0(14)	3109.0(9)	56.1(5)
B1	1477(2)	8977.5(15)	3480.7(9)	44.2(5)
Mn1	2820.0(3)	10214.4(2)	587.1(2)	40.62(10)
O1	1809.2(14)	9168.9(11)	1014.1(5)	59.1(4)
O2	1046.8(12)	11146.0(10)	491.0(5)	49.5(3)
O3	4257.5(16)	11419.6(11)	708.4(6)	62.2(4)
O4	4582.8(14)	9339.2(11)	738.9(6)	59.3(4)
O5	1428.1(13)	9573.9(10)	-225.9(6)	52.3(3)
O6	3271.6(13)	10325.3(10)	-255.1(5)	50.8(3)
N1	2850.6(15)	10760.4(12)	1472.0(6)	49.8(4)
C1	1623(2)	9345.9(18)	1568.7(8)	60.6(6)
C2	2719(2)	9966.6(17)	1824.3(8)	58.5(5)
C3	1715(2)	11403.2(16)	1474.1(8)	57.7(5)

Table S28. Fractional Atomic Coordinates (×10^4) and Equivalent Isotropic Displacement Parameters (Å^2×10^3) for [Mn(TEA)(OAc)(MeOH)]BPh_4·2 MeOH (**6**). U_{eq} is defined as 1/3 of the trace of the orthogonalised U_{IJ} tensor.

**Atom**	***x***	***y***	***z***	**U(eq)**
C4	1223(2)	11818.4(15)	918.2(9)	55.5(5)
C5	4174(2)	11181.2(19)	1649.7(9)	66.1(6)
C6	4528(2)	11836.5(18)	1240.8(10)	68.8(6)
C7	4912(3)	8541.9(17)	1043.9(11)	70.8(6)
C8	2277.8(18)	9882.5(13)	-495.8(7)	43.9(4)
C9	2140(2)	9734.4(19)	-1101.9(9)	65.7(6)
O7	447(2)	13300.9(15)	4359.1(7)	86.3(6)
C34	842(5)	12486(3)	4166(2)	157(2)
O8	5447(3)	7898(2)	4901.0(17)	146.4(11)
C35	5991(5)	8716(3)	4935.8(18)	131.3(14)

Table S29. Anisotropic Displacement Parameters (Å^2×10^3) for [Mn(TEA)(OAc)(MeOH)]BPh_4·2 MeOH (**6**). The Anisotropic displacement factor exponent takes the form: -2π^2[h^{2}a*^{2}U$_{11}$+2hka*b*U$_{12}$+…].

**Atom**	**U_{11}**	**U_{22}**	**U_{33}**	**U_{23}**	**U_{13}**	**U_{12}**
C10	38.7(9)	50.7(11)	41.8(9)	3.0(8)	6.1(7)	1.1(8)
C11	46.0(10)	55.3(12)	57.1(11)	11.8(9)	16.2(9)	6.7(9)
C12	65.1(14)	77.2(17)	63.4(13)	16.4(12)	27.3(11)	23.9(12)
C13	77.4(16)	80.3(18)	52.3(12)	-6.8(12)	8.1(11)	31.0(14)
C14	69.0(14)	61.5(14)	70.5(14)	-16.7(12)	-0.2(11)	2.5(11)
C15	50.4(11)	60.1(13)	62.5(12)	-10.0(10)	13.1(9)	-6.6(10)
C16	41.5(9)	54.0(11)	47.9(10)	-1.8(9)	9.2(8)	-9.0(8)
C17	57.2(12)	57.8(13)	54.6(11)	0.2(10)	4.5(9)	-10.5(10)
C18	86.6(17)	72.2(16)	59.5(14)	13.1(12)	0.2(12)	-16.1(13)
C19	104(2)	102(2)	48.4(12)	7.2(14)	7.3(13)	-25.1(18)
C20	111(2)	93(2)	53.9(13)	-18.0(14)	17.1(14)	-10.9(17)
C21	85.6(16)	65.0(14)	54.6(12)	-7.2(11)	13.5(11)	-2.0(13)
C22	42.1(9)	43.3(10)	53.7(10)	0.7(8)	12.8(8)	-4.7(8)
C23	50.6(12)	78.3(16)	66.8(13)	-6.9(12)	19.4(10)	1.2(11)
C24	51.1(13)	103(2)	99(2)	-4.3(17)	30.2(13)	5.4(13)
C25	38.9(11)	84.2(18)	110(2)	3.6(16)	6.9(12)	-2.1(11)
C26	54.4(13)	82.3(18)	72.5(15)	6.6(13)	-3.7(11)	-5.1(12)
C27	45.7(11)	75.9(15)	58.0(12)	3.2(11)	9.3(9)	-2.4(10)
C28	49.2(10)	52.4(11)	47.2(10)	-4.3(8)	15.0(8)	-8.0(9)
C29	57.1(13)	71.7(15)	74.8(15)	2.6(12)	7.2(11)	-17.5(12)
C30	69.9(16)	96(2)	88.2(18)	-15.7(17)	21.2(14)	-40.1(16)

Table S29. Anisotropic Displacement Parameters ($Å^2×10^3$) for [Mn(TEA)(OAc)(MeOH)]BPh_4·2 MeOH (**6**). The Anisotropic displacement factor exponent takes the form: -$2\pi^2[h^2a^{*2}U_{11}+2hka^*b^*U_{12}+…]$.

Atom	U_{11}	U_{22}	U_{33}	U_{23}	U_{13}	U_{12}
C31	114(2)	60.4(15)	74.0(16)	-13.7(13)	42.4(16)	-34.6(16)
C32	103(2)	48.9(13)	63.8(14)	-4.5(11)	29.9(13)	-8.3(13)
C33	65.7(13)	49.3(12)	56.2(11)	-3.1(10)	18.7(10)	-3.0(10)
B1	39.4(10)	47.2(12)	47.1(11)	0.6(9)	10.7(8)	-3.8(9)
Mn1	35.70(15)	51.14(18)	36.33(15)	-1.94(12)	9.77(10)	2.14(12)
O1	54.7(8)	78.0(11)	46.0(7)	3.0(7)	11.8(6)	-12.3(7)
O2	37.2(6)	62.1(9)	48.9(7)	-7.7(6)	6.0(5)	4.5(6)
O3	71.6(9)	63.3(10)	53.5(8)	-10.5(7)	15.8(7)	-18.4(8)
O4	43.6(7)	67.3(9)	71.6(9)	23.2(8)	22.7(6)	14.4(7)
O5	47.6(7)	60.4(9)	52.0(7)	-2.7(6)	17.6(6)	-3.4(6)
O6	40.9(7)	70.1(9)	42.4(7)	-3.8(6)	10.3(5)	-2.6(6)
N1	38.1(8)	70.3(11)	41.0(8)	-7.3(8)	6.4(6)	1.5(7)
C1	55.3(12)	82.9(16)	46.9(10)	12.5(11)	18.3(9)	3.7(11)
C2	56.2(12)	83.3(16)	36.2(9)	3.6(10)	8.3(8)	10.3(11)
C3	53.5(11)	71.5(14)	50.3(11)	-13.2(10)	15.6(9)	8.3(10)
C4	48.6(11)	57.1(12)	61.3(12)	-10.2(10)	10.9(9)	9.1(9)
C5	51.4(12)	95.4(18)	50.1(11)	-16.8(12)	4.2(9)	-9.0(12)
C6	61.0(13)	79.0(16)	66.8(14)	-22.8(12)	11.5(11)	-18.0(12)
C7	70.8(15)	65.4(15)	77.7(15)	19.1(12)	16.2(12)	14.0(12)
C8	40.6(9)	50.0(10)	41.8(9)	-1.9(8)	8.4(7)	7.8(8)
C9	60.3(13)	93.1(18)	44.6(11)	-10.0(11)	11.4(9)	-7.6(12)
O7	85.5(12)	104.1(15)	66.6(10)	-11.3(10)	4.5(9)	26.6(11)
C34	141(4)	139(4)	199(5)	-64(4)	48(3)	43(3)
O8	128(2)	111(2)	214(3)	-65(2)	67(2)	-14.3(17)
C35	162(4)	97(3)	135(3)	-11(2)	22(3)	-23(3)

Table S30. Bond Lengths for [Mn(TEA)(OAc)(MeOH)]BPh_4·2 MeOH (**6**).

Atom	Atom	Length/Å	Atom	Atom	Length/Å
C10	C11	1.394(3)	C30	C31	1.371(4)
C10	C15	1.398(3)	C31	C32	1.358(4)
C10	B1	1.654(3)	C32	C33	1.386(3)
C11	C12	1.382(3)	Mn1	O1	2.2123(15)
C12	C13	1.367(4)	Mn1	O2	2.2401(13)
C13	C14	1.380(4)	Mn1	O3	2.2891(15)
C14	C15	1.389(3)	Mn1	O4	2.1821(14)
C16	C17	1.396(3)	Mn1	O5	2.4410(14)

Table S30. Bond Lengths for [Mn(TEA)(OAc)(MeOH)]BPh_4·2 MeOH (**6**).

Atom	Atom	Length/Å	Atom	Atom	Length/Å
C16	C21	1.402(3)	Mn1	O6	2.1998(13)
C16	B1	1.646(3)	Mn1	N1	2.3192(15)
C17	C18	1.392(3)	O1	C1	1.432(2)
C18	C19	1.363(4)	O2	C4	1.439(2)
C19	C20	1.374(4)	O3	C6	1.435(3)
C20	C21	1.389(3)	O4	C7	1.411(3)
C22	C23	1.395(3)	O5	C8	1.252(2)
C22	C27	1.394(3)	O6	C8	1.262(2)
C22	B1	1.653(3)	N1	C2	1.481(3)
C23	C24	1.384(3)	N1	C3	1.490(3)
C24	C25	1.365(4)	N1	C5	1.473(3)
C25	C26	1.372(4)	C1	C2	1.495(3)
C26	C27	1.381(3)	C3	C4	1.509(3)
C28	C29	1.400(3)	C5	C6	1.484(4)
C28	C33	1.389(3)	C8	C9	1.492(3)
C28	B1	1.644(3)	O7	C34	1.380(4)
C29	C30	1.396(4)	O8	C35	1.328(4)

Table S31. Bond Angles for [Mn(TEA)(OAc)(MeOH)]BPh_4·2 MeOH (**6**).

Atom	Atom	Atom	Angle/˚	Atom	Atom	Atom	Angle/˚
C11	C10	C15	114.17(18)	O1	Mn1	O5	82.49(5)
C11	C10	B1	124.67(18)	O1	Mn1	N1	74.65(6)
C15	C10	B1	121.15(16)	O2	Mn1	O3	90.50(6)
C12	C11	C10	123.3(2)	O2	Mn1	O5	79.16(5)
C13	C12	C11	120.6(2)	O2	Mn1	N1	76.83(5)
C12	C13	C14	118.8(2)	O3	Mn1	O5	132.64(5)
C13	C14	C15	119.7(2)	O3	Mn1	N1	72.16(6)
C14	C15	C10	123.4(2)	O4	Mn1	O1	85.79(6)
C17	C16	C21	114.60(19)	O4	Mn1	O2	176.07(6)
C17	C16	B1	124.79(18)	O4	Mn1	O3	87.84(6)
C21	C16	B1	120.52(19)	O4	Mn1	O5	104.57(6)
C18	C17	C16	122.9(2)	O4	Mn1	O6	85.31(5)
C19	C18	C17	120.3(3)	O4	Mn1	N1	99.27(6)
C18	C19	C20	119.2(2)	O6	Mn1	O1	133.00(5)
C19	C20	C21	120.3(3)	O6	Mn1	O2	97.95(5)
C20	C21	C16	122.7(3)	O6	Mn1	O3	80.84(5)
C23	C22	B1	124.23(18)	O6	Mn1	O5	55.66(5)
C27	C22	C23	114.19(19)	O6	Mn1	N1	152.35(6)

Table S31. Bond Angles for [Mn(TEA)(OAc)(MeOH)]BPh_4·2 MeOH (**6**).

Atom	Atom	Atom	Angle/˚	Atom	Atom	Atom	Angle/˚
C27	C22	B1	121.52(17)	N1	Mn1	O5	145.46(5)
C24	C23	C22	122.8(2)	C1	O1	Mn1	117.80(14)
C25	C24	C23	120.8(2)	C4	O2	Mn1	110.41(11)
C24	C25	C26	118.6(2)	C6	O3	Mn1	118.54(13)
C25	C26	C27	120.1(2)	C7	O4	Mn1	134.74(13)
C26	C27	C22	123.5(2)	C8	O5	Mn1	86.66(11)
C29	C28	B1	121.20(19)	C8	O6	Mn1	97.66(11)
C33	C28	C29	114.4(2)	C2	N1	Mn1	106.48(12)
C33	C28	B1	124.35(18)	C2	N1	C3	111.38(16)
C30	C29	C28	122.4(3)	C3	N1	Mn1	109.52(11)
C31	C30	C29	120.1(3)	C5	N1	Mn1	107.43(11)
C32	C31	C30	119.5(2)	C5	N1	C2	109.04(16)
C31	C32	C33	119.9(3)	C5	N1	C3	112.73(18)
C32	C33	C28	123.6(2)	O1	C1	C2	108.12(16)
C16	B1	C10	111.57(16)	N1	C2	C1	111.94(16)
C16	B1	C22	109.42(15)	N1	C3	C4	113.37(16)
C22	B1	C10	108.03(15)	O2	C4	C3	111.30(17)
C28	B1	C10	110.01(15)	N1	C5	C6	112.48(18)
C28	B1	C16	108.50(16)	O3	C6	C5	107.84(19)
C28	B1	C22	109.28(16)	O5	C8	O6	119.99(16)
O1	Mn1	O2	93.53(6)	O5	C8	C9	121.10(18)
O1	Mn1	O3	144.66(5)	O6	C8	C9	118.91(17)

Table S32. Torsion Angles for [Mn(TEA)(OAc)(MeOH)]BPh_4·2 MeOH (**6**).

A	B	C	D	Angle/˚	A	B	C	D	Angle/˚
C10	C11	C12	C13	-0.9(3)	C29	C28	B1	C10	-69.3(2)
C11	C10	C15	C14	-0.8(3)	C29	C28	B1	C16	53.0(2)
C11	C10	B1	C16	-105.4(2)	C29	C28	B1	C22	172.25(19)
C11	C10	B1	C22	134.25(18)	C29	C30	C31	C32	0.9(4)
C11	C10	B1	C28	15.0(3)	C30	C31	C32	C33	-1.5(4)
C11	C12	C13	C14	-0.7(3)	C31	C32	C33	C28	0.0(3)
C12	C13	C14	C15	1.4(4)	C33	C28	C29	C30	-2.7(3)
C13	C14	C15	C10	-0.6(4)	C33	C28	B1	C10	109.6(2)
C15	C10	C11	C12	1.6(3)	C33	C28	B1	C16	-128.1(2)
C15	C10	B1	C16	76.0(2)	C33	C28	B1	C22	-8.8(3)
C15	C10	B1	C22	-44.3(2)	B1	C10	C11	C12	-177.03(19)
C15	C10	B1	C28	-163.48(18)	B1	C10	C15	C14	177.8(2)
C16	C17	C18	C19	-1.2(4)	B1	C16	C17	C18	-175.0(2)

Table S32. Torsion Angles for [Mn(TEA)(OAc)(MeOH)]BPh_4·2 MeOH (**6**).

A	B	C	D	Angle/˚	A	B	C	D	Angle/˚
C17	C16	C21	C20	-1.1(3)	B1	C16	C21	C20	175.6(2)
C17	C16	B1	C10	-21.3(2)	B1	C22	C23	C24	175.7(2)
C17	C16	B1	C22	98.2(2)	B1	C22	C27	C26	-175.8(2)
C17	C16	B1	C28	-142.65(19)	B1	C28	C29	C30	176.3(2)
C17	C18	C19	C20	0.5(4)	B1	C28	C33	C32	-176.90(19)
C18	C19	C20	C21	-0.1(5)	Mn1	O1	C1	C2	-28.0(2)
C19	C20	C21	C16	0.5(4)	Mn1	O2	C4	C3	-45.47(19)
C21	C16	C17	C18	1.5(3)	Mn1	O3	C6	C5	19.9(2)
C21	C16	B1	C10	162.39(18)	Mn1	O5	C8	O6	-1.47(18)
C21	C16	B1	C22	-78.1(2)	Mn1	O5	C8	C9	178.39(19)
C21	C16	B1	C28	41.0(2)	Mn1	O6	C8	O5	1.6(2)
C22	C23	C24	C25	0.3(5)	Mn1	O6	C8	C9	-178.22(17)
C23	C22	C27	C26	1.5(3)	Mn1	N1	C2	C1	-46.55(19)
C23	C22	B1	C10	144.4(2)	Mn1	N1	C3	C4	-23.4(2)
C23	C22	B1	C16	22.7(3)	Mn1	N1	C5	C6	51.0(2)
C23	C22	B1	C28	-96.0(2)	O1	C1	C2	N1	50.0(2)
C23	C24	C25	C26	1.1(5)	N1	C3	C4	O2	46.7(2)
C24	C25	C26	C27	-1.1(4)	N1	C5	C6	O3	-47.1(3)
C25	C26	C27	C22	-0.2(4)	C2	N1	C3	C4	-140.91(19)
C27	C22	C23	C24	-1.5(4)	C2	N1	C5	C6	166.00(19)
C27	C22	B1	C10	-38.6(2)	C3	N1	C2	C1	72.8(2)
C27	C22	B1	C16	-160.28(19)	C3	N1	C5	C6	-69.8(2)
C27	C22	B1	C28	81.0(2)	C5	N1	C2	C1	-162.18(18)
C28	C29	C30	C31	1.3(4)	C5	N1	C3	C4	96.1(2)
C29	C28	C33	C32	2.1(3)					

Table S33. Hydrogen Atom Coordinates (Å×10^4) and Isotropic Displacement Parameters (Å^2×10^3) for [Mn(TEA)(OAc)(MeOH)]BPh_4·2 MeOH (**6**).

Atom	*x*	*y*	*z*	U(eq)
H11	3690.61	8875.55	2908.12	62
H12	4421.24	7819.45	2348.53	80
H13	3316.15	6464.16	2184.53	84
H14	1468.41	6169.38	2609.16	82
H15	713.83	7247.19	3155.94	69
H17	2239.23	7403.68	4106.11	68
H18	2529.08	7086.55	5032.17	89
H19	2357.76	8209.54	5659.33	102
H20	1849.94	9657.72	5350.93	103

Table S33. Hydrogen Atom Coordinates (Å×10^4) and Isotropic Displacement Parameters (Å^2×10^3) for [Mn(TEA)(OAc)(MeOH)]BPh_4·2 MeOH (**6**).

**Atom**	***x***	***y***	***z***	**U(eq)**
H21	1535.35	9979.01	4425.5	82
H23	-769.24	9206.3	4020.36	77
H24	-3031.61	9349.1	3719.61	99
H25	-3898.37	9254.63	2797.6	94
H26	-2457.11	8967.25	2177.73	86
H27	-196.6	8825.6	2477.57	72
H29	4006.55	9722.92	3778.03	82
H30	4979.95	11077.87	3601.42	100
H31	3704.96	12190.35	3113.99	96
H32	1451.79	11955.3	2822.45	84
H33	491.65	10601.74	2983.64	67
H1	1153.22	8897.03	851.98	89
H2	209(10)	10991(13)	437(9)	74
H3	4280.81	11802.13	469.57	93
H4	5252(15)	9499(14)	594(10)	89
H1A	1659.2	8786.94	1775.3	73
H1B	751.45	9623.04	1571.28	73
H2A	2529.94	10169.43	2178.49	70
H2B	3564.68	9640.52	1886.98	70
H3A	2001.95	11880.71	1736.42	69
H3B	973.71	11088.55	1597.13	69
H4A	373.83	12121.94	925.98	67
H4B	1867.79	12265.08	838.01	67
H5A	4856.71	10714.11	1709.13	79
H5B	4170.71	11487.97	1997.49	79
H6A	3996.09	12380.84	1244.6	83
H6B	5472.31	11997.34	1329.18	83
H7A	5456.3	8688.47	1390.1	106
H7B	5402.71	8144.82	840.74	106
H7C	4101.62	8249.96	1108.34	106
H9A	1839.23	10280.99	-1291.88	99
H9B	1497.39	9263.71	-1210.26	99
H9C	2995.95	9563.21	-1193.48	99
H7	684.48	13326.74	4693.56	129
H34A	761.41	12022.11	4430.41	236
H34B	1760.49	12527.39	4109.51	236
H34C	277.93	12343.27	3823.61	236

Table S33. Hydrogen Atom Coordinates (Å×10^4) and Isotropic Displacement Parameters (Å^2×10^3) for [Mn(TEA)(OAc)(MeOH)]BPh_4·2 MeOH (**6**).

**Atom**	***x***	***y***	***z***	**U(eq)**
H8	4684.68	7924.3	4976.41	220
H35A	5832.73	9000.72	5269.52	197
H35B	5590.57	9069.88	4625.39	197
H35C	6941.68	8667.64	4937.5	197

Potentiometric titration data

Table S34. Quantities Used for the Potentiometric Titration of Ligands

Ligand	Ligand (mmoles)	HCl (mmoles)	$MnCl_2$ (mmoles)	[KCl] (M)	[NaOH] (M)
TMPA	0.0500	0.1000	0.0500	0.100	0.0100
DPEA	0.0500	0.1000	0.0500	0.100	0.0100
DPMEA	0.0500	0.1500	0.0500	0.100	0.0100
PDEA	0.200	0.300	0.200	0.100	0.0500
PDMEA	0.200	0.300	0.200	0.100	0.0500
TEA	0.500	1.00	0.500	0.100	0.0500

Table S35. Protonation Constants of the Tripodal Ligands Examined in this Study, Stability Constants of Their Mn^{2+} Complexes, and Acid Dissociation Constant of an Associated Aqua Ligand, $MnL(H_2O)$ (I = 0.10 M KCl, t = 25°C).

	TMPA	DPEA	DPMEA	PDEA	PDMEA	TEA
**Log K_{a1}**	6.01(1)	6.10(1)	6.19(1)	6.78(1)	6.90(2)	7.783(4)
**Log K_{a2}**	10.08(1)	9.40(3)	9.81(1)	N/A	N/A	N/A
**Log β_{MnL}**	5.24(2)	4.12(1)	3.71(2)	2.38(2)	2.32(2)	0*
**Log K_a $MnL(H_2O)$**	-4.98(3)	-5.63(1)	-6.07(2)	-7.00(2)	-6.6(1)	N/A

*Could not measure at these concentrations.

Potentiometric Titration Data

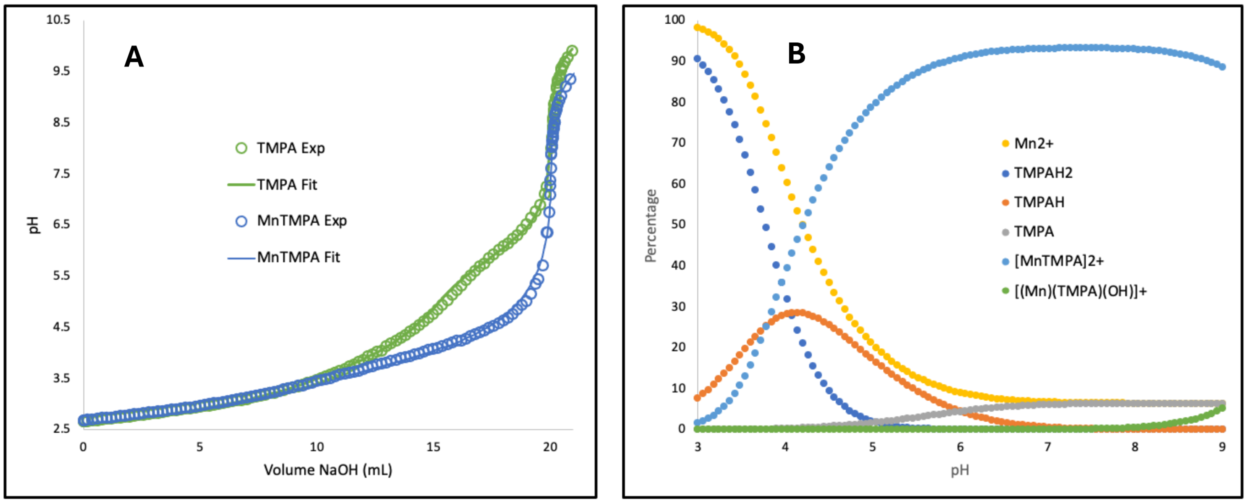

Figure S23. (A) Potentiometric titrations of 0.0500 mmoles tris(pyridin-2-ylmethyl)amine (TMPA) and 0.100 mmoles HCl in DI water containing 0.100 M KCl by 0.0100 M NaOH (green circles), and 0.0500 mmoles TMPA, 0.100 mmoles HCl, and 0.0500 mmoles $MnCl_2$ in DI water containing 0.100 M KCl by 0.0100 M NaOH (blue circles). The protonation constants for TMPA and stability constant for $[MnTMPA(H_2O)_x]^{2+}$ complex formation were determined by a non-linear, least squares fitting (Hyperquad 2013[1]) of the potentiometric data (solid lines) to speciation models in which the TMPA binds two protons and forms a mononuclear complex with Mn^{2+}. The K_a value of a coordinated water molecule is also accounted for in this model. (B) Speciation plot (HYSS[2]) produced from the protonation constants for the TMPA ligand, stability constant (β_{MnL}) for the $[MnTMPA(H_2O)_x]^{2+}$ complex, and K_a value of a coordinated water molecule, where [TMPA] = $[Mn^{2+}]$ = 1.25 mM.

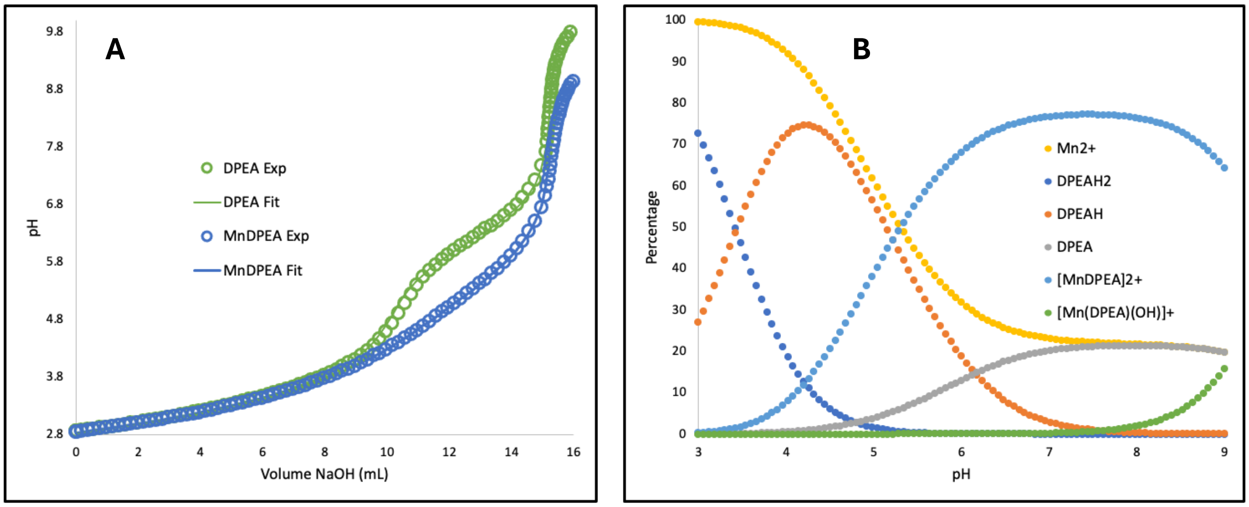

Figure S24. (A) Potentiometric titrations of 0.0500 N,N-bis(2-pyridylmethyl)ethanolamine (DPEA) and 0.100 mmoles HCl in DI water containing 0.100 M KCl by 0.0100 M NaOH (green circles), and 0.0500 mmoles DPEA, 0.100 mmoles HCl, and 0.0500 mmoles $MnCl_2$ in DI water containing 0.100 M KCl by 0.0100 M NaOH (blue circles). The protonation constants for DPEA and stability constant for $[MnDPEA(H_2O)_x]^{2+}$ complex formation were determined by a non-linear, least squares fitting (Hyperquad 2013[1]) of the potentiometric data (solid lines) to speciation models in which the DPEA binds two protons and forms a mononuclear complex with manganese(II). The K_a of a coordinated water molecule is also accounted for in this model. (B) Speciation plot (HYSS[2]) produced from the protonation constants determined for the DPEA ligand, stability constant (β_{MnL}) for the $[MnDPEA(H_2O)_x]^{2+}$ complex, and K_a value of a coordinated water molecule, where [DPEA] = $[Mn^{2+}]$ = 1.25 mM.

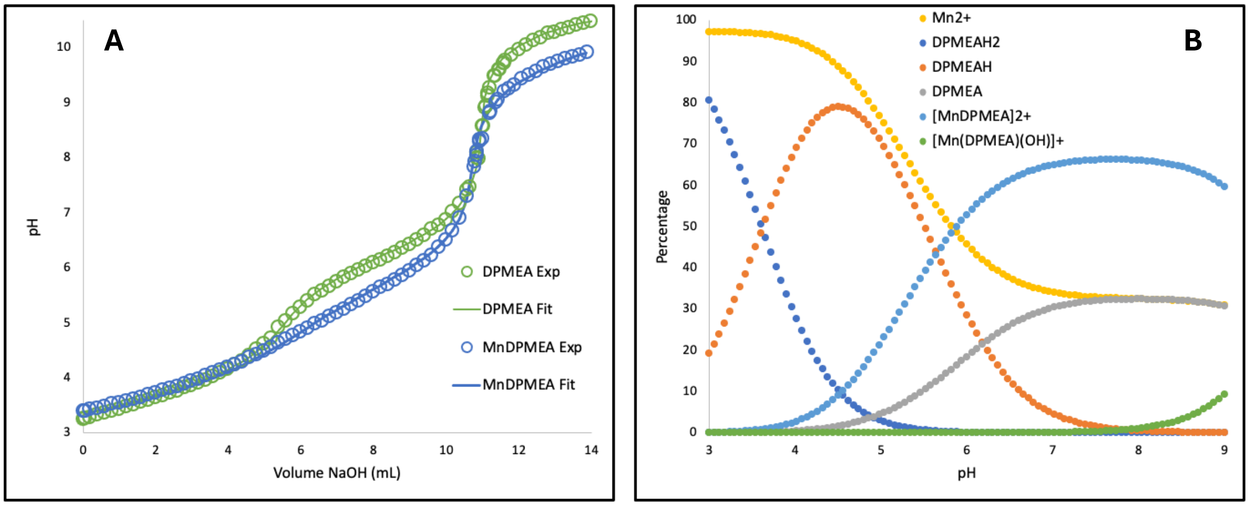

Figure S25. (A) Potentiometric titrations of 0.0500 2-Methoxy-N,N-bis(pyridin-2-ylmethyl)ethan-1-amine (DPMEA) and 0.150 mmoles HCl in DI water containing 0.100 M KCl by 0.0100 M NaOH (green circles), and 0.0500 mmoles DPMEA, 0.150 mmoles HCl, and 0.0500 mmoles $MnCl_2$ in DI water containing 0.100 M KCl by 0.0100 M NaOH (blue circles). The protonation constants for DPMEA and stability constant for $[MnDPMEA(H_2O)_x]^{2+}$ complex formation were determined by a non-linear, least squares fitting (Hyperquad 2013[1]) of the potentiometric data (solid lines) to speciation models in which the DPMEA binds two protons and forms a mononuclear complex with manganese(II). The K_a of a coordinated water molecule is also accounted for in this model. (B) Speciation plot (HYSS[2]) produced from the protonation constants determined for the DPMEA ligand, stability constant (β_{MnL}) for the $[MnDPMEA(H_2O)_x]^{2+}$ complex, and pK_a value of a coordinated water molecule, where [DPMEA] = $[Mn^{2+}]$ = 1.25 mM.

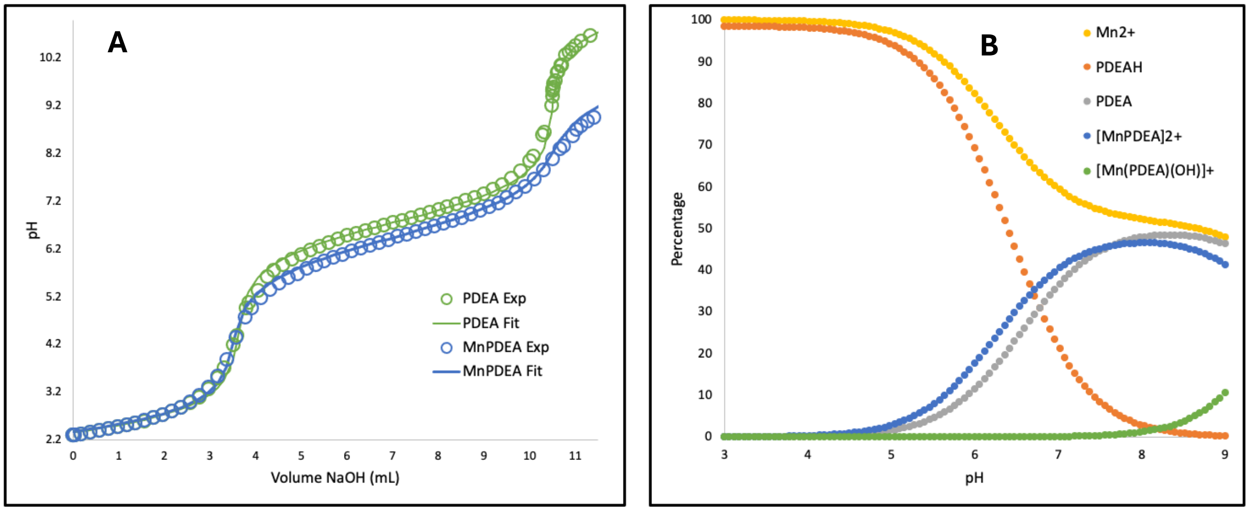

Figure S26. (A) Potentiometric titrations of 0.200 2,2'-((Pyridin-2-ylmethyl)azanediyl)bis(ethan-1-ol) (PDEA) and 0.300 mmoles HCl in DI water containing 0.100 M KCl by 0.0500 M NaOH (green circles), and 0.200 mmoles PDEA, 0.300 mmoles HCl, and 0.200 mmoles $MnCl_2$ in DI water containing 0.100 M KCl by 0.0500 M NaOH (blue circles). The protonation constant for PDEA and stability constant for $[MnPDEA(H_2O)_x]^{2+}$ complex formation were determined by a non-linear, least squares fitting (Hyperquad 2013[1]) of the potentiometric data (solid lines) to speciation models in which the PDEA binds one proton and forms a mononuclear complex with manganese(II). The K_a of a coordinated water molecule is also accounted for in this model. (B) Speciation plot (HYSS[2]) produced from the protonation constants for the PDEA ligand, stability constant (β_{MnL}) for the $[MnPDEA(H_2O)_x]^{2+}$ complex, and K_a value of a coordinated water molecule, where [PDEA] = $[Mn^{2+}]$ = 5.0 mM.

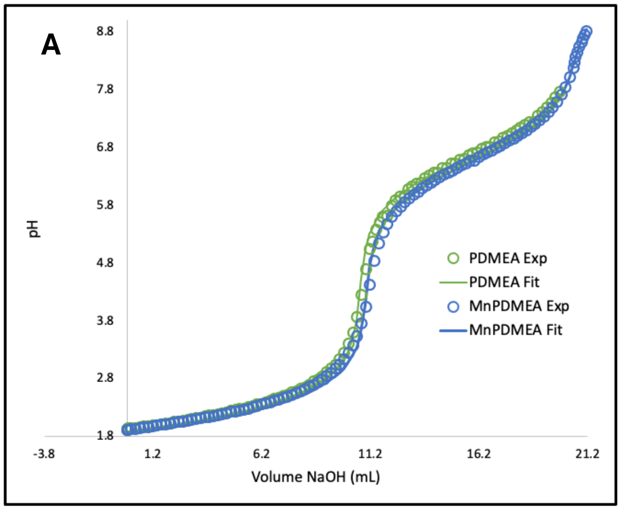

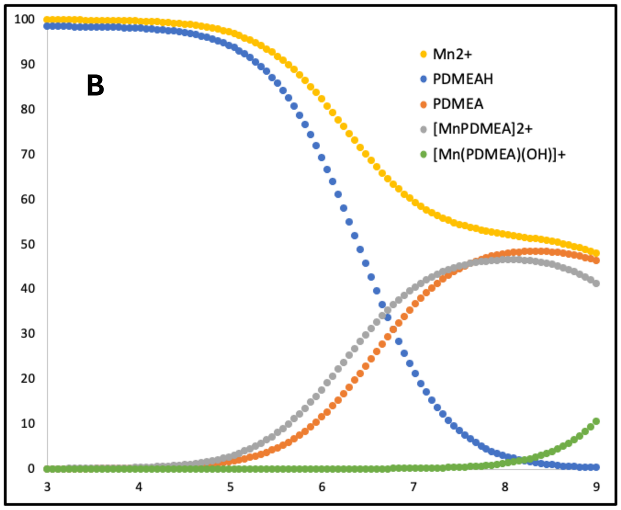

Figure S27. (A) Potentiometric titrations of 0.200 2-Methoxy-N-(2-methoxyethyl)-N-(pyridin-2-ylmethyl)ethan-1-amine (PDMEA) and 0.300 mmoles HCl in DI water containing 0.100 M KCl by 0.0500 M NaOH (green circles), and 0.200 mmoles PDMEA, 0.300 mmoles HCl, and 0.200 mmoles $MnCl_2$ in DI water containing 0.100 M KCl by 0.0500 M NaOH (blue circles). The protonation constant for PDMEA and stability constant for $[MnPDMEA(H_2O)_x]^{2+}$ complex formation were determined by a non-linear, least squares fitting (Hyperquad 2013[1]) of the potentiometric data (solid lines) to speciation models in which the PDMEA binds one proton and forms a mononuclear complex with manganese(II). The K_a of a coordinated water molecule is also accounted for in this model. (B) Speciation plot (HYSS[2]) produced from the protonation constant for the PDMEA ligand, stability constant (β_{MnL}) for the $[MnPDMEA(H_2O)_x]^{2+}$ complex, and K_a value of a coordinated water molecule, where [PDMEA] = $[Mn^{2+}]$ = 5.0 mM.

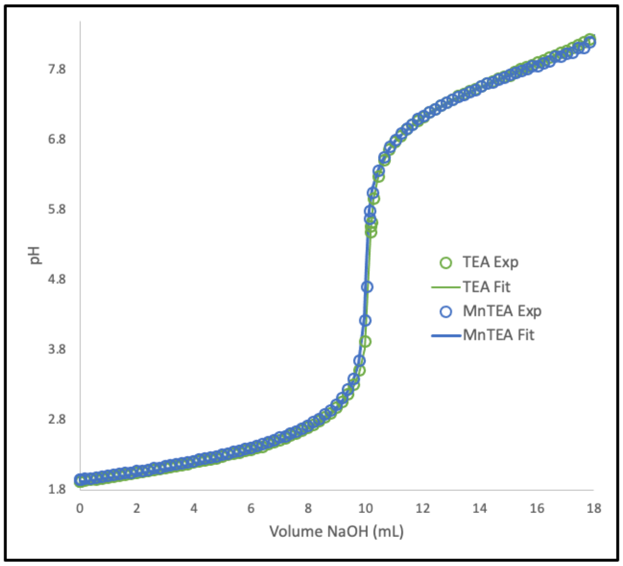

Figure S28. Potentiometric titrations of 0.500 mmoles Triethanolamine (TEA) and 1.00 mmoles HCl in DI water containing 0.100 M KCl by 0.0500 M NaOH (Green Circles), and 0.500 mmoles TEA, 1.00 mmoles HCl, and 0.500 mmoles $MnCl_2$ in DI water containing 0.100 M KCl by 0.0500 M NaOH (blue circles). The protonation constant for TEA was determined by a non-linear, least squares fitting (Hyperquad 2013[1]) of the potentiometric data (solid lines) to speciation models in which the TEA binds one proton. The presence of Mn^{2+} did not have an effect on the TEA titration indicating that complex formation does not occur under these conditions.

Cyclic voltammetry data

Table S36. Quantities Used for Recording Cyclic Voltammograms.

Ligand	Ligand (M)	$MnCl_2$ (M)	% Complex*
TMPA	0.001	0.001	93
DPEA	0.01	0.001	98
DPMEA	0.01	0.001	97
PDEA	0.01	0.001	66
PDMEA	0.01	0.001	61

* % Complex formation was estimated from complex stability studies herein.

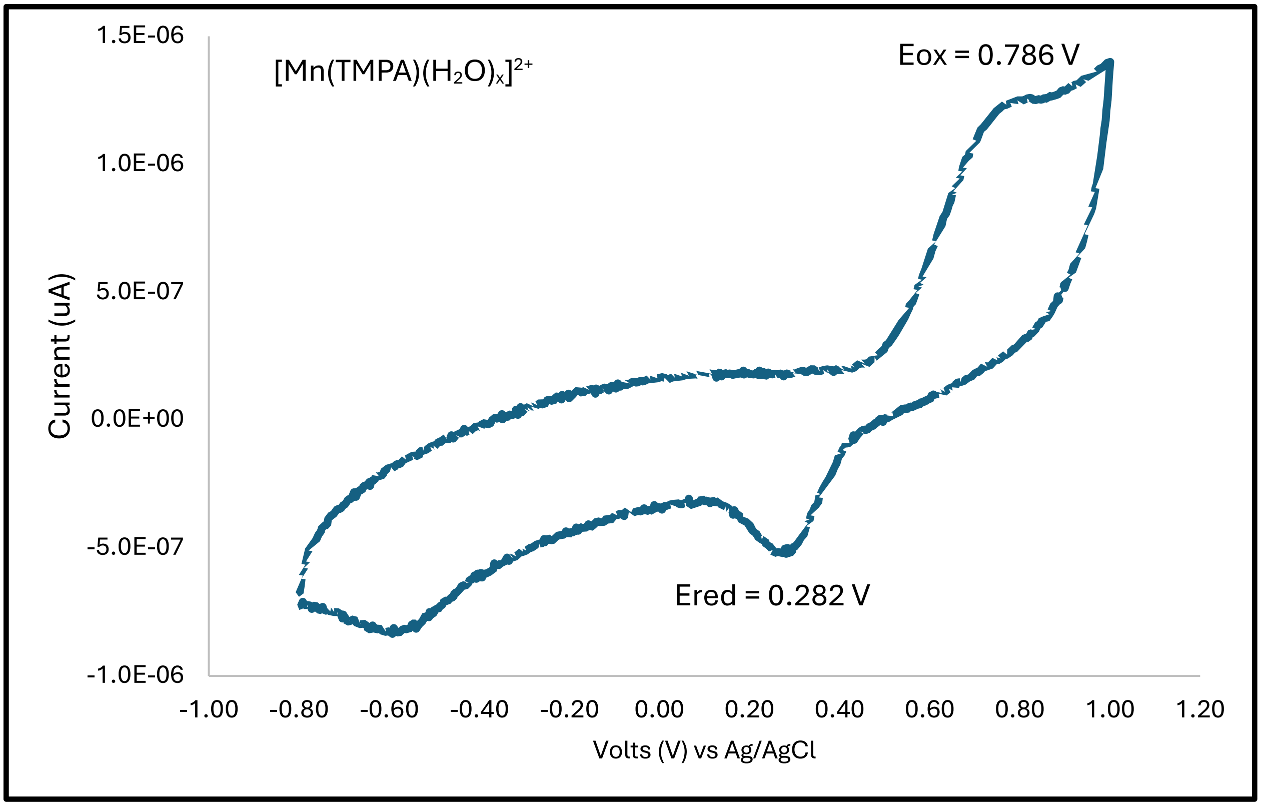

Figure S29. Cyclic Voltammogram of a solution of 0.001 M $MnCl_2$ and 0.001 M TMPA in 50 mM collidine buffer, pH 7.5. A glassy carbon working electrode, Ag/AgCl reference electrode, and Pt/Ti auxiliary electrode were employed. Prior to recording the CV, the solution was bubbled with nitrogen gas for 15 minutes to eliminate oxygen. The CV was then recorded from -0.6 to 1.1 V at a scan rate of 100 mV/s.

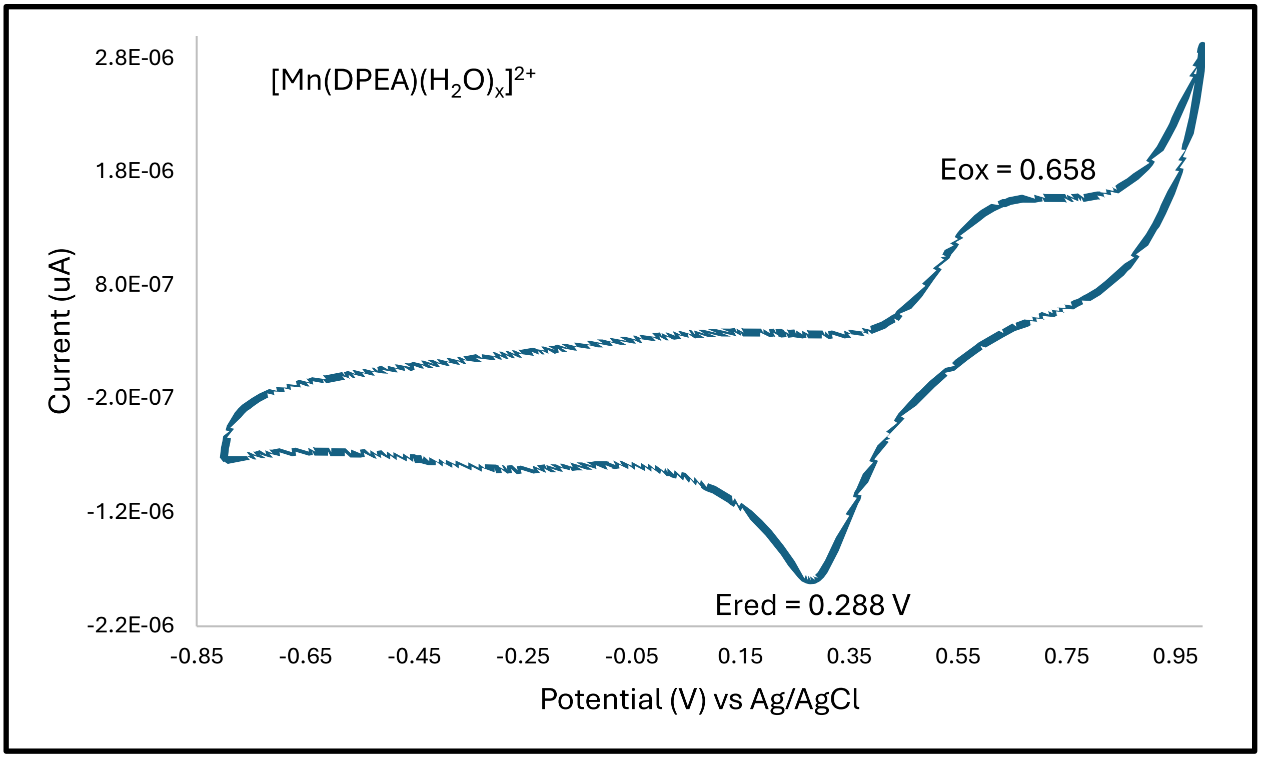

Figure S30. Cyclic Voltammogram of a solution of 0.001 M $MnCl_2$ and 0.01 M DPEA in 50 mM collidine buffer, pH 7.5. A glassy carbon working electrode, Ag/AgCl reference electrode, and Pt/Ti auxiliary electrode were employed. Prior to recording the CV, the solution was bubbled with nitrogen gas for 15 minutes to eliminate oxygen. The CV was then recorded from -0.85 to 0.95 V at a scan rate of 100 mV/s.

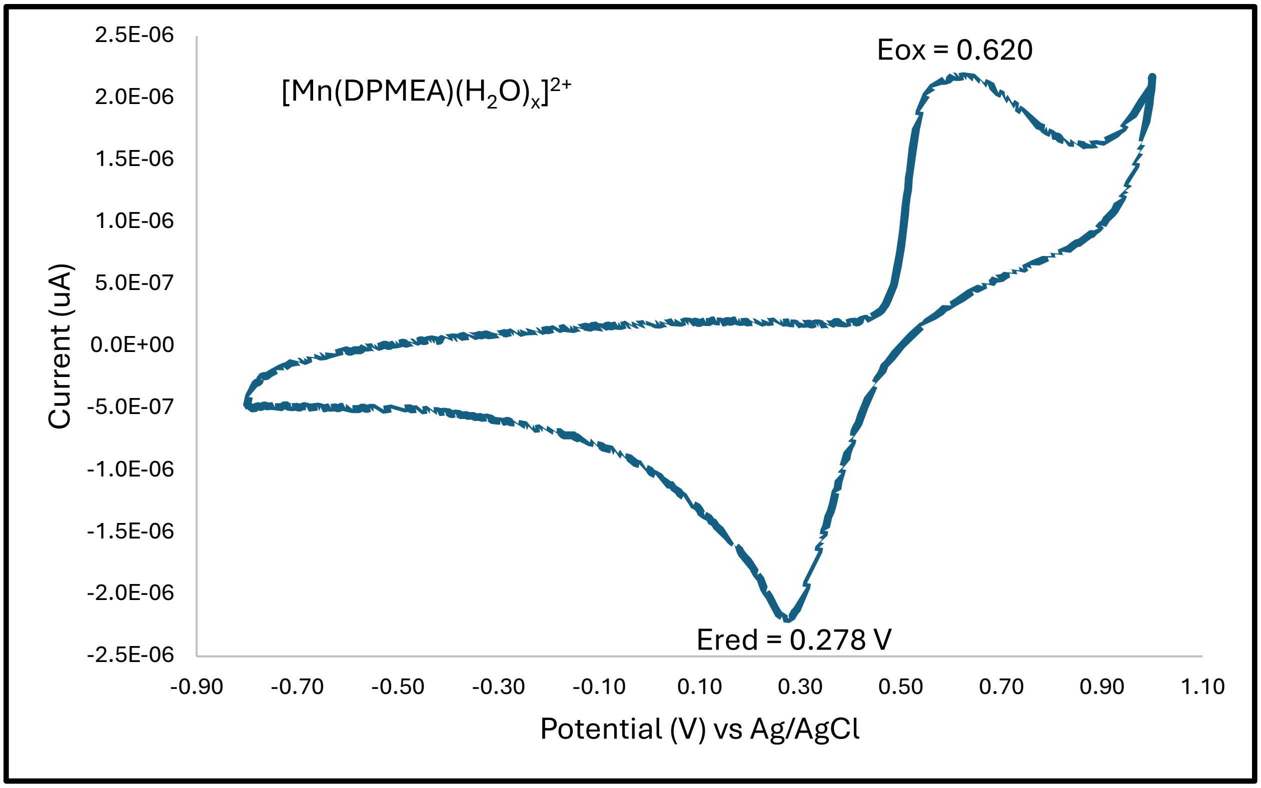

Figure S31. Cyclic Voltammogram of a solution of 0.001 M $MnCl_2$ and 0.01 M DPMEA in 50 mM collidine buffer, pH 7.5. A glassy carbon working electrode, Ag/AgCl reference electrode, and Pt/Ti auxiliary electrode were employed. Prior to recording the CV, the solution was bubbled with nitrogen gas for 15 minutes to eliminate oxygen. The CV was then recorded from -0.9 to 1.1 V at a scan rate of 100 mV/s.

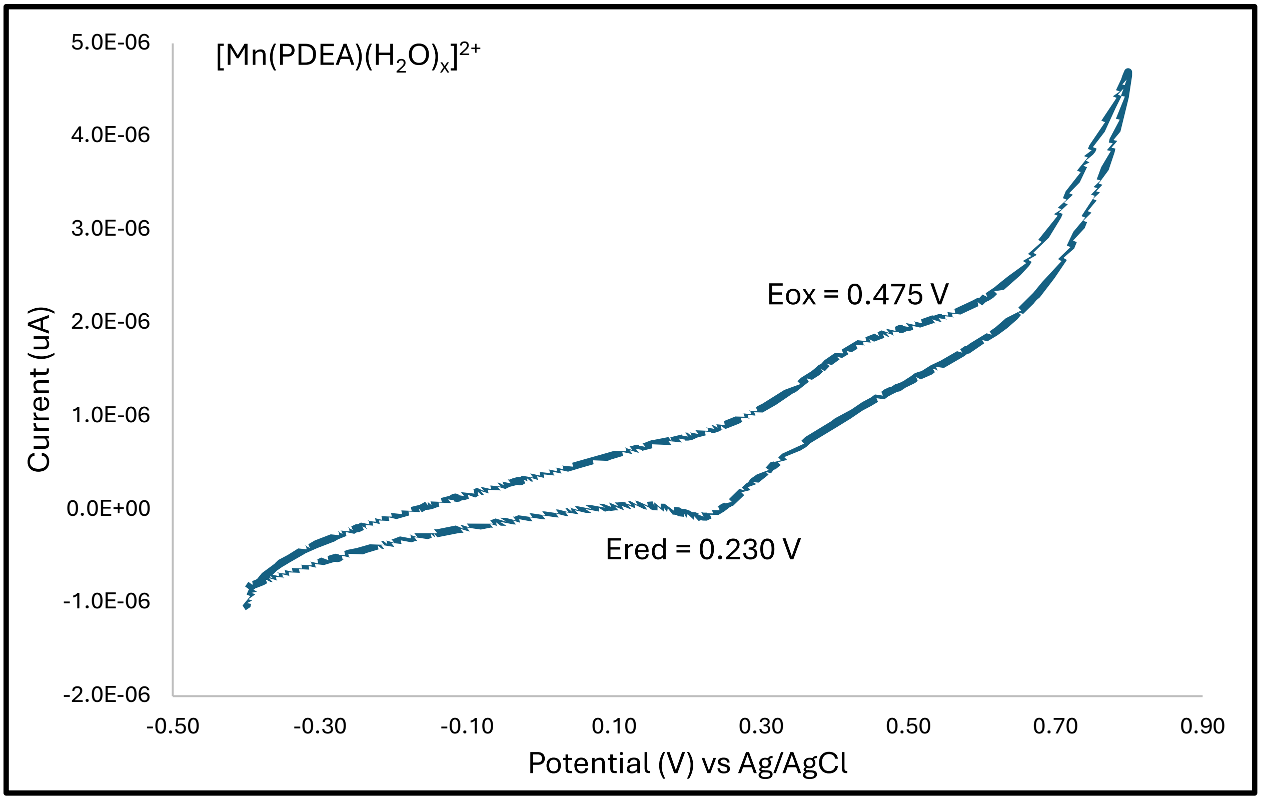

Figure S32. Cyclic Voltammogram of a solution of 0.001 M $MnCl_2$ and 0.001 M PDEA in 50 mM collidine buffer, pH 7.5. A glassy carbon working electrode, Ag/AgCl reference electrode, and Pt/Ti auxiliary electrode were employed. Prior to recording the CV, the solution was bubbled with nitrogen gas for 15 minutes to eliminate oxygen. The CV was then recorded from -0.4 to 0.8 V at a scan rate of 100 mV/s.

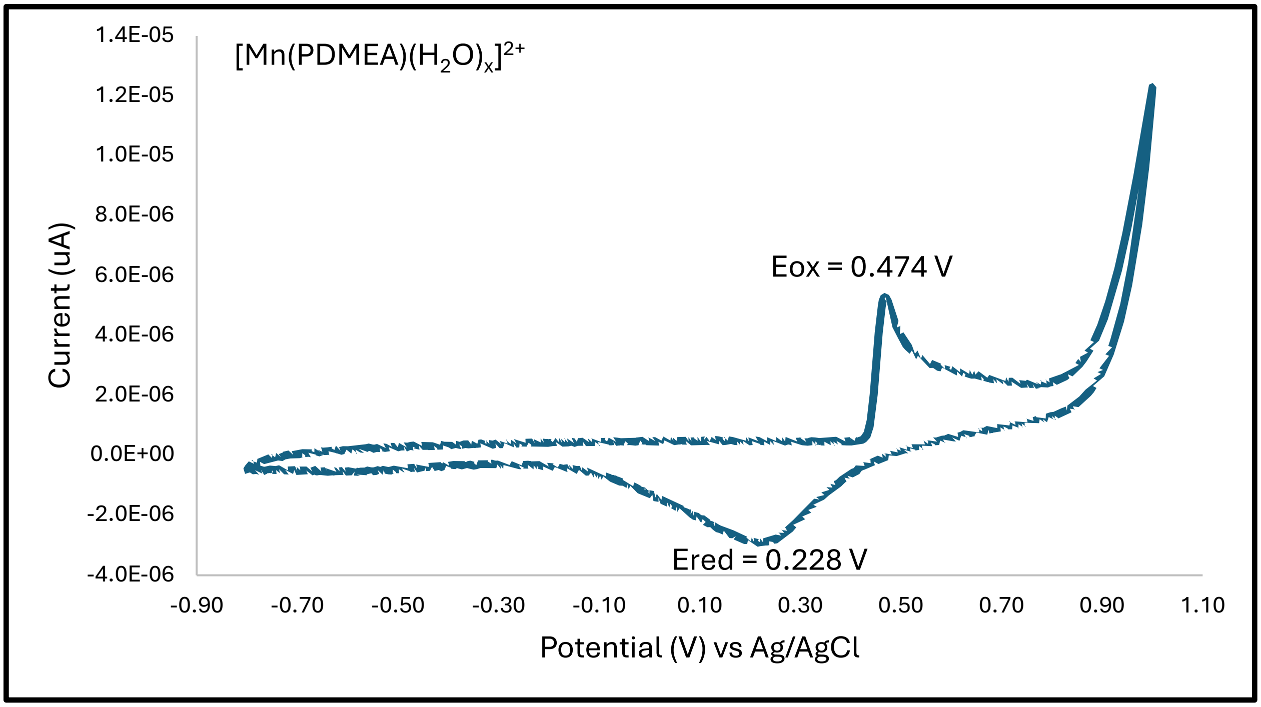

Figure S33. Cyclic Voltammogram of a solution of 0.001 M $MnCl_2$ and 0.001 M PDEA in 50 mM collidine buffer, pH 7.5. A glassy carbon working electrode, Ag/AgCl reference electrode, and Pt/Ti auxiliary electrode were employed. Prior to recording the CV, the solution was bubbled with nitrogen gas for 15 minutes to eliminate oxygen. The CV was then recorded from -0.8 to 1.0 V at a scan rate of 100 mV/s.

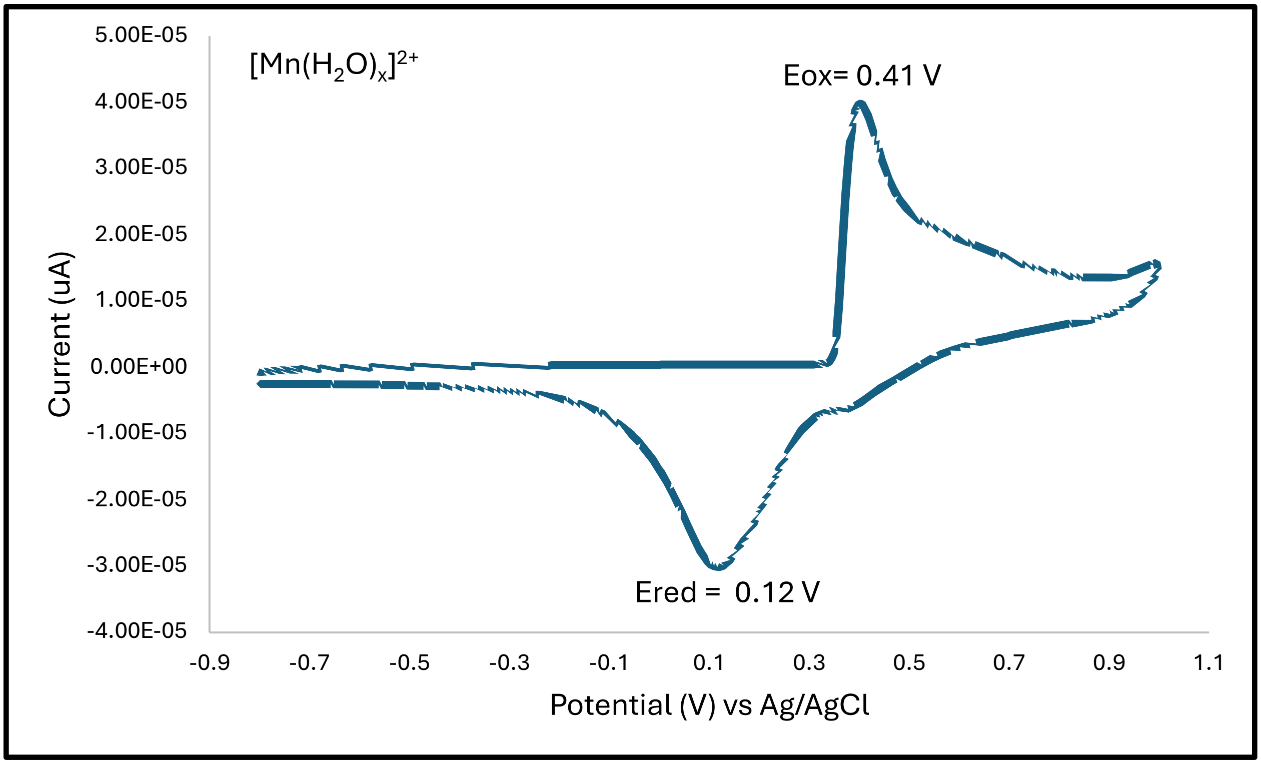

Figure S34. Cyclic Voltammogram of a solution of 0.001 M $MnCl_2$ in 50 mM collidine buffer, pH 7.5. A glassy carbon working electrode, Ag/AgCl reference electrode, and Pt/Ti auxiliary electrode were employed. Prior to recording the CV, the solution was bubbled with nitrogen gas for 15 minutes to eliminate oxygen. The CV was then recorded from -0.8 to 1.0 V at a scan rate of 100 mV/s.

McCord Fridovich Assay Data

Table S37. Quantities Used for McCord-Fridovich assays.

Ligand	Ligand (M)	% Complex*
TMPA	0.001	99
DPEA	0.001	92
DPMEA	0.001	80
PDEA	0.01	66
PDMEA	0.01	61

* % Complex formation was estimated from complex stability studies herein, given an $MnCl_2$ concentration of 0.1 μM.

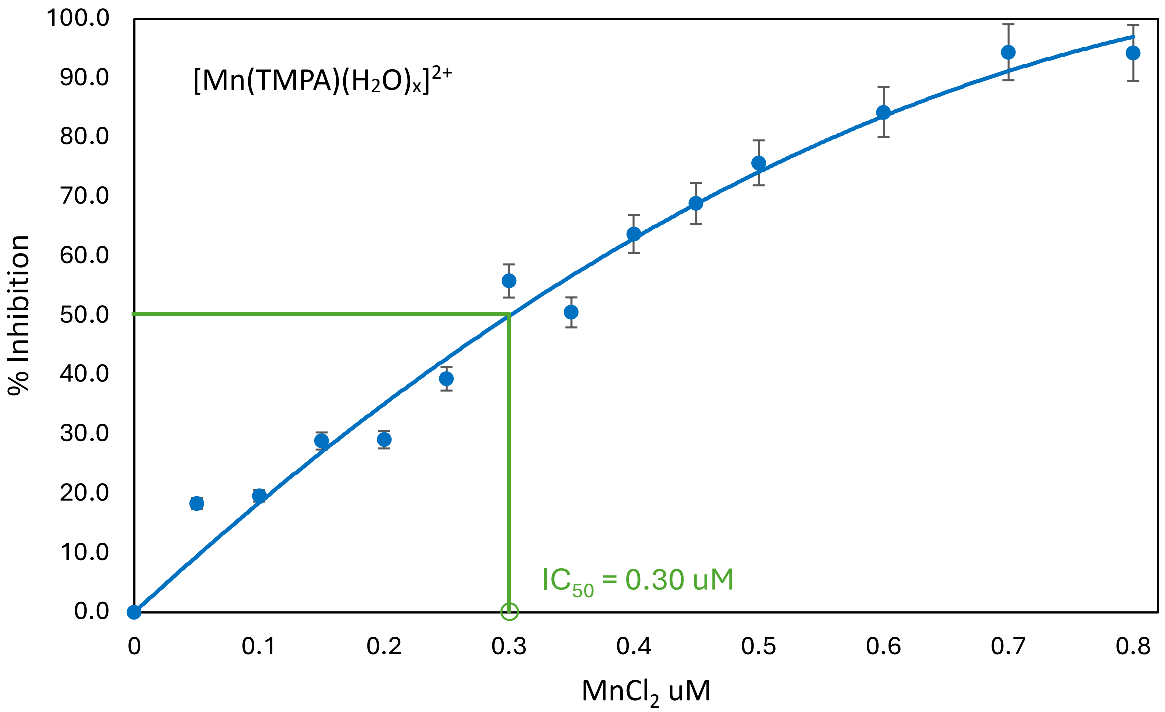

Figure S35. McCord-Fridovich assay plot of percent inhibition versus the concentration of $MnCl_2$ for experiments carried out in 50 mM HEPES buffer, pH 7.5, containing 0.001 M TMPA. Other reagents included 50 μM cytochrome C, 50 μM xanthine, 30 μg/mL catalase, and 100 μg/mL xanthine oxidase, along with enough buffer to provide a total of 3 mL of solution. Experiments were run in triplicate at 25°C, and error bars represent the standard deviations.

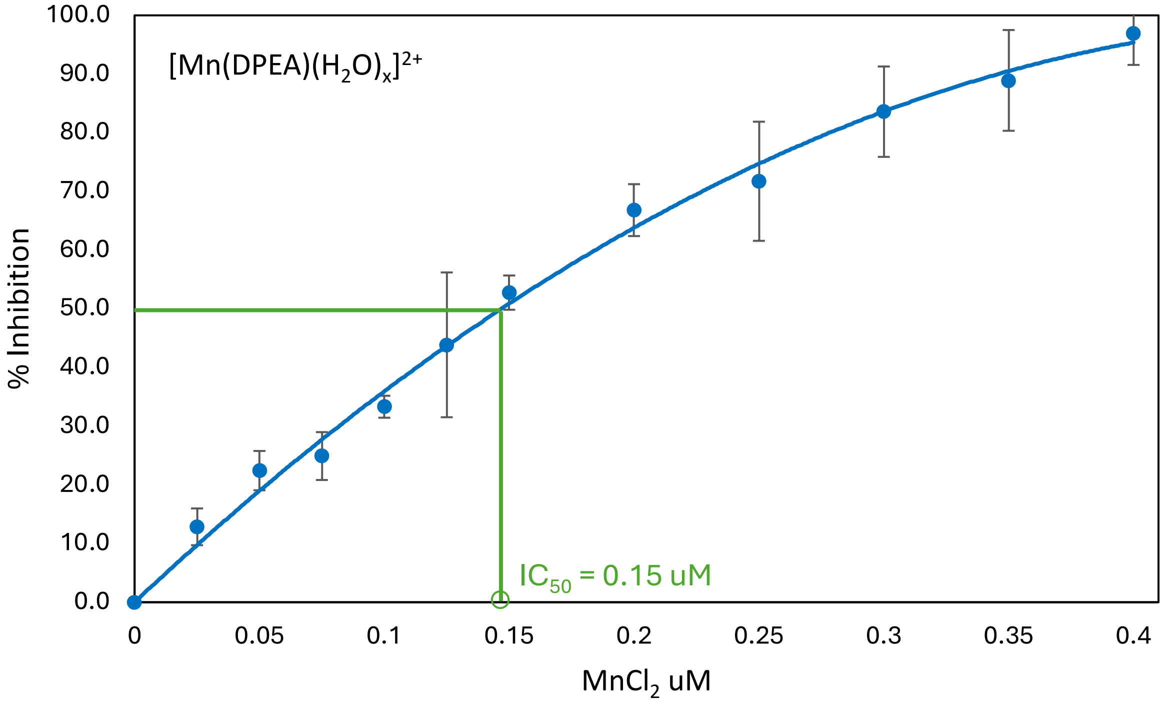

Figure S36. McCord-Fridovich assay plot of percent inhibition versus the concentration of $MnCl_2$ for experiments carried out in 50 mM HEPES buffer, pH 7.5, containing 0.01 M DPEA. Other reagents included 50 μM cytochrome C, 50 μM xanthine, 30 μg/mL catalase, and 100 μg/mL xanthine oxidase, along with enough buffer to provide a total of 3 mL of solution. Experiments were run in triplicate at 25°C, and error bars represent the standard deviations.

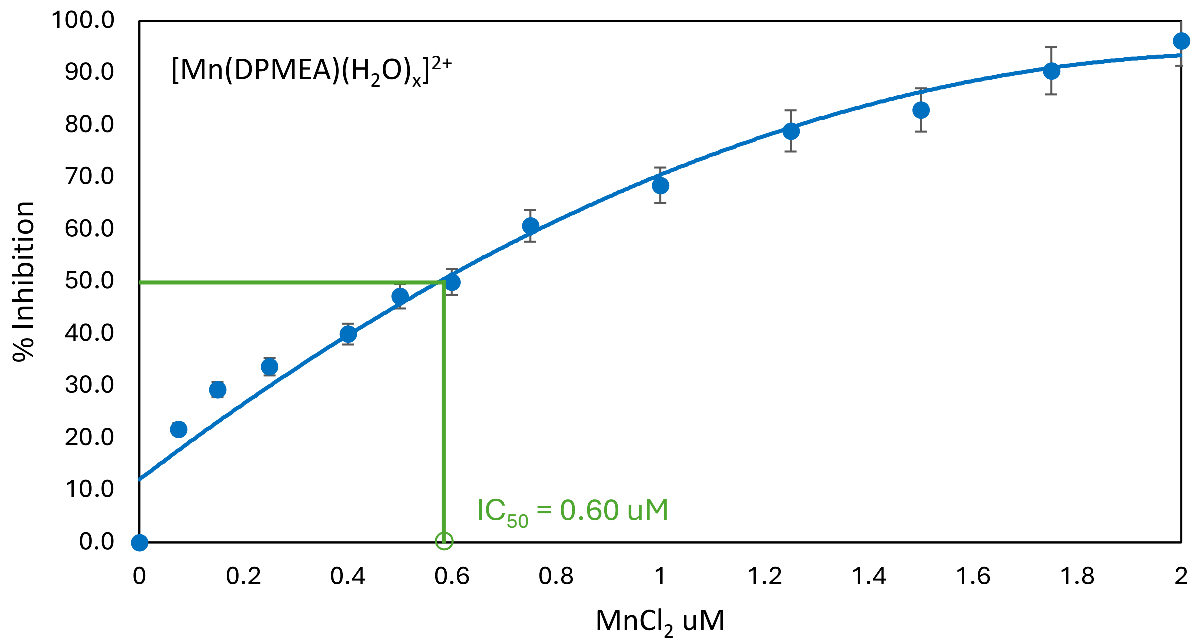

Figure S37. McCord-Fridovich assay plot of percent inhibition versus the concentration of $MnCl_2$ for experiments carried out in 50 mM HEPES buffer, pH 7.5, containing 0.01 M DPMEA. Other reagents included 50 μM cytochrome C, 50 μM xanthine, 30 μg/mL catalase, and 100 μg/mL xanthine oxidase, along with enough buffer to provide a total of 3 mL of solution. Experiments were run in triplicate at 25°C, and error bars represent the standard deviations.

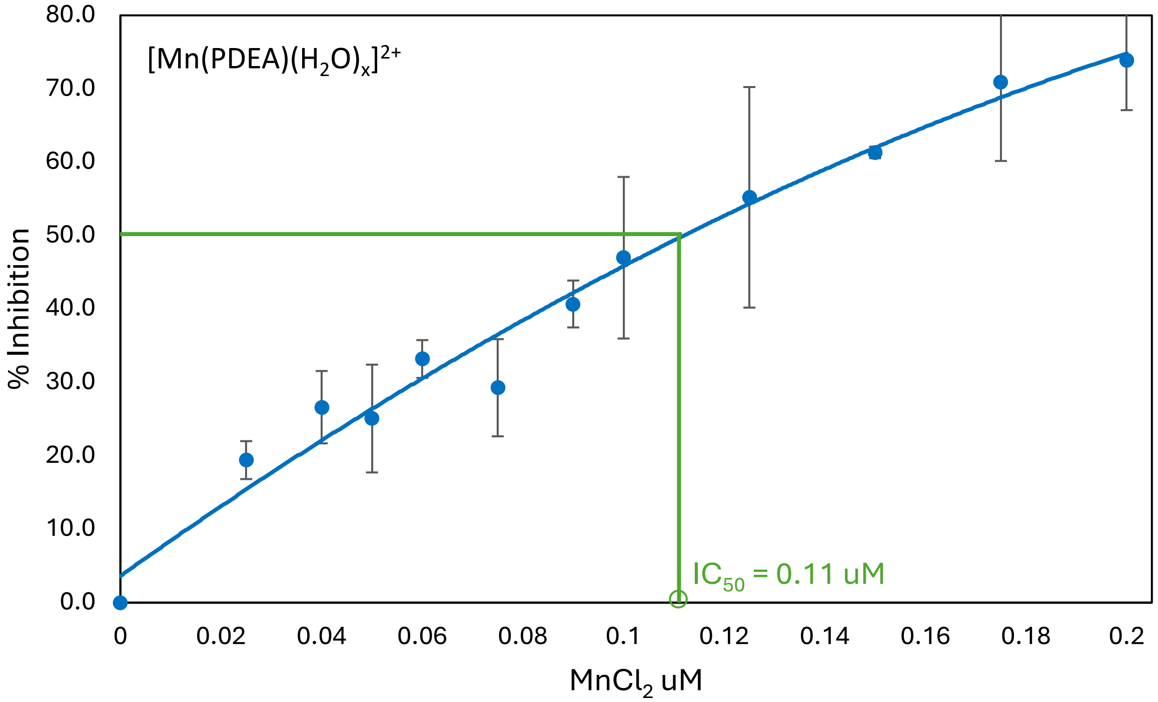

Figure S38. McCord-Fridovich assay plot of percent inhibition versus the concentration of $MnCl_2$ for experiments carried out in 50 mM HEPES buffer, pH 7.5, containing 0.01 M PDEA. Other reagents included 50 μM cytochrome C, 50 μM xanthine, 30 μg/mL catalase, and 100 μg/mL xanthine oxidase, along with enough buffer to provide a total of 3 mL of solution. Experiments were run in triplicate at 25°C, and error bars represent the standard deviations.

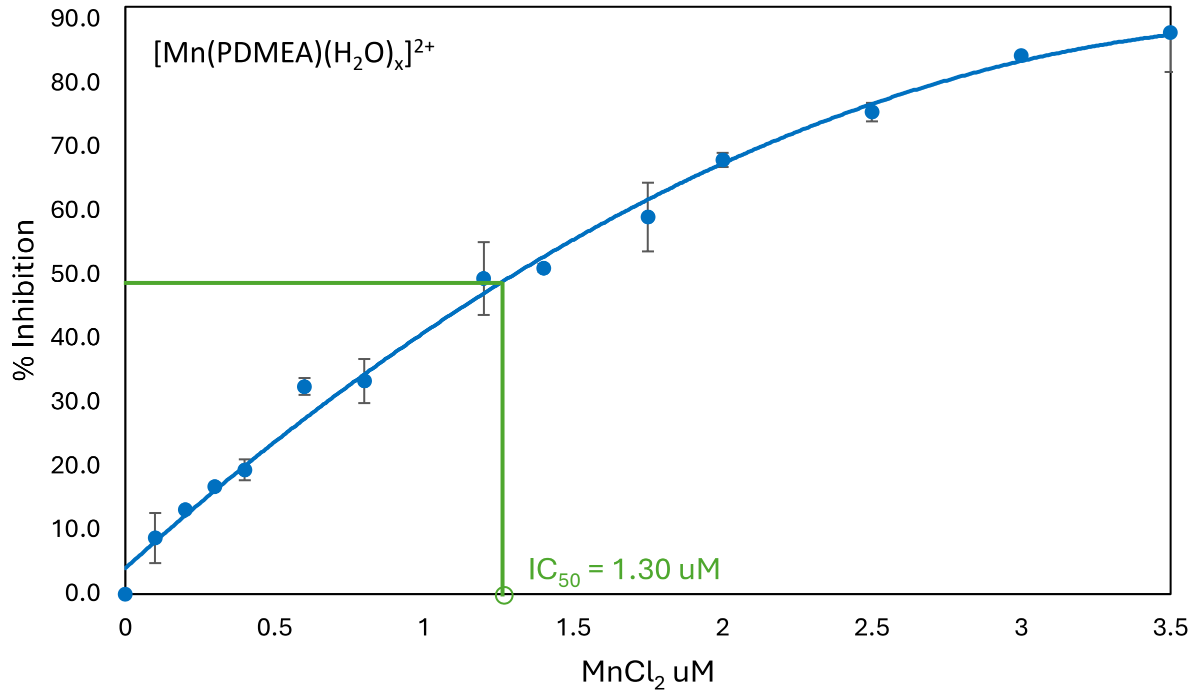

Figure S39. McCord-Fridovich assay plot of percent inhibition versus the concentration of $MnCl_2$ for experiments carried out in 50 mM HEPES buffer, pH 7.5, containing 0.01 M PDMEA. Other reagents included 50 μM cytochrome C, 50 μM xanthine, 30 μg/mL catalase, and 100 μg/mL xanthine oxidase, along with enough buffer to provide a total of 3 mL of solution. Experiments were run in triplicate at 25°C, and error bars represent the standard deviations.

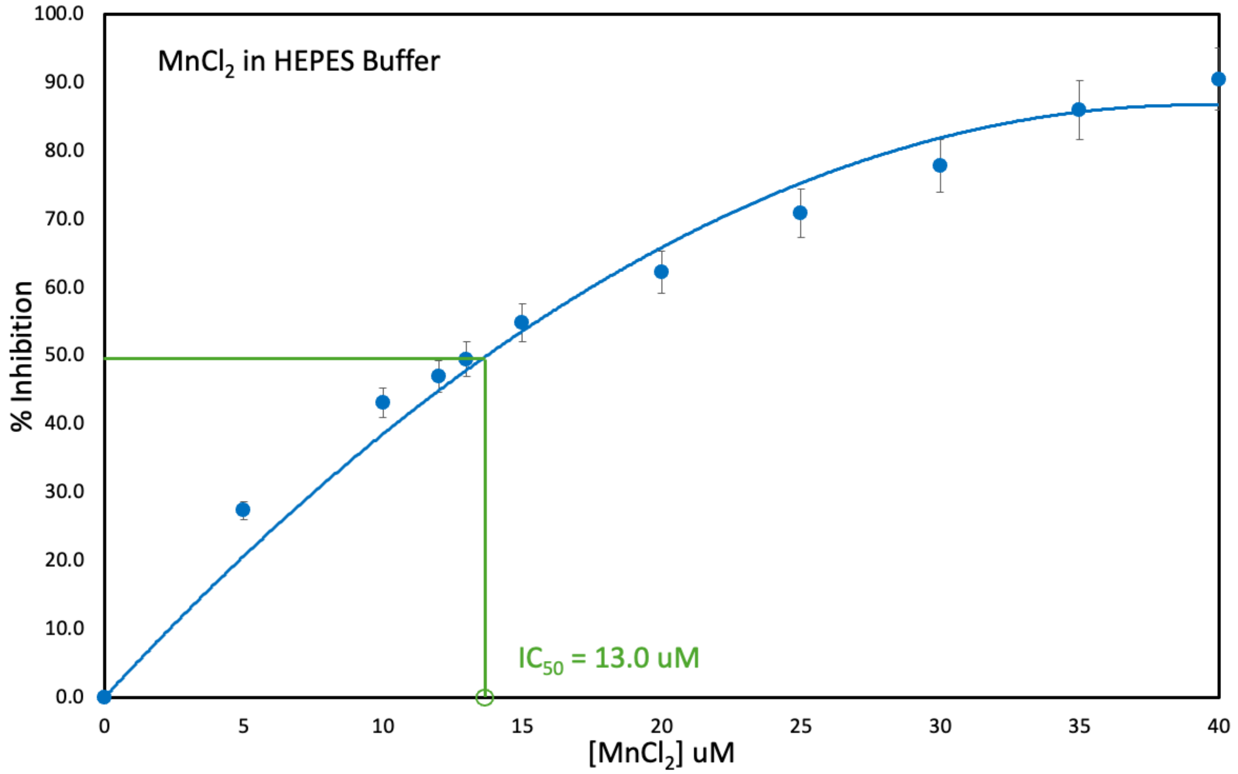

Figure S40. McCord-Fridovich assay plot of percent inhibition versus the concentration of $MnCl_2$ for experiments carried out in 50 mM HEPES buffer, pH 7.5. Other reagents included 50 μM cytochrome C, 50 μM xanthine, 30 μg/mL catalase, and 100 μg/mL xanthine oxidase, along with enough buffer to provide a total of 3 mL of solution. Experiments were run in triplicate at 25°C, and error bars represent the standard deviations.

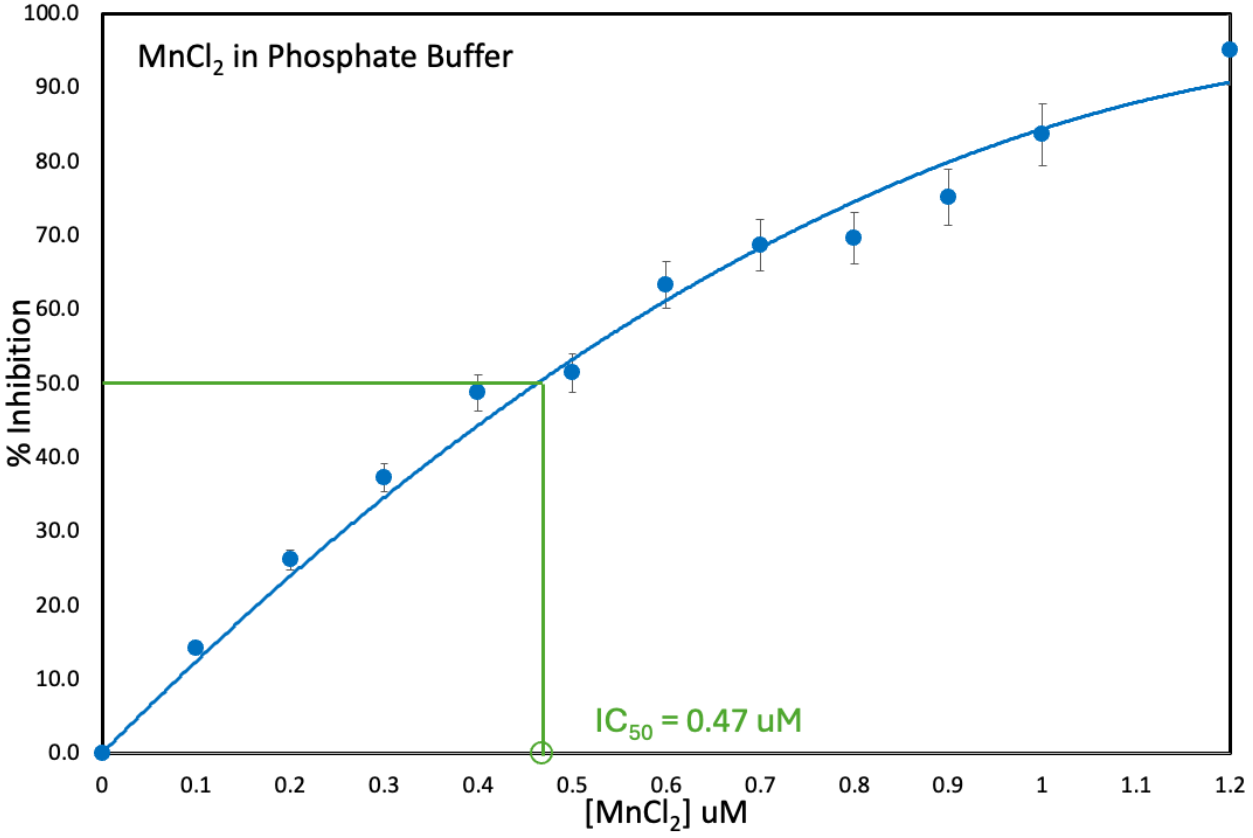

Figure S41. McCord-Fridovich assay plot of percent inhibition versus the concentration of $MnCl_2$ for experiments carried out in 50 mM Phosphate buffer, pH 7.5. Other reagents included 50 μM cytochrome C, 50 μM xanthine, 30 μg/mL catalase, and 100 μg/mL xanthine oxidase, along with enough buffer to provide a total of 3 mL of solution. Experiments were run in triplicate at 25°C, and error bars represent the standard deviations.
